# Longitudinal Prediction of the Infant Gut Microbiome with Dynamic Bayesian Networks

**DOI:** 10.1038/srep20359

**Published:** 2016-02-08

**Authors:** Michael J. McGeachie, Joanne E. Sordillo, Travis Gibson, George M. Weinstock, Yang-Yu Liu, Diane R. Gold, Scott T. Weiss, Augusto Litonjua

**Affiliations:** 1 Channing Division of Network Medicine, Brigham and Women’s Hospital, Boston, MA; 2 The Jackson Laboratory for Genomic Medicine, Farmington, CT

## Abstract

Sequencing of the 16S rRNA gene allows comprehensive assessment of bacterial community composition from human body sites. Previously published and publicly accessible data on 58 preterm infants in the Neonatal Intensive Care Unit who underwent frequent stool collection was used. We constructed Dynamic Bayesian Networks from the data and analyzed predictive performance and network characteristics. We constructed a DBN model of the infant gut microbial ecosystem, which explicitly captured specific relationships and general trends in the data: increasing amounts of *Clostridia*, residual amounts of *Bacilli*, and increasing amounts of *Gammaproteobacteria* that then give way to *Clostridia*. Prediction performance of DBNs with fewer edges were overall more accurate, although less so on harder-to-predict subjects (p = 0.045). DBNs provided quantitative likelihood estimates for rare abruptions events. Iterative prediction was less accurate (p < 0.001), but showed remarkable insensitivity to initial conditions and predicted convergence to a mix of *Clostridia, Gammaproteobacteria*, and *Bacilli*. DBNs were able to identify important relationships between microbiome taxa and predict future changes in microbiome composition from measured or synthetic initial conditions. DBNs also provided likelihood estimates for sudden, dramatic shifts in microbiome composition, which may be useful in guiding further analysis of those samples.

## Microbiome

The microbiota living in the human gut performs a number of vital functions for homeostasis, including the harvest of essential nutrients^1^,^2^, synthesis of vitamins^3^, metabolism of xenobiotics^4^, and the development and maintenance of the immune system^5^,^6^. Alterations in the gut microbiome have been observed in a number of disease states^7^, and may be directly connected to pathogenesis. Microbes that populate an infant’s gut after birth serve as critical immune stimuli in the first days of life^8^, and could influence the composition of the “mature” gut microbiome, with subsequent implications for the health of the human host in both early life and adulthood.

Studies on the initial colonization of the infant gut are very limited, with sparse, if any, longitudinal data^1^. Early studies have proposed an initial predominance of facultative anaerobes, followed by a progression to anaerobic bacteria^9^,^10^. The “first colonizers” of the infant gut may derive from maternal sources (vaginal flora, skin flora, and gut flora) or from environmental microbes. Microbiome studies of meconium^11^, amniotic fluid and placenta^12^, suggest that infants encounter microbes even before birth. Microbial rDNA present in the intrauterine environment suggests that prenatal sources may also contribute to gut colonization^12^.

The most comprehensive study on the progression of the infant gut microbiota thus far examined 58 preterm infants in a neonatal intensive care unit, with repeated measurements taken every few days on all study subjects starting within the first days of life, and ending at approximately one month of age^13^. In this study, La Rosa *et al.* used longitudinal analysis of time series data to demonstrate that the microbiota of the infant gut was initially dominated by *Bacilli* at birth, giving way to *Gammaproteobacteria*, then *Clostridia* at the end of the first month of life. Gestational age appeared to have the greatest influence on the pace (but not the pattern) of bacterial microbiome progression, and the non-random assembly observed seems to suggest that host biology or consistent exposure sources (*i.e.* infant diet, maternal flora) play a key role in infant gut population (as compared to chance encounters with microbes in the environment).

Although classic longitudinal analysis captures changes in outcomes over time, this standard approach has many limitations. Often, individual taxa are treated as separate outcomes, and information on the connections between bacteria (i.e. how one bacterial population may influence another over time) is lost. Network-based methods are an alternative approach to longitudinal gut microbiome modeling. In general, network studies on gut microbiota have been limited thus far; most are comprised of correlation analyses, which others have noted have poor asymptotic prediction^14^. Two types of network methodologies specifically designed to capture complex interactions and dynamic change within the microbiome over time include the generalized Lotka-Volterra model and Dynamic Bayesian Networks.

Generalized Lotka-Volterra (GLV) model and other dynamic systems identification formalisms^15^ use longitudinal microbiota compositional data to identify parameters in ordinary differential equations that describe the dynamics of microbial ecosystems^16^,^17^. For instance, GLV has been used in a study of the murine gut microbiome to generate a network of interactions between bacterial taxa^18^; this network included almost all possible edges and was not used for prediction. In another microbiome study, a continuous GLV model was assumed and coefficients related to the individual microbe growth rates, the strengths of the microbe-microbe interactions, and susceptibility to antibiotics were learned using linear regression with regularization^17^. Discrete GLV, where coefficients were learned using a sparse linear regression technique, has also been employed^16^. Alternatively, other dynamic systems models have been used, in one case modeling two of possibly many interacting microbes in the gut^19^. These endeavors build upon a rich history of systems-identification literature, spanning the theoretical and practical^20^, and these approaches have shown that data-derived models of microbiota dynamics can have significant analytic and predictive power^17^. However, the degree to which the microbiome datasets available meet the rigorous requirements of exact parameter estimation in these models remains an outstanding question^16^. Methods that include explicit parameter estimation and allowances for noisy data may be more appropriate. One such method used Bayesian statistics to help inform dynamic models of a single independent bacteria taxon’s change in response to antibiotics^21^.

Bayesian networks (BNs) are an appropriate tool to model the interaction of many microbial taxa in the gut microbiome, since they include implicit parameter estimation techniques for inferring complex networks from noisy data and are intended to predict clinical outcomes of relevance. In this work we model the infant gut microbiome using Dynamic Bayesian Networks (DBN), which are related to (static) Bayesian Networks (BN), but are ideal for modeling biological systems with feedback loops, which traditional BNs cannot easily represent^22^. DBNs have been successfully used to model time-series data in other clinical and ecological applications, including bird populations in wildlife reserves^23^, cancer outcomes^24^, gene regulatory networks from transcriptome data in blood^25^ and from perturbation data^26^. In these applications DBNs were found to be superior to other techniques including LASSO regression^23^ and proportional hazards^24^ for outcome prediction. To our knowledge, DBNs have not yet been applied to human microbiome data.

In this paper, we employed DBNs to model the progression of microbiota in colonizing the infant gut, using microbiota abundance data from the previously published La Rosa *et al.* study^13^. Using DBNs, we developed a predictive model for the infant’s gut microbiome based on prior composition. We modeled relationships between multiple bacterial taxa (both dominant and rare), the compositional changes bacterial taxa exert on other community members over time, and the influence of potential external characteristics (mode of delivery, antibiotic use, etc) on gut microbiome progression. We also investigated the likelihood of abruption events (rapid, extreme changes in microbiome composition), and determined how these abruptions influence future taxonomic composition in the infant’s gut.

## Methods

### Microbiome Data and Clinical Characteristics

We obtained public microbiome data collected by La Rosa *et al.*^13^ for their paper *Patterned progression of bacterial populations in the premature infant gut* from the PNAS website. This data is described in greater detail in La Rosa *et al.*^13^; briefly, it contains 922 infant gut microbiome measurements from a total of 58 pre-term infants, taken one or two days apart on average. Infants enrolled in the study were ≤1500 g at birth and were expected to live beyond the first week of life. Infants that experienced serious intra-abdominal pathology (i.e. necrotizing enterocolitis) were excluded from the study. Demographic and clinical data collected on the preterm infants included race, gender, gestational age at birth, post conceptional age when the stool sample was obtained, being housed in an open or closed room, dietary information (breast milk as a percentage of total enteral volume administered to the infant), mode of delivery (C-section vs. vaginal), and antibiotic use (percent of days of life on antibiotic). To determine the bacterial community composition of the infant stool samples, the 16S region (V3-V5) of bacterial rDNA was sequenced using the Roche 454 platform. The median number of 16S rDNA sequence reads across all infant stool specimens collected was 7,183 (IQR 5,243–9,314). Clinical and demographic data, along with counts of 16S bacterial ribosomal DNA sequences grouped by bacterial class, is contained in the file “pnas.1409497111.sd01.xlsx”. From that file, we retained all bacteria, although two of the bacterial taxa (Cyanobacteria and Holophagae) are likely environmental contaminants, in the spirit of using all available data for model construction. Using all of the pre-term infant gut microbiome data, we constructed dynamic Bayesian networks to capture the influence of individual microbial classes on each other over time. In short, longitudinal data (repeated measures of the microbial classes) are accounted for within the dynamic Bayes net, and the connections between nodes show how one taxon may influence another over time. Our confidence in each individual network connection is reflected by the Bayes Factor; connections with the largest Bayes factors are more likely to represent a true causal association. Details of dynamic Bayes network methodology are shown below.

### Dynamic Bayesian Networks

We used the CGBayesNets package^27^ to build two-stage dynamic Bayesian networks of the microbiome population dynamics from the entire data set. We use “two-stage” here to refer to the use of an abstraction on time points: that the network model only considers “current time” samples and the immediately previous time samples. The CGBayesNets package performs both network learning and inference with discrete and continuous (normal) nodes in a formalism known as a Conditional Gaussian Bayesian network^28^. We adapted this to construct two-stage DBNs. For each sample, bacterial ribosomal DNA sequence counts were converted to relative abundances (proportions). To satisfy requirements of a two-stage dynamic Bayesian network, each microbial sample was paired with the immediately preceding microbial sample from the same subject. This resulted in 864 paired microbial samples, used as the primary data for the DBN modeling.

The two-stage DBN was constructed by using standard Bayesian network techniques, but limiting edges to only those going from prior time points to subsequent time points. In preliminary analysis, the variables “mode of birth” referring to cesarean or vaginal delivery, “milk” indicating the amount of breastfeeding the infant received, and “gender”, resulted in overfitting. Including these variables in the modeling step led to networks with these three variables linked to all of the bacteria (although not *Gammaproteobacteria*), which is a sign of overfitting. We assessed the degree of overfitting by five-fold cross validation, building the network on 4/5^th^s of the subjects and testing it on the remaining 1/5^th^, in the same manner as described below; where networks using these variables had on average greater per-person error (0.0325 vs. 0.0296, p = 0.009, two-tailed t-test), indicating a reduced ability to make generalizations from the majority of subjects that would then predict bacteria concentrations of the held out minority. We removed these variables from further investigation. Clinical variables that remained in the DBN model were days of antibiotics, day of life of sample obtained, gestational age at birth, and post-conceptional age when sample obtained; and also including the binary indicator for the infant being cared for in an open (double) or closed (single) room .

While building our Bayesian networks, prior assumed distributions on each node are required for use with Bayes’ Rule to determine the posterior probability of the data; the parameters of these prior distributions were chosen to provide a strong complexity penalty (prior equivalent sample size: ν = 10; prior assumed standard deviation: σ^2^ = 1; for more details, see McGeachie *et al.*^28^) and limited each node to a maximum of three possible parent nodes (although with an unlimited number of child nodes). We used the CGBayesNet function FullBNLearn()^27^, which performs an exhaustive search through possible edges using a hill-climbing algorithm: starting with an empty network with no edges, it chooses the most likely edge addition (or deletion), adds that edge to the network, and then repeats this process until no more edges remain which will increase the posterior likelihood of the data given the network^28^. We predicted the progress of the microbiome of each subject from the DBN using the remaining subjects as training data to learn the parameters of the DBN, thus not biasing the network with the microbiome transitions unique to a particular subject.

**Figure 1:**
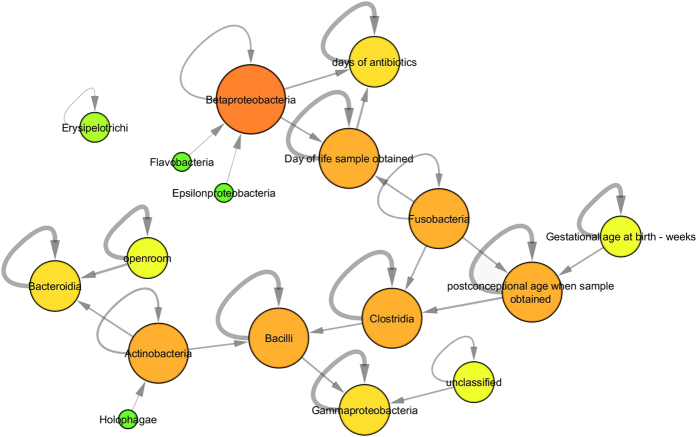
Dynamic Bayesian Network of Infant Gut Microbiota Dynamics. The DBN network included the clinical variables and bacteria taxa pictured above. Nodes indicate variables, with greater size and redder color indicating greater node degree. Direction of edges indicate temporal statistical influence: the source node’s prior value predicts the target node’s present value. Edge thickness indicates strength of statistical dependence.

For comparison, we built a DBN using a strong prior (parameter weights ν = 50, σ^2^ = 1), which biased toward Gaussians with large standard deviations compared to the data, and set the maximum number of parents per node to five; these settings result in more edges being included in the network. We compared predictive performance of this network (the “dense” network, supplemental Fig. 1) to our preferred network (the “sparse” network, Fig. 1) on each of the subjects. We compared performance differences in mean absolute error using a two-tailed t-test.

To identify unlikely abruption events, which signify departures from the underlying, expected model, we calculated the distribution of posterior log probabilities of each sample, on the –log10 scale, then converted to z-scores, and identified outliers that had a p-value less than 0.05/58. Since the dataset does not contain purely independent observations, we chose the number of subjects (n = 58) as the Benjamini-Hochberg correction for multiple testing; this enabled us to identify subjects experiencing at least one statistically unlikely abruption event.

### Iterative Models

DBNs provide a way of simulating the action of perturbations or unusual initial conditions on the eventual microbiome composition. We performed such iterative predictions by starting with initial conditions similar to a particular subject, and then used those values to predict the bacteria abundances of the second sample for that subject. We then used those predictions to predict the third sample’s abundances, and then used that output to predict the fourth, and so on. This allowed us to project microbiome abundances far into the future and to simulate unusual initial conditions.

### Model Construction

We measured accuracy of the model in predicting each time point from the previous time point, for each subject. Using all available variables, we obtained predictions for the amount of each bacterial taxa included in the network. These values were then normalized to represent percent composition of bacteria out of the total population, in keeping with the original interpretation of these data. In further analysis, bacteria taxa were included if they either appeared in the DBN model or represented at least 1% of any one sample, we thus included *Actinobacteria, Alphaproteobacteria, Bacilli, Bacteroidia, Betaproteobacteria, Clostridia, Cyanobacteria, Epsilonproteobacteria, Erysipelotrichi, Flavobacteria, Fusobacteria, Gammaproteobacteria, Holophagae*, and unclassified. Average predictive accuracy was measured by mean absolute error across the included bacteria taxa. For clarity, figures are shown displaying the 12 most commonly-occurring bacteria taxa.

### Formal Bayesian Methodology

Our DBNs are a type of Conditional Gaussian Bayesian model; and as such it induces a joint distribution over the variables in the domain of the time series, a distribution which is a multidimensional normal mixture density. The DBN is composed of a directed acyclic graph *G* over the variables of the domain (discrete variables Δ in the present analysis, including *open room*; and continuous variables *Ψ,* including all of the bacteria concentrations and *antibiotics, day of life sample obtained, gestational age at birth, post-conceptual age when sample obtained*). We write *π(X)* for the set of parents of variable *X* in *G*. The DBN further specifies a set of conditional probability distributions *P* over Δ, and a set of conditional linear Gaussian density functions *F* over *Ψ*. We can then write the multivariate normal mixture density over all variables as:


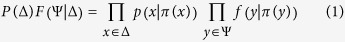


Continuous variables are modeled as Gaussian regressions on their continuous parents with parameters dependent upon their discrete parents, so that we have:


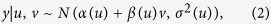


for *u* the discrete parents of *y* and *v* the continuous parents of *y*, according to the DBN, where α(*u*) is an intercept dependent upon *u*, and **β**(*u*) is a vector of regression coefficients of *v* dependent upon *u*.

In the current work, we considered a simplified two-stage DBN (TS-DBN) which uses a Markov assumption that the values of variables at time *t + 1* are independent of earlier time points (*t − 1* and earlier) given the variable values at time *t*. We further assumed that all transitions of interest were from time *t* to time *t + 1*, and not within time *t* or *t + 1*. Given the DBN, we use the expectation of variable *y* at time *t + 1*, as the predicted outcome for *y*_*t+1*_, which is simply:


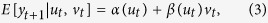


where ***u***_***t***_ is a vector of the assignments of discrete parents *u* at time *t*, and ***v***_***t***_ is a vector of assignments of continuous parents *v* at time *t*. We modified the CGBayesNets package to compute this expectation in simple TS-DBNs.

Given the DBN with distribution parameters θ, the predicted value for discrete variable *x* at time *t+1* is given by the following, where *z* is a value of discrete variable *x, **u***_***t***_ is an assignment to the (necessarily discrete) parents *u* of *x* at time *t*, Γ() is the Gamma function, and the notation *α*[***u***_***t***_*,z*] refers to the cardinality of the prior assumed sample size having variables *u* = ***u***_***t***_ and *x* = *z*, similarly *n*[***u***_***t***_*,z*] refers to the number of data points in *D* such that *u* = ***u***_***t***_ and *x* = *z*.


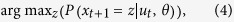



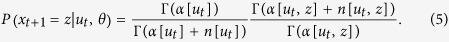


This equation is a simplification of Sebastiani *et al.*^3^, and its value can be directly obtained from Bayesian posterior likelihood computations already included in CGBayesNets.

Typical BN models calculate the posterior probability of the data given the network to determine the network that best fits the data^28^. We use this posterior probability to provide concrete expectations of the data and determine unlikely abruptions. We further adapted the software in CGBayesNets^27^ to explicitly output


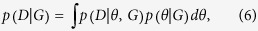


that is, the posterior probability of the data, *D*, given the DBN graph *G*, and DBN parameters θ. We rely on the CGBayesNets package to compute this according to standard Bayesian equations, further details of which are available in McGeachie, Chang, and Weiss^28^.

Our modifications that enable these DBN computations are available at www.cgbayesnets.com, where they are integrated into the latest release of the CGBayesNets package.

## Results

### Dynamic Bayesian Network

We constructed a DBN model of the infant gut microbial ecosystem (see Fig. 1). This model encodes temporal conditional statistical dependence in the following way: each node is independent of all other node’s current time-point values, each node is dependent upon its immediate upstream neighbors’ values at the previous time point, and given those values, is independent of all other nodes’ previous values.

This model included the most commonly observed microbiota classes: the top three being *Bacilli, Clostridia*, and *Gammaproteobacteria*. The model also included *Actinobacteria, Betaproteobacteria, Bacteroidia, Holophagae, Fusobacteria, Flavobacteria, Epsilonproteobacteria*, and the unclassified category of bacteria, all of which were absent in most samples and, when present, were detected at small concentrations. The model also included clinical variables, primarily several different measures of time: days of antibiotics, day of life of sample obtained, gestational age at birth, and post-conceptional age when sample obtained; and also including the binary indicator for the infant being cared for in an open (double) or closed (single) room. These measures of time were primarily indicators of *Clostridia* level, which increases with post-conceptional age. This is a result previously observed in this dataset by La Rosa *et al.* and identified here with the DBN. Rebuilding the model without *Holophagae* and *Cyanobacteria*, two possible environmental contaminants, did not alter results (data not shown).

**Figure 2:**
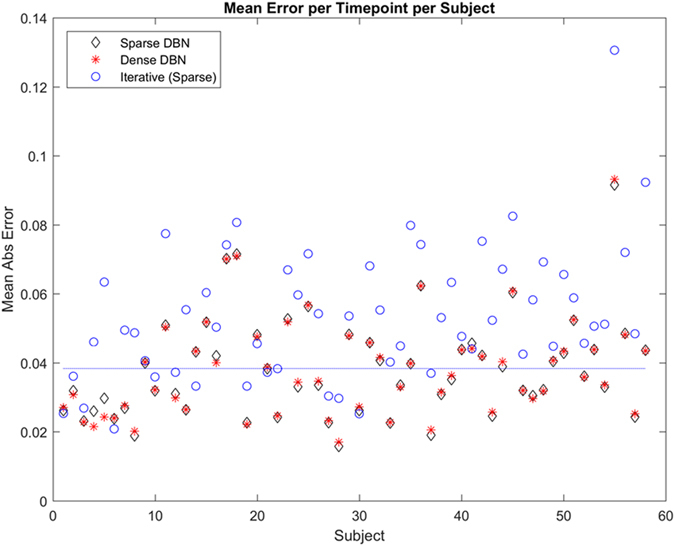
Comparative accuracy of DBN models. Accuracy in mean absolute error per subject is shown, where error is averaged across all bacteria taxa included in the Sparse network (Fig. 1). The Sparse DBN is simpler and performs better on subjects that are easier to predict (below empirical average of sparse networks’ mean absolute error, dashed blue line), while the Dense DBN (Supplemental Fig. 1) contains many more connections and performs better on subjects that are harder to predict (above the dashed blue line). This effect is significant when the two outliers (subjects #4 and #5) are removed (p = 0.045, t-test). Iterative prediction with the sparse network using only the first sample for each subject (blue circles) resulted in much larger average error per subject (p < 0.001, t-test).

Average accuracy of the DBN in predicting each subject (n = 58) is shown in Fig. 2. Accuracy was measured in mean absolute error per sample, using the previous sample to predict the subsequent sample. Accuracy was averaged per subject across multiple samples. Some subjects were easier to predict than others. This is a general characteristic of this cohort: some of the subjects display unusual or drastic changes of microbiome sample composition from one time point to the next; La Rosa *et al.* referred to these events as *abruptions*. Overall, two infants showed statistically significant abruption events (see Abruption Events, below). We further found that samples were easier to predict when the time between that sample and the previous one was smaller (Supplemental Fig. 3, p = 0.048, two-tailed t-test). Details about specific abruption events may be found in the discussion.

**Figure 3:**
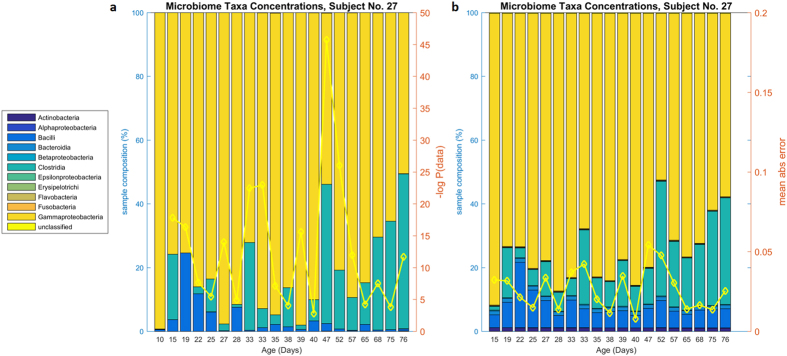
Actual and predicted microbiome composition for Subject No 27. Subject No 27, panel (**a**) microbiota taxa abundances by sample. Panel (**b**) predicted microbiota taxa abundances by DBN. This subject shows a somewhat typical distribution of microbiota, dominated by *Gammaproteobacteria* that gives way to *Clostridia*. Small amounts of *Bacilli* are present, along with trace amounts of *Actinobacteria*. On the second axis, panel (**a**) shows the posterior likelihood of the data conditioned on the previous sample (yellow line); shown in negative log scale. Panel (**b**) shows predictions of bacteria taxa abundances given the previous sample’s abundances, according to the DBN. The prediction for a sample is based on the data from the previous sample. The second axis shows the mean absolute error per sample (yellow line).

We further investigated the accuracy of DBN microbiota taxa concentration predictions by examining individual subjects. One subject (number 27) is shown in Fig. 3. All 58 subjects are similarly shown in an Appendix. This subject is representative of many of the subjects in the cohort because *Gammaproteobacteria, Clostridia*, and *Bacilli* dominate; in general these three make up the majority of the microbial ecology of the infants studied. In the DBN, these three bacteria are connected; with prior levels of *Clostridia* influencing levels of *Bacilli*, and prior levels of *Bacilli* influencing levels of *Gammaproteobacteria*; in addition age (post-conceptional age when sample obtained) influences *Clostridia*. Our confidence in the dynamic relationship between two variables (nodes) is captured in the natural log Bayes Factor (Supplementary Table S1). Two of these relationships are stronger than most inter-bacterial relationships (natural log Bayes Factor^29^ 7.9 for age->*Clostridia*, 0.3 for *Clostridia*->*Bacilli*, and 2.5 for *Bacilli*->*Gammaproteobacteria*; median log Bayes Factor is 0.65). Infants where these bacteria dominate are generally well-predicted. In general, the DBN predicts increasing amounts of *Clostridia* over time, residual amounts of *Bacilli*, and increasing amounts of *Gammaproteobacteria* that then give way to *Clostridia*. These trends are visible in Fig. 3 by comparing the true data (Fig. 3, panel a) to the predicted data (panel b). This subject also displays relatively minor changes from one time point to the next, resulting in both low prediction error and reasonable posterior probability of the true data.

We also observed connections between the three dominant bacterial classes and other relatively rare taxonomic groupings. For instance, abundances of *Bacilli* were dependent upon prior *Actinobacteria* levels. *Clostridia* abundance depended upon prior abundance of *Fusobacterium. Actinobacteria* predicted abundance of *Bacteroidia*.

### Abruption Events

**Figure 4:**
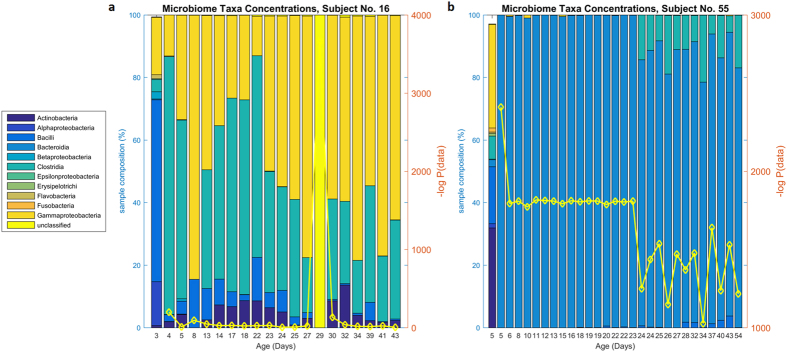
Subjects with unlikely abruptions. Subject 16 progresses through a typical volatile mix of *Bacilli, Clostridia*, and *Gammaproteobacteria*, but at day 29 this is replaced by 100% unclassified bacteria. Subject 55 has an unusual mix of rare bacteria taxa initially, but this is entirely replaced by *Bacteroidia* subsequently. On the second axis, the yellow line shows the posterior likelihood of the data conditioned on the previous sample, in negative log scale.

Abruption events, or large and unlikely departures from the previous sample’s abundances of bacteria, were visually evident in several of the subjects. We quantified the likelihood of these abruptions by calculating the posterior probability of the data from the network; this gives a quantitative estimate of the probability of seeing a more extreme combination of bacterial taxa, where this probability is based on the posterior density functions for each bacteria taxa (see Methods). We were unable to predict these abruption events: the DBN has learned an essentially conservative model that predicts changes primarily between *Clostridia, Bacilli*, and *Gammaproteobacteria*. We did quantify the likelihood of these abruptions as follows: we reported the posterior probability of a data sample based on the DBN model and the previous data sample for that subject. This resulted in probability estimates (*e.g.*, Fig. 3) reported in negative log probabilities. We identified statistically significant outliers from the distribution of posterior sample likelihoods (below (0.05/58) on the z-scale of likelihoods, see Methods), identifying subjects experiencing at least one anomalous posterior data sample likelihood, and correspondingly, an unlikely abruption. This resulted in two subjects (numbers 16, log(p) = −3661, and 55, log(p) = *−*2413) experiencing statistically significant abruptions (Fig. 4). These two subjects may represent natural anomalies, or in these particular cases, it may represent some type of processing error. We accordingly removed these two subjects, and repeated our measure of abruptions, obtaining three subjects that experience either unusually high amounts of *Bacteroidia* (number 17), or statistically unlikely transitions to majority-*Bacteroidia* samples (numbers 52 and 53), shown in Supplemental Fig. 4. The ability of our method to identify these events, which may represent some type of anomaly, whether in the sample processing or in the actual microbiome, is a strength of our DBN approach.

### Iterative Prediction

**Figure 5:**
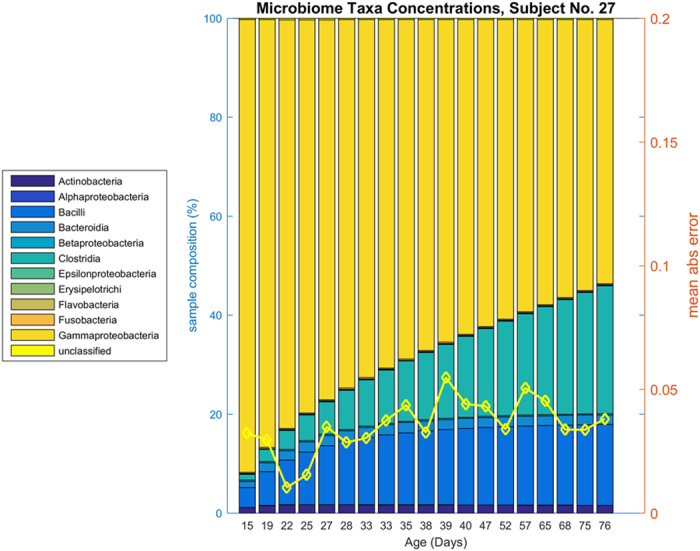
Iterative Prediction of subject number 27. Compare Fig. 3, Panel (**a**). This presents an idealized prediction of the trajectory of subject number 27 from day 15 iteratively simulated into the future. The second axis shows the mean absolute error per sample (yellow line).

To investigate the DBN model’s ability to predict final outcomes from the initial conditions, we performed iterative prediction. In this scenario, we used the results from our prediction of one time point as input into the prediction of the next time point, and thus iteratively predicted a microbiome trajectory only from initial conditions. Average predictive accuracy in mean absolute error per subject was much higher than prediction using every sample (p < 0.001, two-tailed t-test) and is shown on Fig. 2. In some cases, the iterative prediction provides what appears to be a smoothed or idealized progression compared to the volatility in the measurements of the actual daily bacteria concentrations for a given subject (example, Fig. 5, subject number 27. Compare Fig. 3). In some subjects, the iterative prediction method had lower mean absolute error than the every-sample method; inspection showed these were either subjects with only 2–5 samples total, or subjects where one outlier sample biased prediction by the every-sample method (e.g., subject #14, Supplemental Fig. 2), however this may also be due to chance.

**Figure 6:**
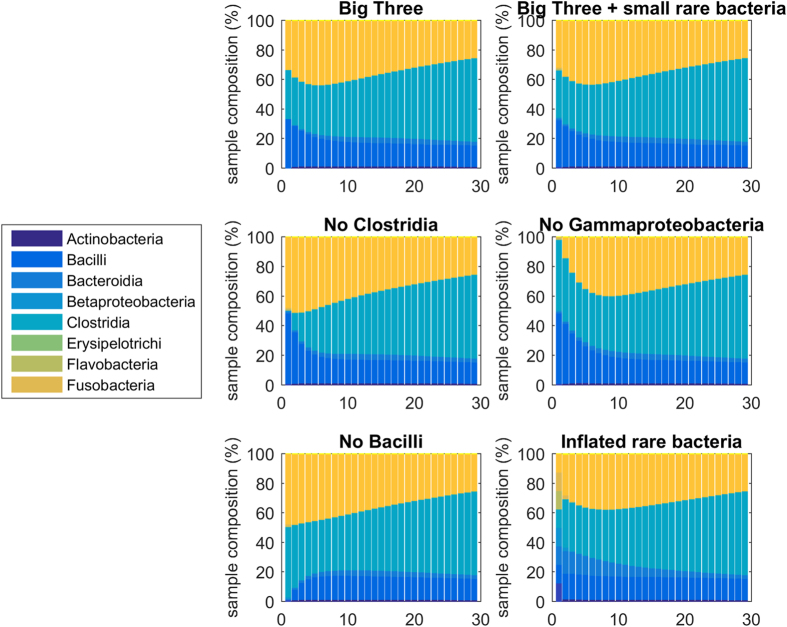
Iterative predictions of infant gut microbiome composition with different initial conditions. From left to right and top to bottom, initial conditions are simulated starting with an even amount of the three predominate bacteria (*Clostridia, Bacilli*, and *Gammaproteobacteria*); those are then simulated with small amounts of rarer bacteria; next initial conditions with no *Clostridia* are simulated; then no *Gammaproteobacteria*; then no *Bacilli*; and finally initial conditions with very large amounts of rare bacteria. In all cases the differences in initial conditions are largely gone by 20 time steps.

Our iterative predictions displayed a remarkable insensitivity to initial conditions. This is demonstrated by our analysis using synthetic initial conditions of six varieties: 1) initial conditions composed of an even split between the three main bacteria taxa – *Bacilli, Clostridia*, and *Gammaproteobacteria*; 2) the same even split with small amounts of other rare taxa included; 3) similar to condition 2, but without any *Clostridia*; 4) similar to condition 2, but without any *Gammaproteobacteria*; 5) similar to condition 2, but without any *Bacilli*; and 6) initial conditions composed of an even split between each rare type of bacteria (Fig. 6). In each case, after 15 or 20 time points, the populations converge to the same pattern: increasing *Clostridia*, decreasing *Gammaproteobacteria*, residual *Bacilli*, and any rare bacteria taxa have decreased to very minor amounts. This is the general behavior of the DBN model; and it captures well the insight from the original La Rosa *et al.* paper^13^.

### Sparse Vs. Dense Networks

For comparison purposes, we also generated a DBN with many more edges and including more of the rare bacteria (see Methods). This resulted in a denser network (Supplemental Fig. 1). We hypothesized that this would be able to predict microbiome transitions involving rare bacterial taxa to a greater extent than our original sparser network model. In general, this model displayed the same characteristics as the sparse network model: capturing the essential relationship between *Bacilli, Clostridia*, and *Gammaproteobacteria*; while displaying little difference in the predictions involving rare bacteria concentrations. Although per-sample prediction accuracy differed little between the sparse and dense DBN models (Fig. 2), we did notice that the sparse network was better at predicting subjects which had lower total prediction error (below the empirical mean of the mean absolute error of the sparse model, blue line on Fig. 2); while the denser network was better at predicting subjects with greater total error (above the blue line on Fig. 2). We found this trend was statistically significant only after removing the two outliers (subjects number 4 and 5), at p = 0.045 (two-tailed t-test).

## Discussion

Although the developing infant gut microbiome remains understudied, La Rosa and colleagues collected and analyzed extensive data on its progression in early life, in a cohort of preterm infants admitted to a NICU^13^. With 922 microbiome assessments, this is an attractive cohort for DBN analysis, of which other authors have suggested that 1000 samples is a good baseline for reconstructing an adequately accurate DBN^25^ La Rosa *et al.* used this data to demonstrate the succession of bacterial colonization (from *Bacilli* to *Gammaproteobacteria* to *Clostridia*), and showed the importance of post-conceptional age to the developing infant gut microbiome. They further concluded that (with rare exception) the trajectory of the infant gut microbiome was independent of the initial conditions. Our analysis of this data set, using DBNs, confirmed many of La Rosa *et al.*’s findings, with some important additions. With a DBN model, we were able to: (1) identify how the three dominant bacterial classes influence one another over time; (2) account for the role of relatively rare taxa in influencing these dominant groups; (3) determine the importance of initial conditions (microbiome profile immediately after birth) for iterative prediction of the post-natal microbiome trajectory; and (4) identify samples that depart from the expected trajectory of infant gut microbiome development in the first month of life.

Given the choreographed sequence of progression consistently observed in La Rosa *et al.*^13^ (initial predominance of *Bacilli*, followed by *Gammaproteobacteria*, then *Clostridia*), it may appear as though *Bacilli* are reduced as the result of increasing *Gammaproteobacteria*, and that subsequent decreases in *Gammaproteobacteria* are the direct result of increasing *Clostridia*. DBN analysis of these data show that *Gammaproteobacteria* is not necessarily an intermediary in the progression from predominance of *Bacilli* to *Clostridia* (*Bacilli* concentrations are independent of *Gammaproteobacteria* levels, but do depend on infiltrating *Clostridia*). Although the true nature of ecological change in infant gut microbiome after birth is difficult to know with certainty, our model may yield some insight into the dynamics of the well-known shift^8^,^9^,^10^ from micro-organisms that tolerate or utilize oxygen (facultative anaerobes) to populations of obligate anaerobes. Results from the DBN model suggest that as initial facultative anaerobe colonizers (*Bacilli*) are diminished by increasing abundance of obligate anaerobes (*Clostridia*), a second class of facultative anaerobes (*Gamma-proteobacteria*) momentarily out-compete the *Bacilli* and flourish temporarily. After this temporary increase, *Gamma-proteobacteria* ultimately gives way to obligate anaerobes (*Clostrida*). While *Clostridia* is a major predictor of decreasing *Bacilli* abundance over time, the DBN model also highlights *Actinobacteria* (many of which are obligate anaerobes), as having an equally important influence on declining *Bacilli* abundances. *Actinobacteria* are considered a rare taxon in this study, as they were present in relatively few infants. As was shown in La Rosa *et al.*^13^, post-conceptional age emerges in the DBN as the main factor associated with increasing abundance of *Clostridia*, which eventually predominates in the infant gut. In addition to post-conceptional age, the DBN model also demonstrates that prior levels of *Fusobacteria* (a relatively rare taxon) may influence *Clostridia* levels over time. Bayes Factors associated with the dynamic network connections between Clostridia, Bacilli and Gammaproteobacteria were highest, suggesting that these connections are more likely to represent true causal associations. While the DBN model allowed for the inclusion of relatively rare taxa, network connections involving these microbes (Actinobacteria, Fusobacteria and Betaproteobacteria) had lower Bayes Factors, indicating a lower level of confidence in these relationships (Supplementary Table S1). In general, infant characteristics, including antibiotics administered and single vs. double rooms, had little influence on the progression of the gut microbiota.

Most studies on development of the gut microbiome within the first months of life do not account for interactions between taxa, and instead consider changes in cluster profiles^30^,^31^ or employ time series analyses for community profiles as a whole^32^. Prior work on modeling microbe-microbe interactions within the infant gut microbiome over time has been conducted using targeted RNA microarrays, which limit investigation to a set of known bacteria^33^. In that work, nonlinear regression was used to identify significant interactions between gut microbiota at the phylum level^33^. Simulations using this model revealed a predominance of *Proteobacteria* at age four months, with *Firmicutes* occurring at mid-level abundance, and *Bacteroides* at lower levels (although concentrations may have been underestimated). *Proteobacteria, Firmicutes* and *Bacteroides* interacted with one another, showing intra-phylum competition (including a competitive relationship between *Bacteroides* and *Firmicutes*). While our model of La Rosa *et al.*’s data featured *Bacteroides* as a node within the network, *Bacteroides* abundance did not appear to influence levels of other taxa in the time window between birth and one month of age. Although direct comparisons between our class-level taxonomic model and Trosvik *et al.*’s phylum-level model are difficult to make, we did also capture interaction between *Firmicutes* (*Bacilli*) and *Proteobacteria* (*Gammaproteobacteria*). Our model of class-level taxonomic data also demonstrated interaction between two taxonomic classes within *Firmicutes* (the influence of developing *Clostrida* abundance on *Bacilli*).

While we focused on a sparse DBN model in this work, we also constructed a dense DBN that included a greater number of nodes (between rare taxa) and edges. The majority of network connections in the sparse DBN were reiterated in the dense model, but new connections between dominant groupings and rare taxa also appeared. For instance, in the dense model, prior levels of both *Flavobacteria* and *Bacilli* relate to current *Gammaproteobacteria* composition (in the sparse network *Flavobacteria* is not included as a node). Prior *Alphaproteobacteria* composition (another rare taxa left out of the sparse network) is featured as a predictor of *Bacilli* and *Clostridia* levels over time. Microbiome prediction accuracy for subjects with more complex microbiome ecology (due to the presence of various rare bacteria) improved when using the dense, as opposed to the sparse, DBN model.

For the majority of infants, the sparse DBN showed accurate predictions of the gut microbiome over time, based on prior composition from each previously measured time point. This type of model is ideal for data sets with multiple repeated assessments of the microbiome within a given time window. Alternatively, the iterative prediction model, a special case of the DBN method, makes sequential predictions over time using only one initial microbiome assessment. Using this model, the final prediction of the infant’s gut microbiome at about one month of age, based on assessment of the microbiome just days after birth, showed remarkable insensitivity to starting taxonomic composition. According to the DBN model, regardless of the initial conditions, *Clostridia* eventually predominate, with some remaining levels of *Bacilli* and *Gammaproteobacteria*. The lack of importance of initial conditions suggests extreme plasticity in colonization, and may indicate a larger role of host biology and/or external factors in directing colonization.

While, overall, the DBN showed accurate predictions of the microbiome, subjects with rapid, extreme changes in microbiome composition were difficult to predict. In this context, abruptions represent the degree to which a measured microbiome departs from a predicted microbiome given the underlying Dynamic Bayes Net model. Given the lack of knowledge on the development of the infant gut microbiome, it is difficult to know if the observed abruptions represent truly unlikely events (even including errors in sample collection or processing), or just normal, albeit sudden, changes that deviate from the typical course of microbiome progression. Furthermore, since the DBN model is learned from the data, the model has little ability to predict events that occur rarely within that data; and consequently assigns small probability to those events. Nevertheless, the DBN does provide an objective output of data likelihood and thus could be used to identify samples for further scrutiny. Accordingly, we may examine the two least likely abruptions shown in Fig. 4. The first seems the most likely to represent some sort of error in collection or data processing: the abruption replaces all data from day 19 in subject 16 with unclassifiable bacteria; the preceding and subsequent samples are both composed of standard mixes of *Actinobacteria, Bacilli, Clostridia*, and *Gammaproteobacteria*. While our general ignorance of the processes governing infant gut microbiome development makes rendering judgments of this type tenuous, we would propose that this sample be retreated or reexamined in some way. Perhaps stranger is the abruption in subject 55, in which (an unusual) variety of bacteria (present only in the initial measurement on day 5), is suddenly replaced by 100% *Bacteroidia*. However, in this case, all subsequent samples from this patient are also dominated by *Bacteroidia*. It seems possible that one, perhaps rare, outcome is for the infant gut microbiome to be replaced by *Bacteroidia*, which has been shown to be the major component of adult gut microbial populations^34^. Alternatively, the initial microbial composition may reflect measurement error or sample swapping; such rapid change (occurring on the same day (day 5)) is an extremely unusual event, and more sudden than compositional changes following even the strongest external perturbations (administration of broad spectrum antibiotics)^35^. If these two subjects are removed, the remaining abruptions detected are large quantities of Bacteroidia, which we consider to be much more likely to represent true biological transition and abruption. In general, little is known about the implications of abruptions in gut microbiome progression during early infancy; theoretically those abruptions that initiate a persistent change in gut microbiota (over days or weeks, such as the abruption show in subject 55), may have greater physiological impact on the host, as compared to highly transient abruptions.

While application of a DBN model to La Rosa *et al.*’s longitudinal microbiome data in preterm infants yielded new insights about microbiome prediction and interactions between microbes over time, the DBN also had some limitations. First, the observation that gender, mode of delivery, and breast feeding variables resulted in overfitting indicates that there was not enough data present in the dataset to accurately model their influence upon the microbiome with a DBN methodology. Second, the lack of data on rare taxa appeared to affect the prediction of those groupings. A third limitation is that while bacterial taxa may show statistical relationships within a network, the exact nature of the biological mechanisms (if any) underlying these relationships often remain unknown. Lastly, the results of the DBN in the population studied (preterm infants residing in the NICU), may not be generalizable to full-term infants who reside in a home environment shortly after birth; these preterm infants may be subject to unusual exposures in a hospital, may be at risk for conditions or microbial abnormalities due to prematurity, or even be unusually insulated from exposures in a controlled NICU setting.

More accurate prediction could be achieved with more detailed datasets, ideally with greatly increased frequency of microbiome observation, at least daily. It is possible that existing datasets could be used as priors for future datasets, thus increasing the accuracy of the predictions. The extent to which the daily microbiome concentrations in infants fluctuate is also a concern; with more data on the daily fluctuations, we may be able to estimate if a prediction is within the expected range of possible microbiomes for a particular infant on a particular day. Similarly, we don’t yet have a firm grasp of which environmental influences impact the microbiome concentration the most, and knowledge of those effects in future datasets would undoubtedly be helpful. Finally, our network methodology could be improved; perhaps modeling within time-slice interactions would lead to a greater understanding of the microbial interactions within the microbiome.

Application of a Dynamic Bayesian Network model to longitudinal gut microbiome data from pre-term infants admitted to a NICU identified important relationships between microbiome taxa, and was used to simulate future changes in microbiome composition from measured or synthetic initial conditions. Simulation studies revealed that initial composition of the gut microbiome mattered little for development of the infant gut microbiota at about one month of age. Likelihood estimates for sudden, dramatic departures from expected microbiome composition may be calculated using a DBN model, and may be useful for identifying laboratory error or true biological variation due to clinically relevant dysbiosis. Our analysis shows that DBNs can be applied to longitudinal microbiome data. Application of this method to longitudinal microbiome data from other cohorts of infants (i.e. non-diseased) may provide valuable insight into how the gut microbiome gets established.

## Additional Information

**How to cite this article**: McGeachie, M. J. *et al.* Longitudinal Prediction of the Infant Gut Microbiome with Dynamic Bayesian Networks. *Sci. Rep.* **6**, 20359; doi: 10.1038/srep20359 (2016).

## Supplementary Material

Supplementary Information
