# Supplementary material for: Longitudinal Prediction of the Infant Gut Microbiome with Dynamic Bayesian Networks
Source: Sci Rep. 2016 Feb 8;6:20359. doi: 10.1038/srep20359 (PMC4745046; doi:10.1038/srep20359)
Supplement: Supplementary Information [file srep20359-s1.pdf]

Longitudinal Prediction of the Infant Gut Microbiome with Dynamic Bayesian Networks

Michael J. McGeachie, PhD[1,*]

Joanne E. Sordillo, ScD[1,*]

Travis Gibson, PhD[1]

George M. Weinstock [2]

Yang-Yu Liu, PhD[1]

Diane R. Gold, MD, MPH[1]

Scott T. Weiss, MD[1]

Augusto Litonjua, MD[1]

[1] Channing Division of Network Medicine, Brigham and Women's Hospital, Boston, MA.

[2] The Jackson Laboratory for Genomic Medicine, Farmington, CT.

*Contributed equally to this work

Correspondence to:

Michael J. McGeachie, PhD.

Channing Division of Network Medicine

181 Longwood Ave

Boston MA, 02115

michael.mcgeachie@channing.harvard.edu

617-525-2141

Supplemental Table 1.

Log BayesFactor	From	To
0.105	*Betaproteobacteria*	Day of life sample obtained
0.325	*Fusobacteria*	Day of life sample obtained
1.498	Gestational age at birth - weeks	postconceptional age when sample obtained
0.323	*Fusobacteria*	postconceptional age when sample obtained
7.593	Day of life sample obtained	days of antibiotics
2.031	*Betaproteobacteria*	days of antibiotics
9.87E-10	*Holophagae*	*Actinobacteria*
0.178	*Actinobacteria*	*Bacilli*
0.295	*Clostridia*	*Bacilli*
14.246	Openroom	*Bacteroidia*
0.92	*Actinobacteria*	*Bacteroidia*
7.87E-05	*Epsilonproteobacteria*	*Betaproteobacteria*
0.011	*Flavobacteria*	*Betaproteobacteria*
7.936	postconceptional age when sample obtained	*Clostridia*
0.652	*Fusobacteria*	*Clostridia*
2.509	*Bacilli*	*Gammaproteobacteria*
0.647	unclassified	*Gammaproteobacteria*

Supplemental Table 1. Log BayesFactors for connection strengths between bacteria in the Bayesian network of Figure 1. Positive numbers indicate significant statistical evidence of a connection between nodes, measured in ratio of change in posterior likelihood of a model with the connection to a model without the connection. This shows the strongest connection between bacteria taxa is from Bacilli to Gammaproteobacteria.

Supplemental Figure 1.

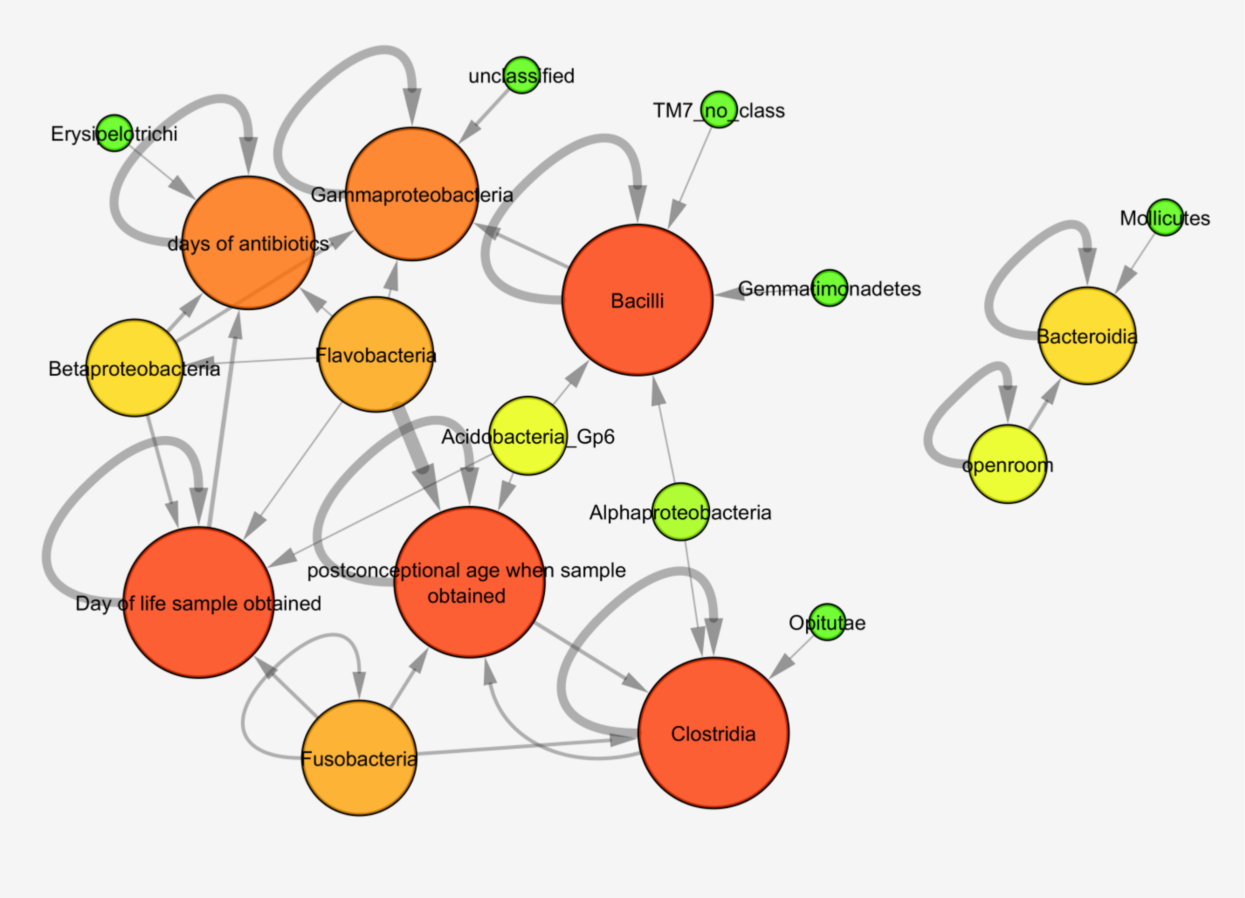

Supplemental Figure 1. A more dense DBN model of infant gut microbiome succession. The DBN network included the clinical and bacteria taxa variables pictured above. Nodes indicate variables, with size and color indicating greater node degree. Edges indicate temporal statistical influence: the source node's prior value predicts the target node's present value. Edge thickness indicates strength of statistical dependence.

Supplemental Figure 2.

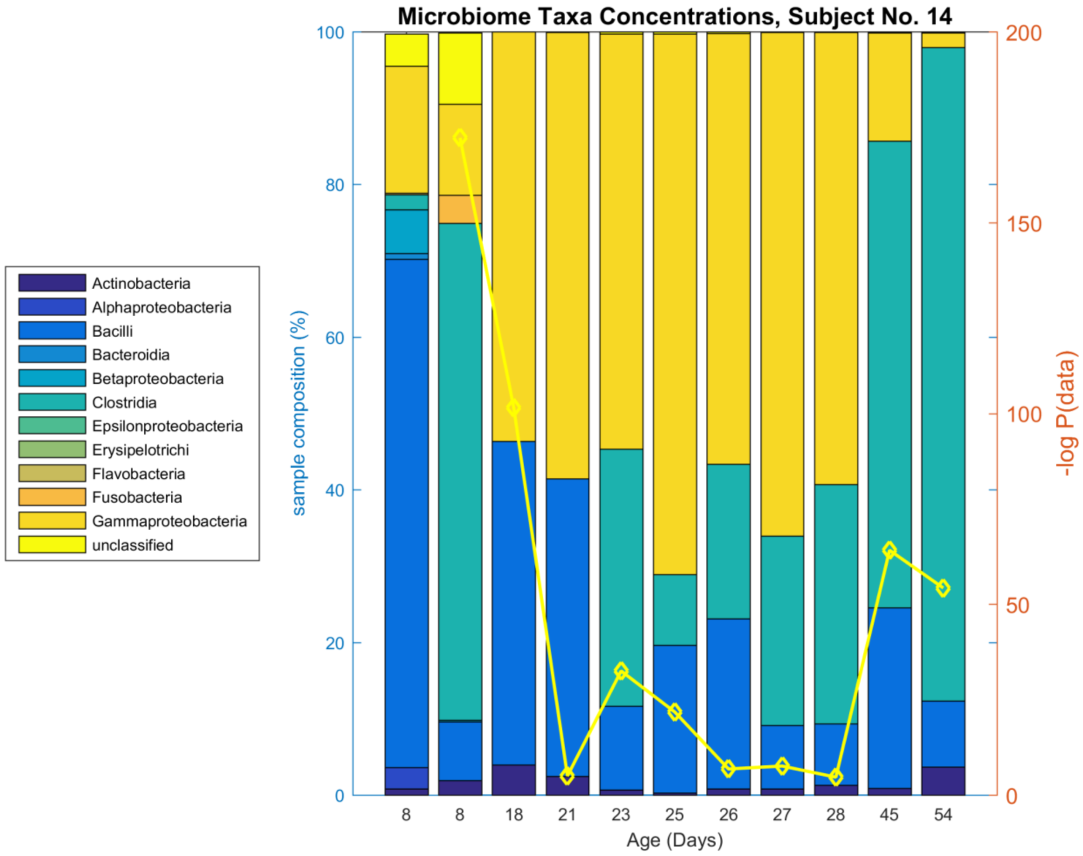

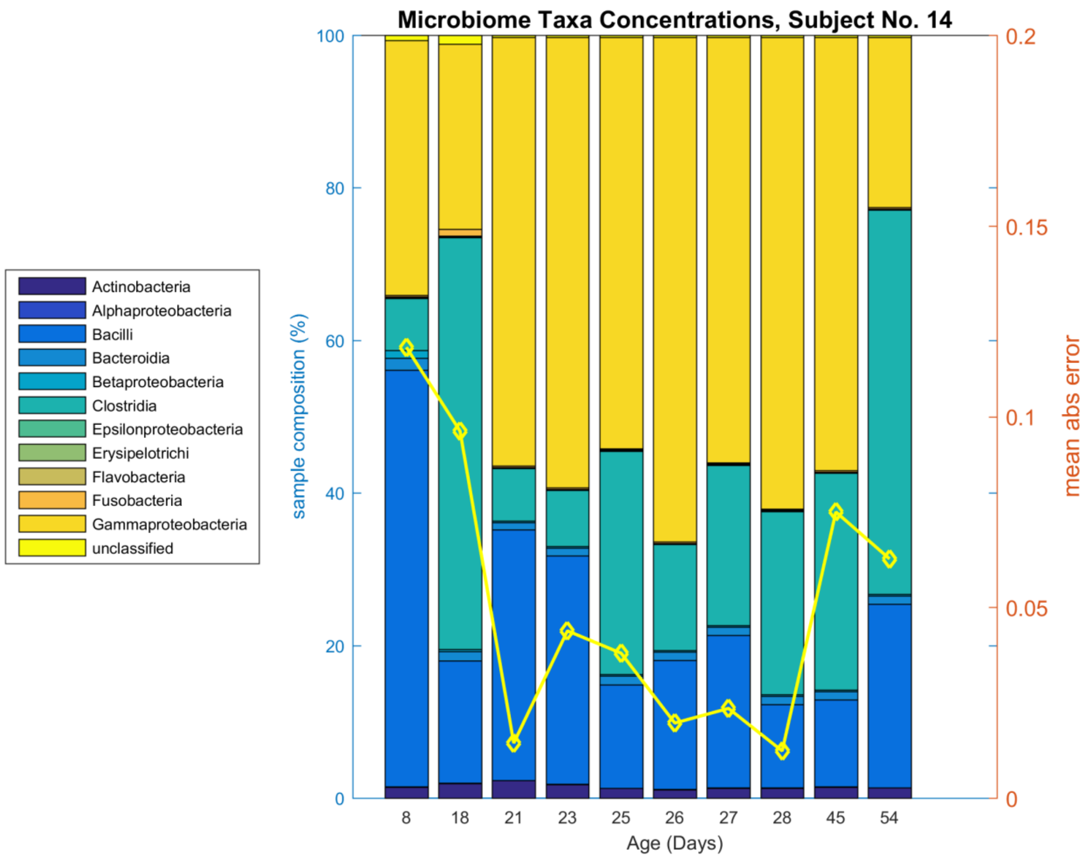
Microbiome Taxa Concentrations, Subject No. 14
Actinobacteria
Alphaproteobacteria
Bacilli
Bacteroidia
Betaproteobacteria
Clostridia
Epsilonproteobacteria
Erysipelotrichi
Flavobacteria
Fusobacteria
Gammaproteobacteria
unclassified
sample composition (%)
mean abs error
Age (Days)
0
20
40
60
80
100
0
0.05
0.1
0.15
0.2
8
18
21
23
25
26
27
28
45
54

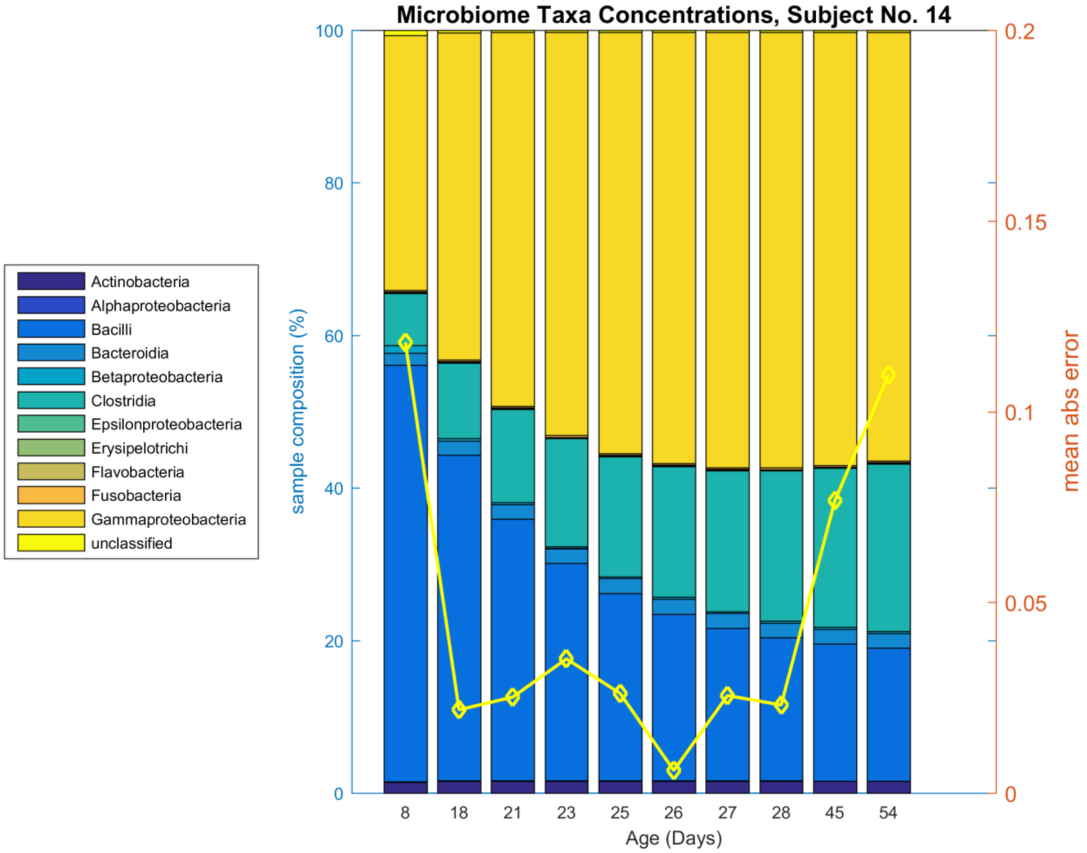

Supplemental Figure 2. Example of iterative prediction. Subject 14 was more accurately predicted by the DBN models using only the first sample of data and iteratively predicting; rather than predicting from each sample to the subsequent sample. In this case, this effect is due to the difficulty in predicting the sample for day 18.

Supplemental Figure 3.

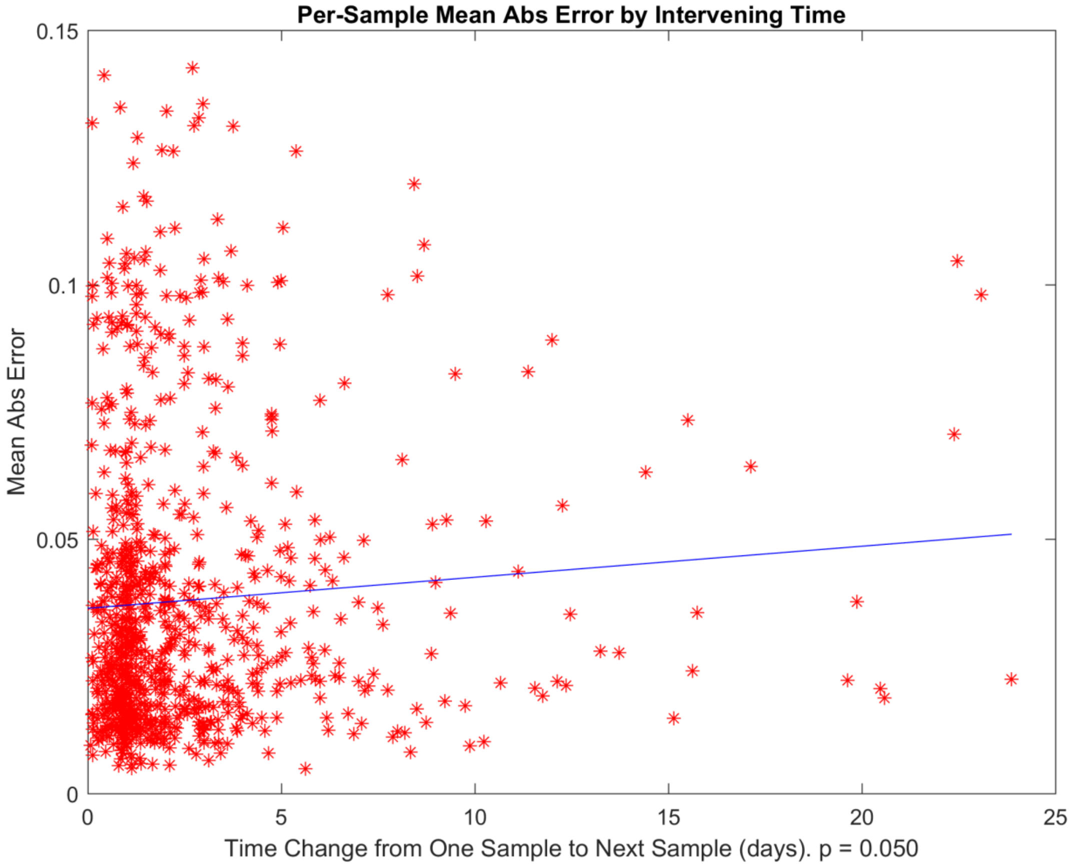

Supplemental Figure 3. Regression of prediction error on intervening time. Predicting the microbiome composition from one sample to the next is harder when the time between those samples increases. The blue regression line represents a significant trend with increasing error as intervening time increases (p = 0.048).

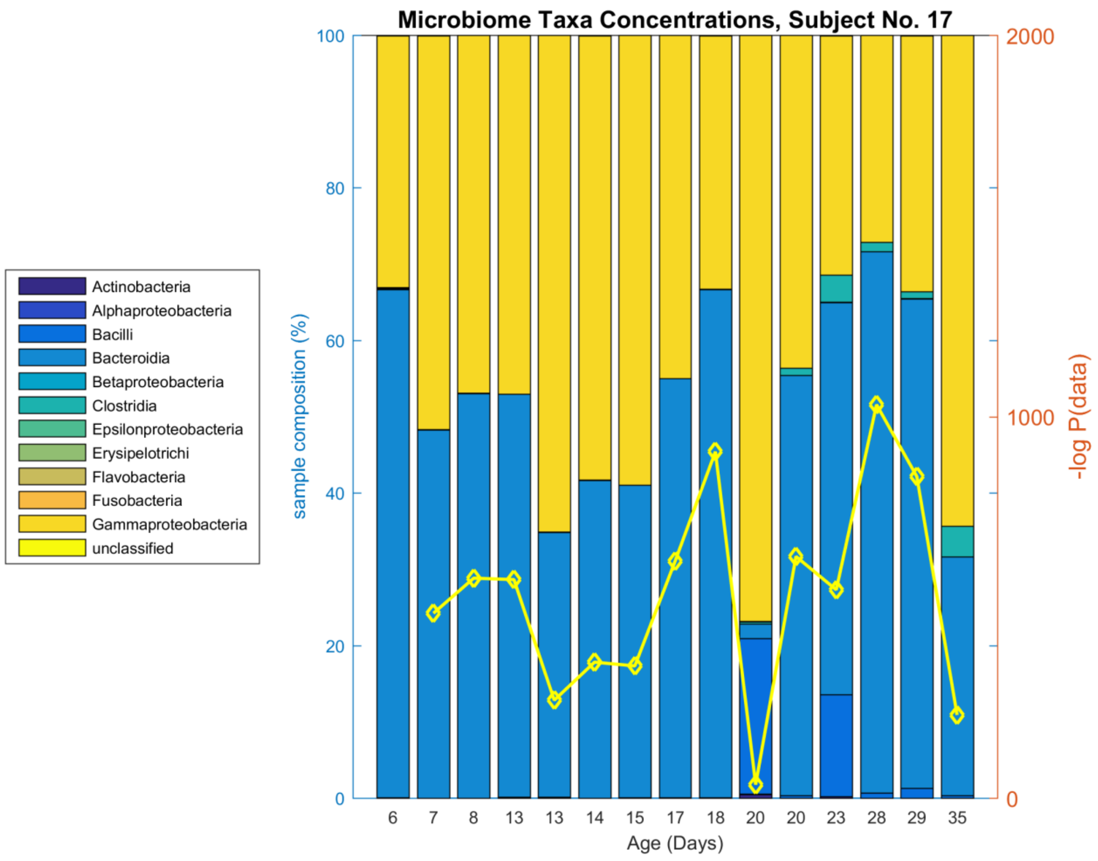
Microbiome Taxa Concentrations, Subject No. 17
Actinobacteria
Alphaproteobacteria
Bacilli
Bacteroidia
Betaproteobacteria
Clostridia
Epsilonproteobacteria
Erysipelotrichi
Flavobacteria
Fusobacteria
Gammaproteobacteria
unclassified
sample composition (%)
-log P(data)
Age (Days)
0
20
40
60
80
100
0
1000
2000
6 7 8 13 13 14 15 17 18 20 20 23 28 29 35

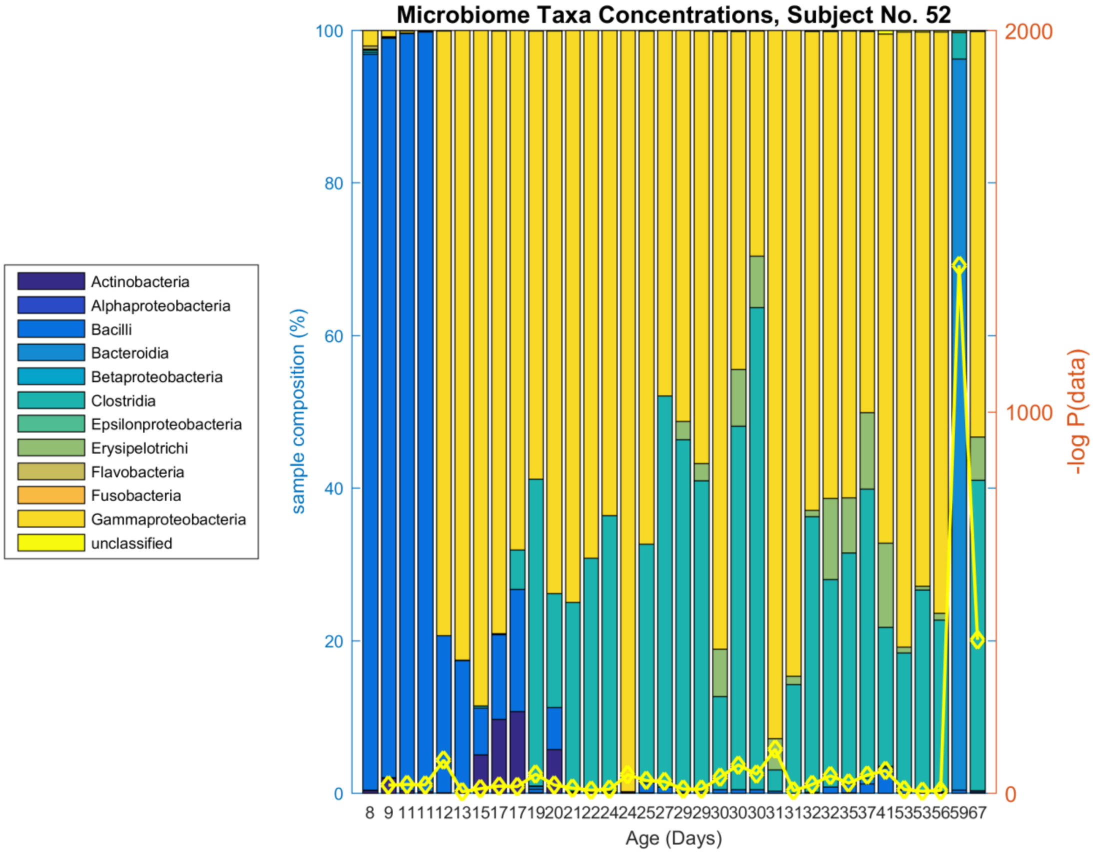

Microbiome Taxa Concentrations, Subject No. 52
Actinobacteria
Alphaproteobacteria
Bacilli
Bacteroidia
Betaproteobacteria
Clostridia
Epsilonproteobacteria
Erysipelotrichi
Flavobacteria
Fusobacteria
Gammaproteobacteria
unclassified
sample composition (%)
-log P(data)
Age (Days)
0
20
40
60
80
100
1000
2000
8 9 111112131517171920212224242527292930303031313232353741535356567

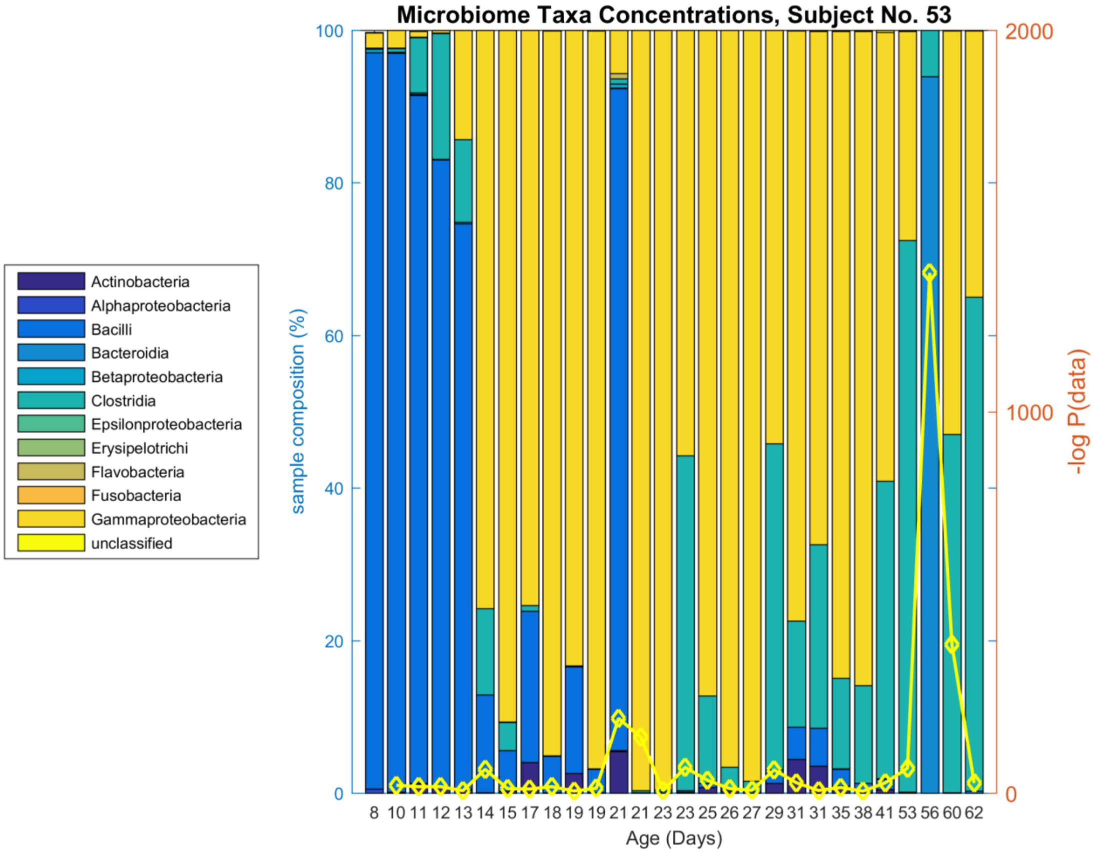

Supplemental Figure 4. Subjects experiencing statistically unlikely abruption events, after probable processing errors removed. Each subject experiences an unusual or unlikely amount of *Bacteroidia*.

Supplemental Appendix:

Microbiome data from each of 58 subjects, with likelihoods of data, and predicted microbiomes for that subject, with mean absolute error of prediction.

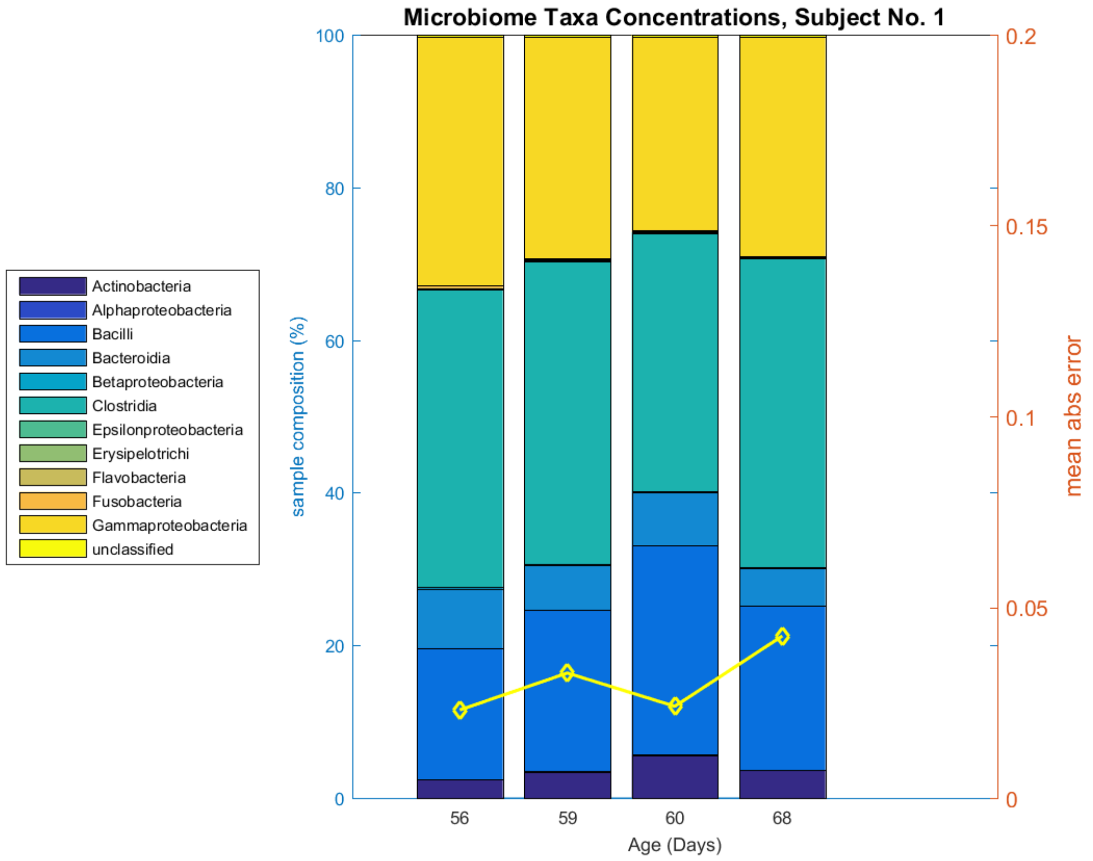
Microbiome Taxa Concentrations, Subject No. 1
Actinobacteria
Alphaproteobacteria
Bacilli
Bacteroidia
Betaproteobacteria
Clostridia
Epsilonproteobacteria
Erysipelotrichi
Flavobacteria
Fusobacteria
Gammaproteobacteria
unclassified
sample composition (%)
mean abs error
0
20
40
60
80
100
0
0.05
0.1
0.15
0.2
56
59
60
68
Age (Days)

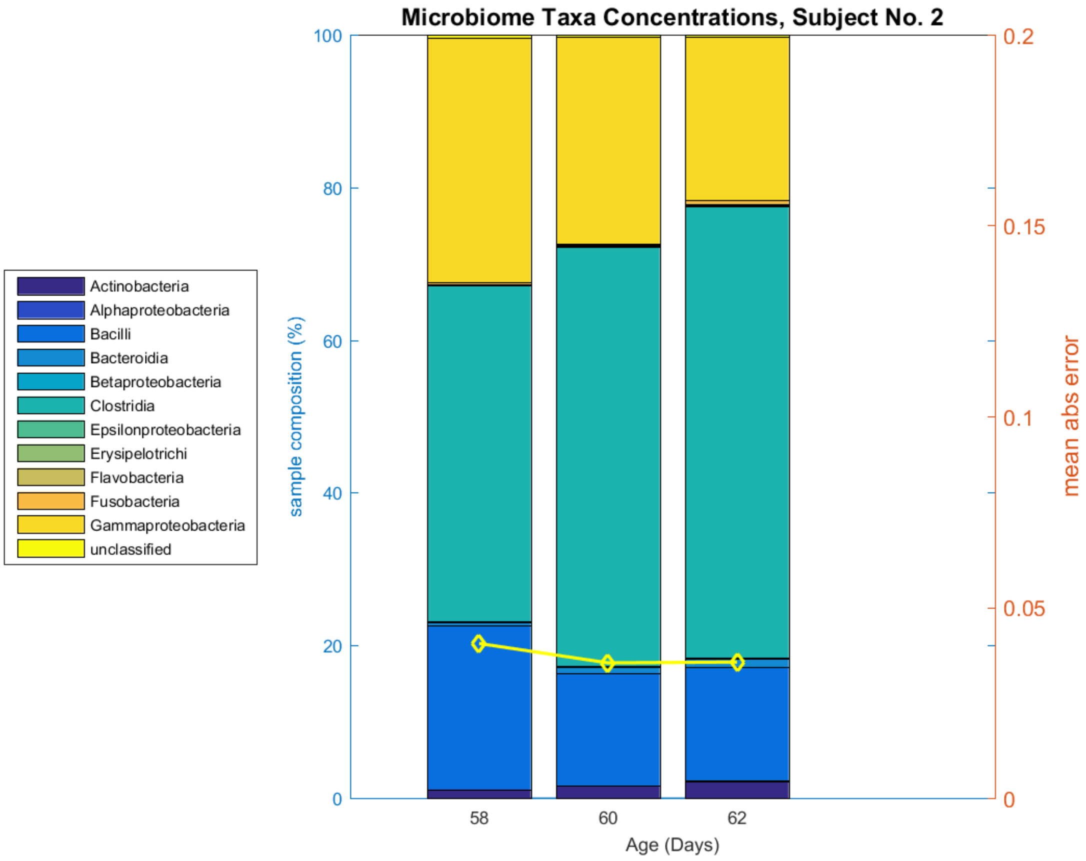

Microbiome Taxa Concentrations, Subject No. 2
Actinobacteria
Alphaproteobacteria
Bacilli
Bacteroidia
Betaproteobacteria
Clostridia
Epsilonproteobacteria
Erysipelotrichi
Flavobacteria
Fusobacteria
Gammaproteobacteria
unclassified
sample composition (%)
0
20
40
60
80
100
mean abs error
0
0.05
0.1
0.15
0.2
58
60
62
Age (Days)

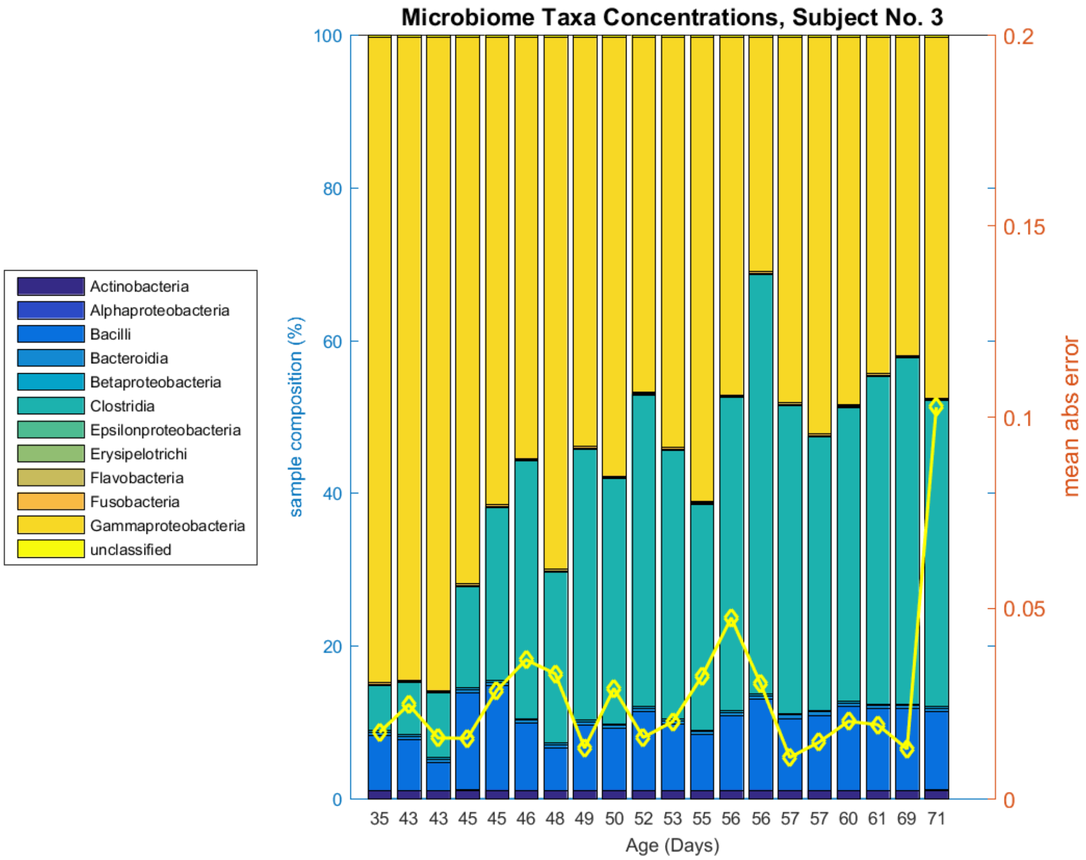
Microbiome Taxa Concentrations, Subject No. 3
Actinobacteria
Alphaproteobacteria
Bacilli
Bacteroidia
Betaproteobacteria
Clostridia
Epsilonproteobacteria
Erysipelotrichi
Flavobacteria
Fusobacteria
Gammaproteobacteria
unclassified
sample composition (%)
mean abs error
Age (Days)
35 43 43 45 45 46 48 49 50 52 53 55 56 56 57 57 60 61 69 71
0 20 40 60 80 100
0 0.05 0.1 0.15 0.2

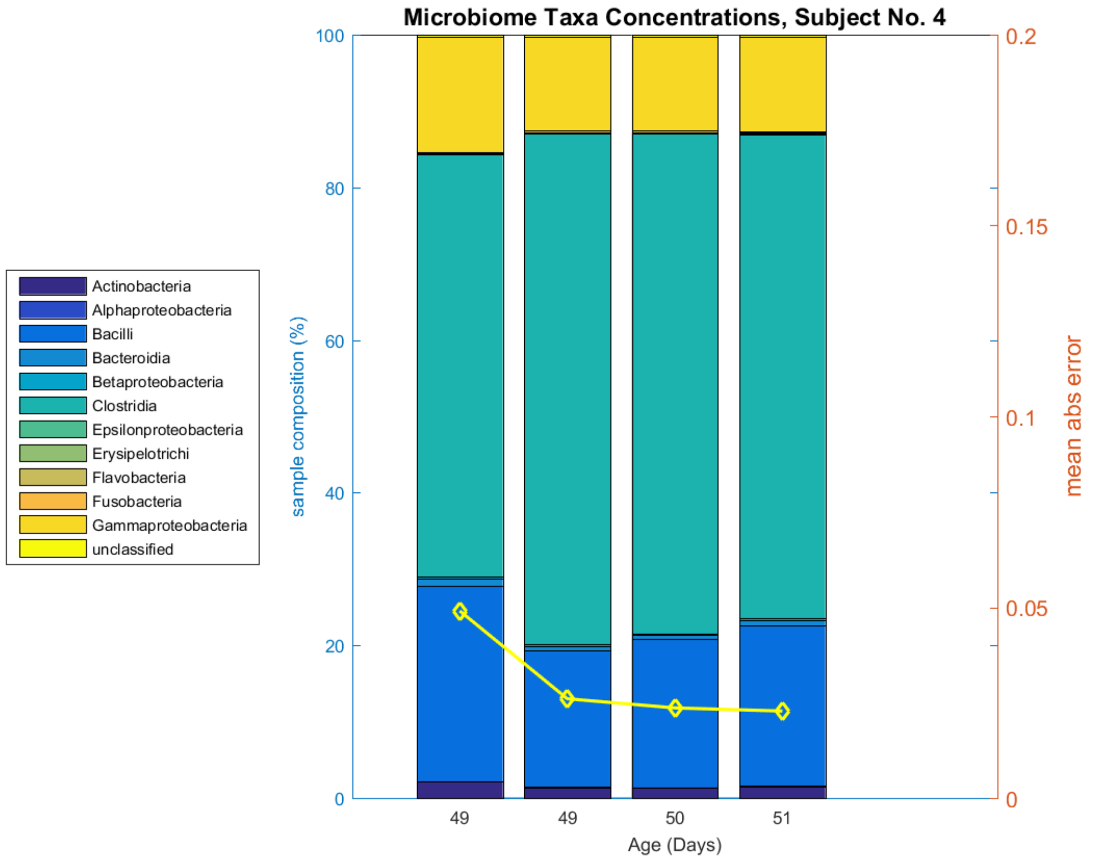

Microbiome Taxa Concentrations, Subject No. 4
Actinobacteria
Alphaproteobacteria
Bacilli
Bacteroidia
Betaproteobacteria
Clostridia
Epsilonproteobacteria
Erysipelotrichi
Flavobacteria
Fusobacteria
Gammaproteobacteria
unclassified
sample composition (%)
mean abs error
0
20
40
60
80
100
0
0.05
0.1
0.15
0.2
49
49
50
51
Age (Days)

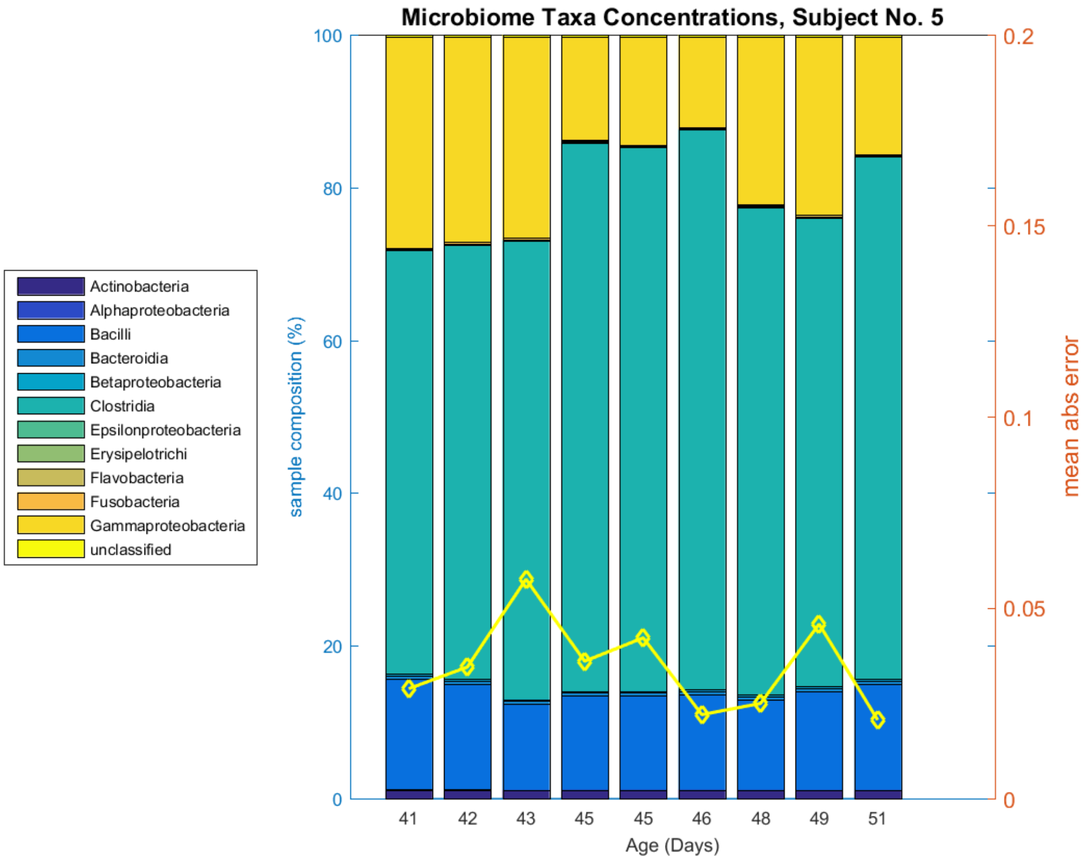
Microbiome Taxa Concentrations, Subject No. 5
Actinobacteria
Alphaproteobacteria
Bacilli
Bacteroidia
Betaproteobacteria
Clostridia
Epsilonproteobacteria
Erysipelotrichi
Flavobacteria
Fusobacteria
Gammaproteobacteria
unclassified
sample composition (%)
100
80
60
40
20
0
mean abs error
0.2
0.15
0.1
0.05
0
41
42
43
45
45
46
48
49
51
Age (Days)

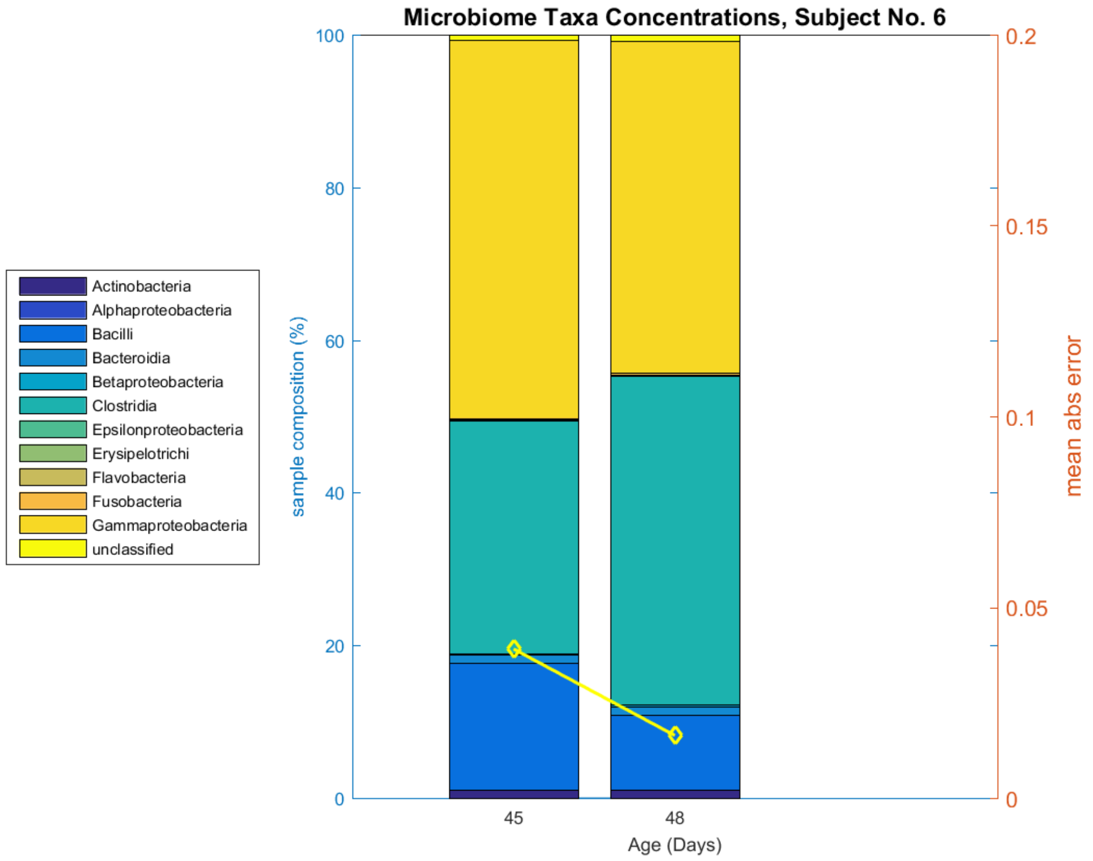
Microbiome Taxa Concentrations, Subject No. 6
Actinobacteria
Alphaproteobacteria
Bacilli
Bacteroidia
Betaproteobacteria
Clostridia
Epsilonproteobacteria
Erysipelotrichi
Flavobacteria
Fusobacteria
Gammaproteobacteria
unclassified
sample composition (%)
0
20
40
60
80
100
mean abs error
0
0.05
0.1
0.15
0.2
45
48
Age (Days)

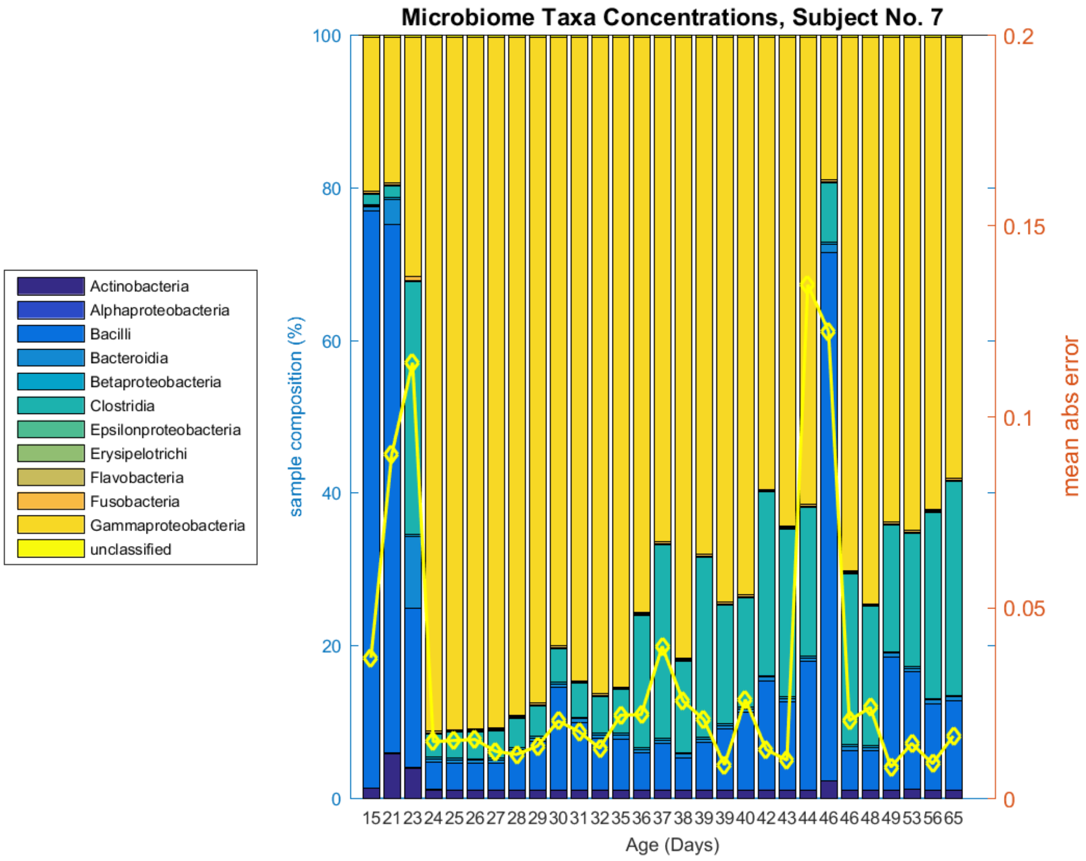
Microbiome Taxa Concentrations, Subject No. 7
Actinobacteria
Alphaproteobacteria
Bacilli
Bacteroidia
Betaproteobacteria
Clostridia
Epsilonproteobacteria
Erysipelotrichi
Flavobacteria
Fusobacteria
Gammaproteobacteria
unclassified
sample composition (%)
mean abs error
100
80
60
40
20
0
0.2
0.15
0.1
0.05
0
15 21 23 24 25 26 27 28 29 30 31 32 35 36 37 38 39 39 40 42 43 44 46 46 48 49 53 56 65
Age (Days)

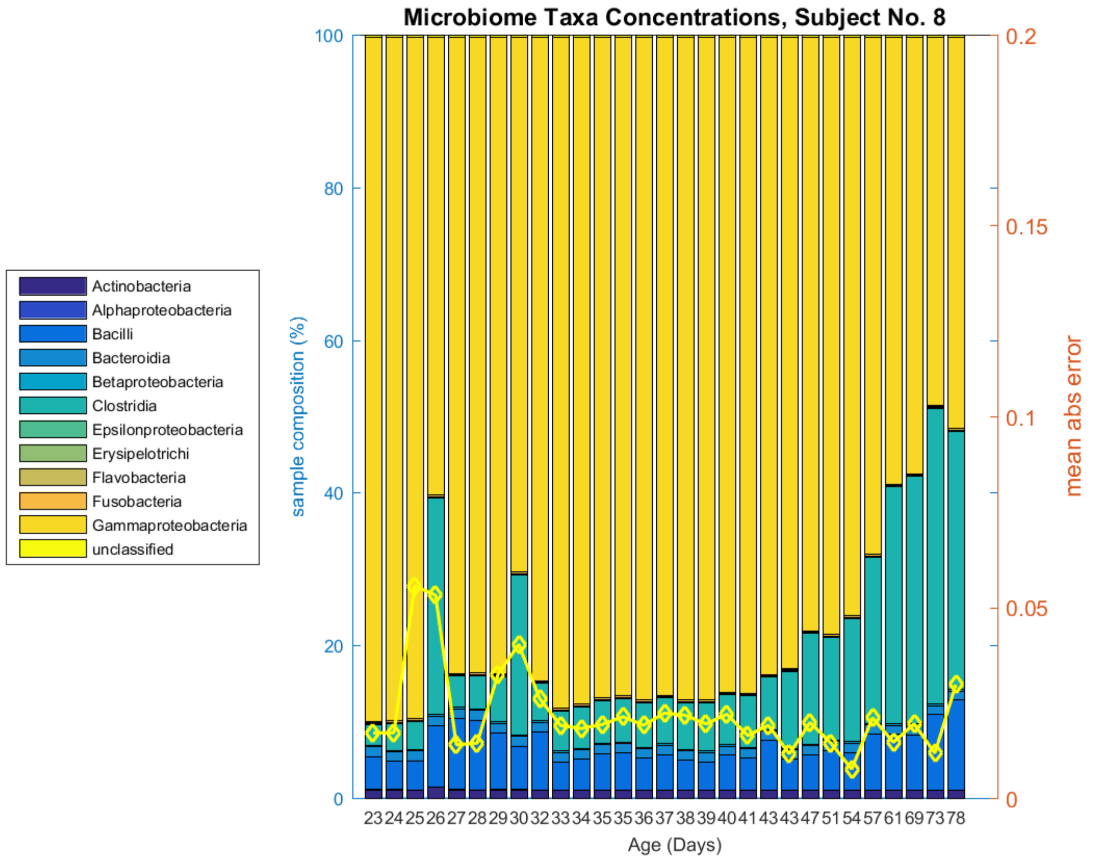
Microbiome Taxa Concentrations, Subject No. 8
Actinobacteria
Alphaproteobacteria
Bacilli
Bacteroidia
Betaproteobacteria
Clostridia
Epsilonproteobacteria
Erysipelotrichi
Flavobacteria
Fusobacteria
Gammaproteobacteria
unclassified
sample composition (%)
mean abs error
0
20
40
60
80
100
0
0.05
0.1
0.15
0.2
23 24 25 26 27 28 29 30 32 33 34 35 35 36 37 38 39 40 41 43 43 47 51 54 57 61 69 73 78
Age (Days)

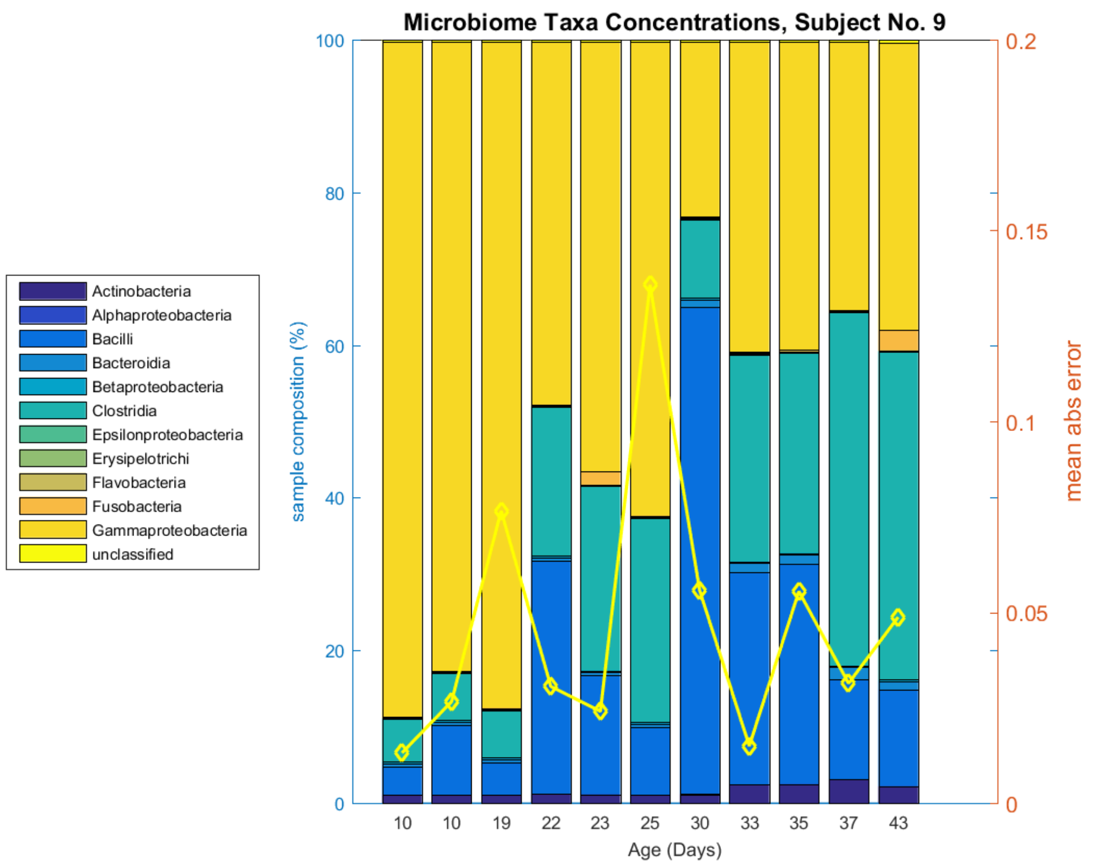
Microbiome Taxa Concentrations, Subject No. 9
Actinobacteria
Alphaproteobacteria
Bacilli
Bacteroidia
Betaproteobacteria
Clostridia
Epsilonproteobacteria
Erysipelotrichi
Flavobacteria
Fusobacteria
Gammaproteobacteria
unclassified
sample composition (%)
mean abs error
Age (Days)
0
20
40
60
80
100
0
0.05
0.1
0.15
0.2
10
10
19
22
23
25
30
33
35
37
43

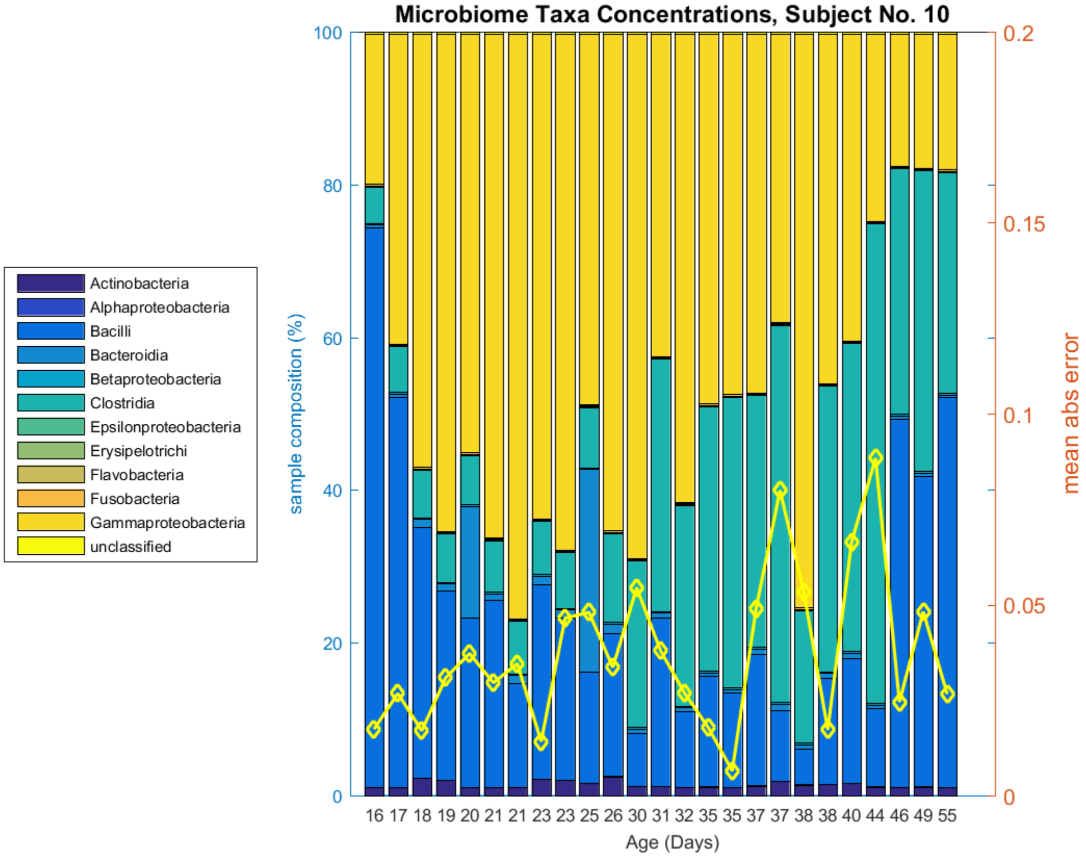
Microbiome Taxa Concentrations, Subject No. 10
Actinobacteria
Alphaproteobacteria
Bacilli
Bacteroidia
Betaproteobacteria
Clostridia
Epsilonproteobacteria
Erysipelotrichi
Flavobacteria
Fusobacteria
Gammaproteobacteria
unclassified
sample composition (%)
mean abs error
Age (Days)
16 17 18 19 20 21 21 23 23 25 26 30 31 32 35 35 37 37 38 38 40 44 46 49 55

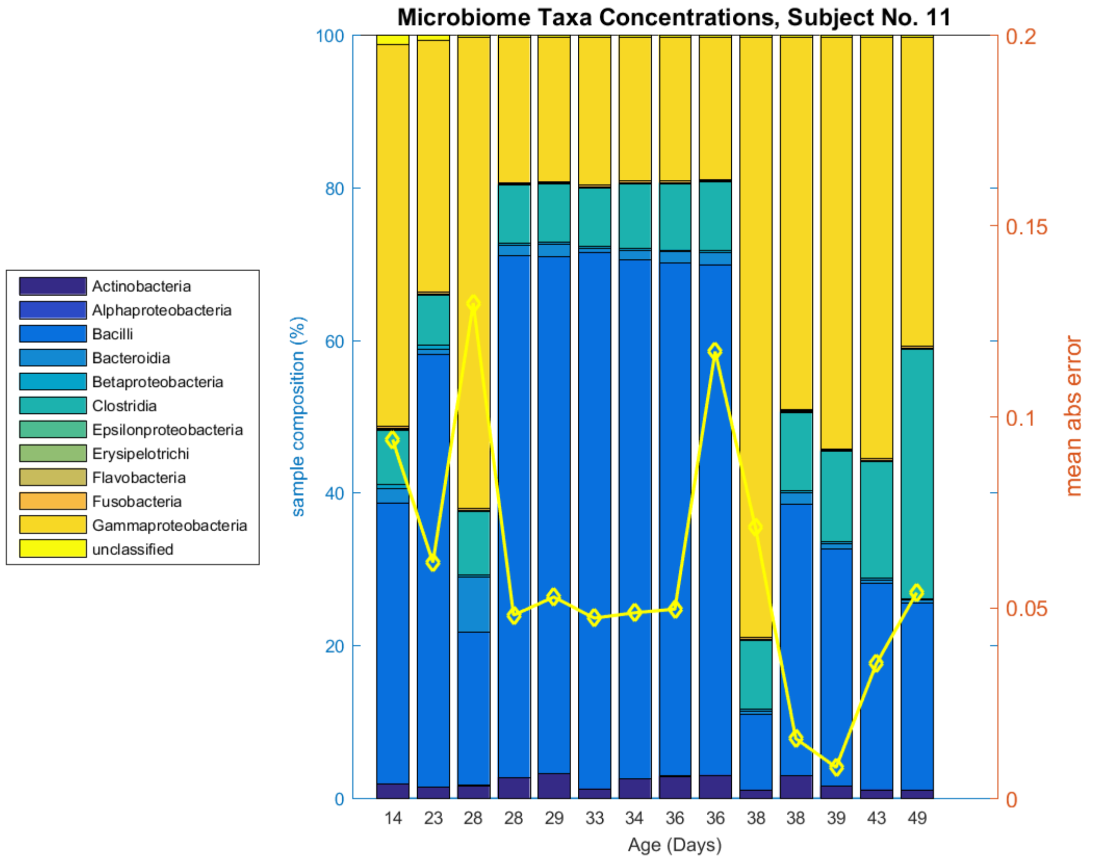
Microbiome Taxa Concentrations, Subject No. 11
Actinobacteria
Alphaproteobacteria
Bacilli
Bacteroidia
Betaproteobacteria
Clostridia
Epsilonproteobacteria
Erysipelotrichi
Flavobacteria
Fusobacteria
Gammaproteobacteria
unclassified
sample composition (%)
mean abs error
Age (Days)
14 23 28 28 29 33 34 36 36 38 38 39 43 49
0 20 40 60 80 100
0 0.05 0.1 0.15 0.2

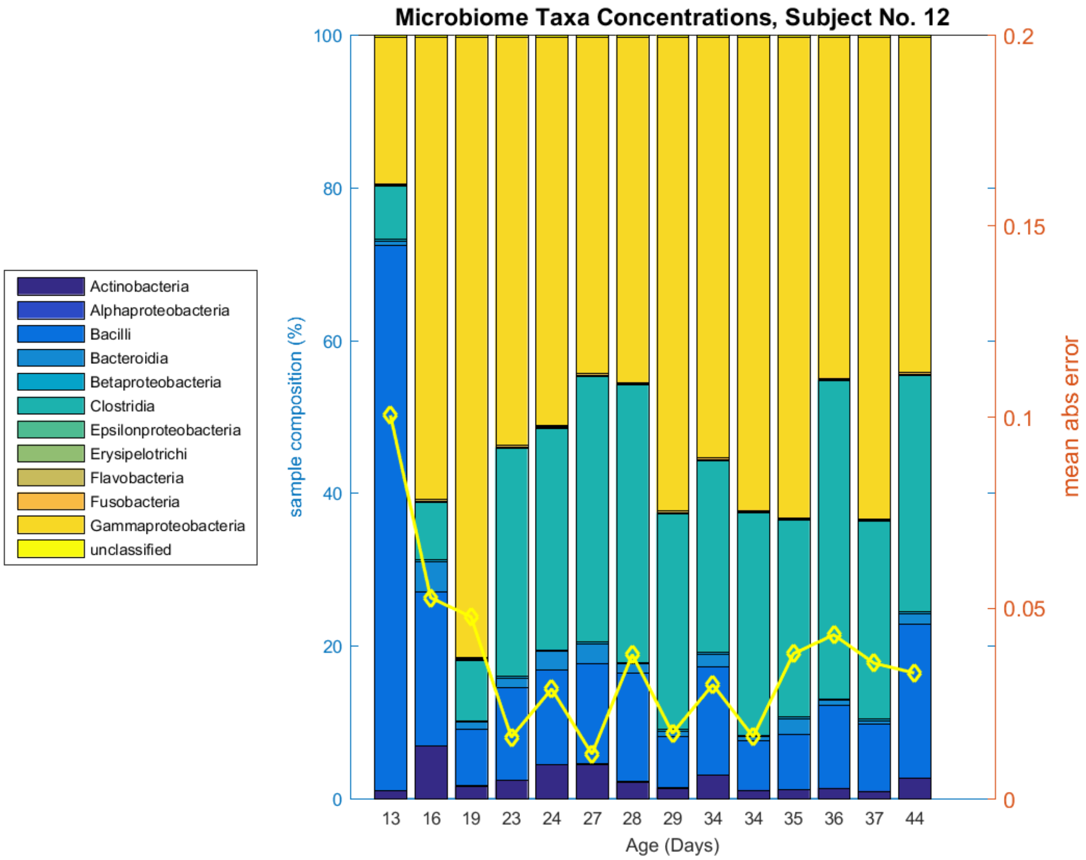

Microbiome Taxa Concentrations, Subject No. 12
Actinobacteria
Alphaproteobacteria
Bacilli
Bacteroidia
Betaproteobacteria
Clostridia
Epsilonproteobacteria
Erysipelotrichi
Flavobacteria
Fusobacteria
Gammaproteobacteria
unclassified
sample composition (%)
mean abs error
Age (Days)
100
80
60
40
20
0
0.2
0.15
0.1
0.05
0
13
16
19
23
24
27
28
29
34
34
35
36
37
44

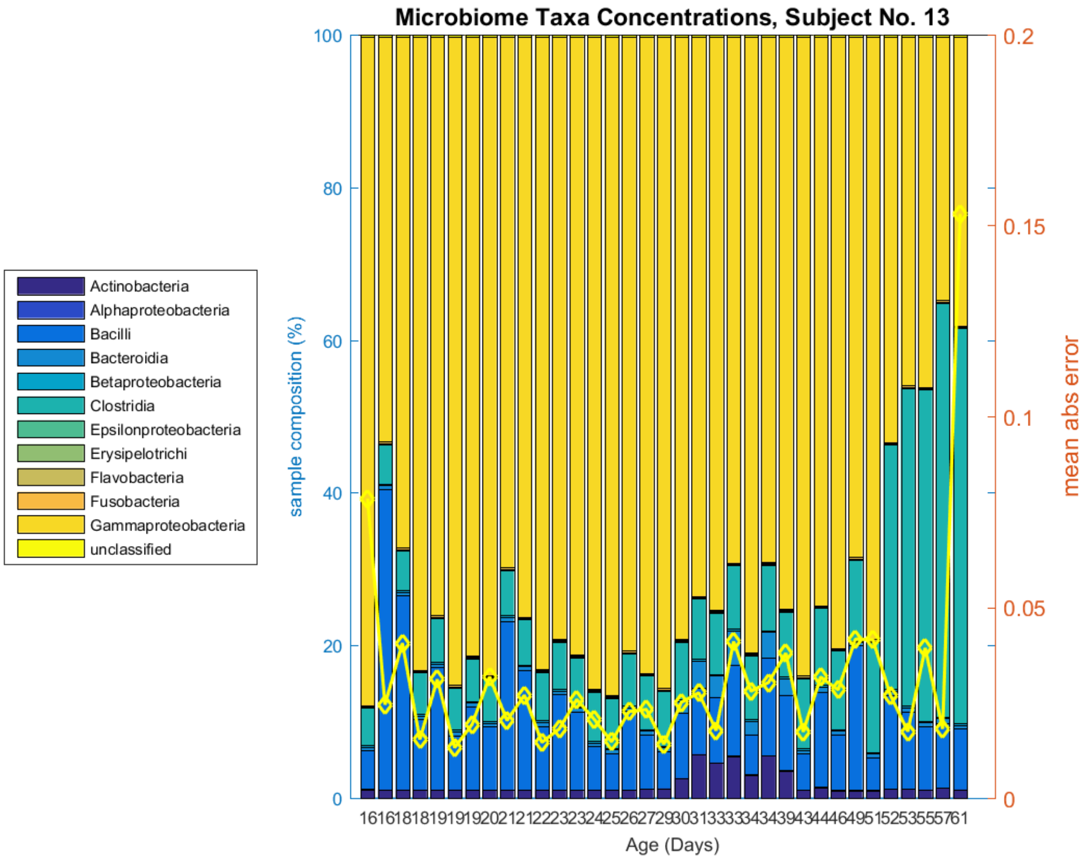
Microbiome Taxa Concentrations, Subject No. 13
Actinobacteria
Alphaproteobacteria
Bacilli
Bacteroidia
Betaproteobacteria
Clostridia
Epsilonproteobacteria
Erysipelotrichi
Flavobacteria
Fusobacteria
Gammaproteobacteria
unclassified
sample composition (%)
mean abs error
Age (Days)
0
20
40
60
80
100
0
0.05
0.1
0.15
0.2

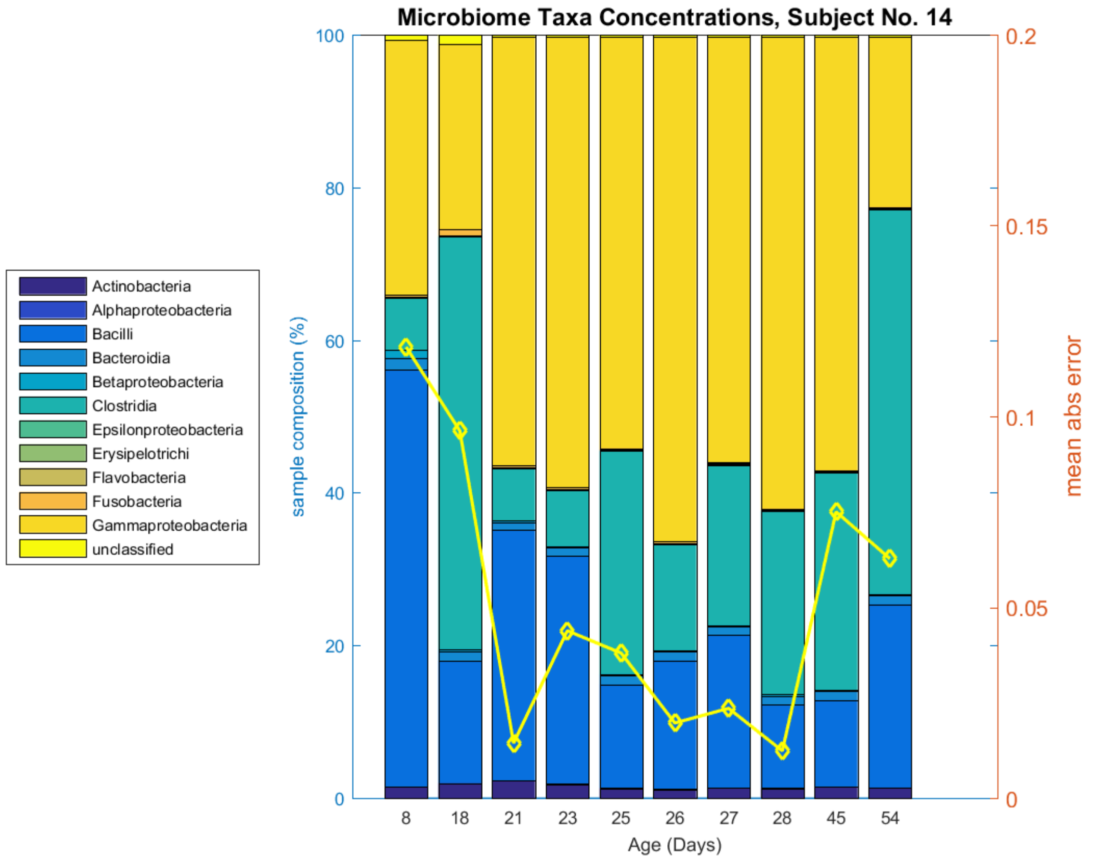

Microbiome Taxa Concentrations, Subject No. 14
sample composition (%)
mean abs error
Age (Days)
0
20
40
60
80
100
0
0.05
0.1
0.15
0.2
8
18
21
23
25
26
27
28
45
54
Actinobacteria
Alphaproteobacteria
Bacilli
Bacteroidia
Betaproteobacteria
Clostridia
Epsilonproteobacteria
Erysipelotrichi
Flavobacteria
Fusobacteria
Gammaproteobacteria
unclassified

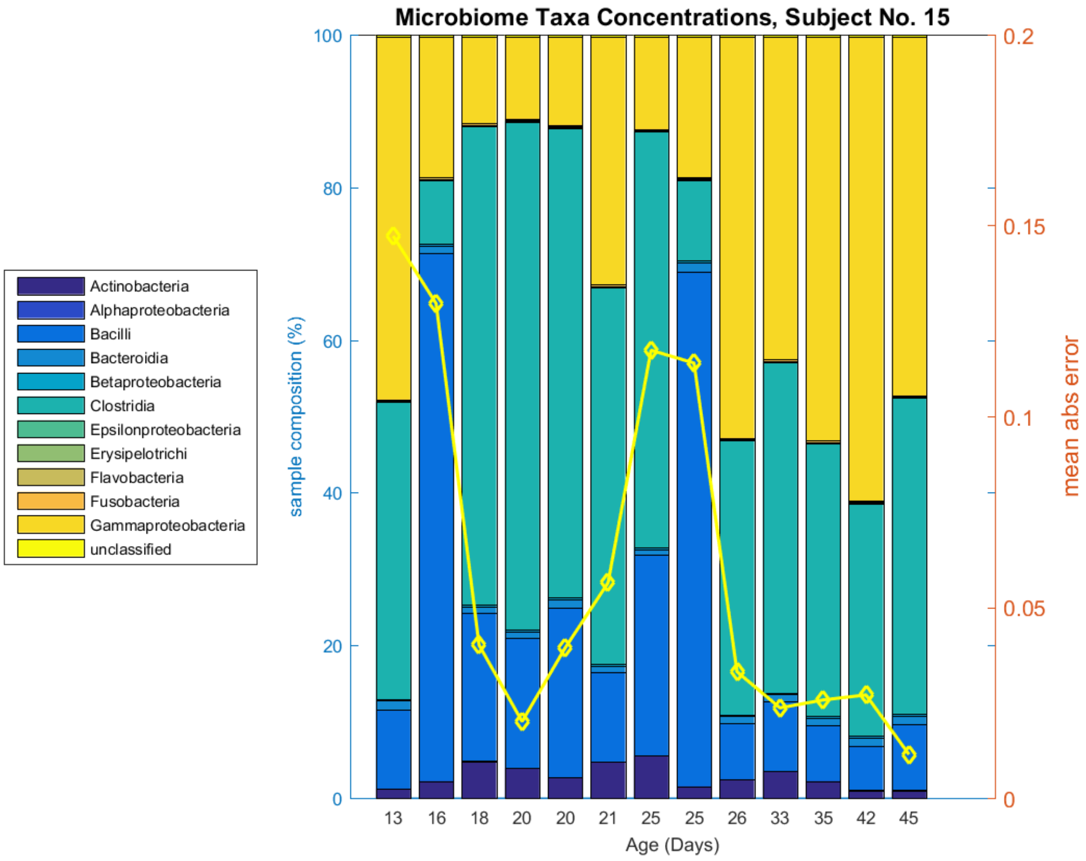

Microbiome Taxa Concentrations, Subject No. 15
Actinobacteria
Alphaproteobacteria
Bacilli
Bacteroidia
Betaproteobacteria
Clostridia
Epsilonproteobacteria
Erysipelotrichi
Flavobacteria
Fusobacteria
Gammaproteobacteria
unclassified
sample composition (%)
mean abs error
Age (Days)
13 16 18 20 20 21 25 25 26 33 35 42 45
0 20 40 60 80 100
0 0.05 0.1 0.15 0.2

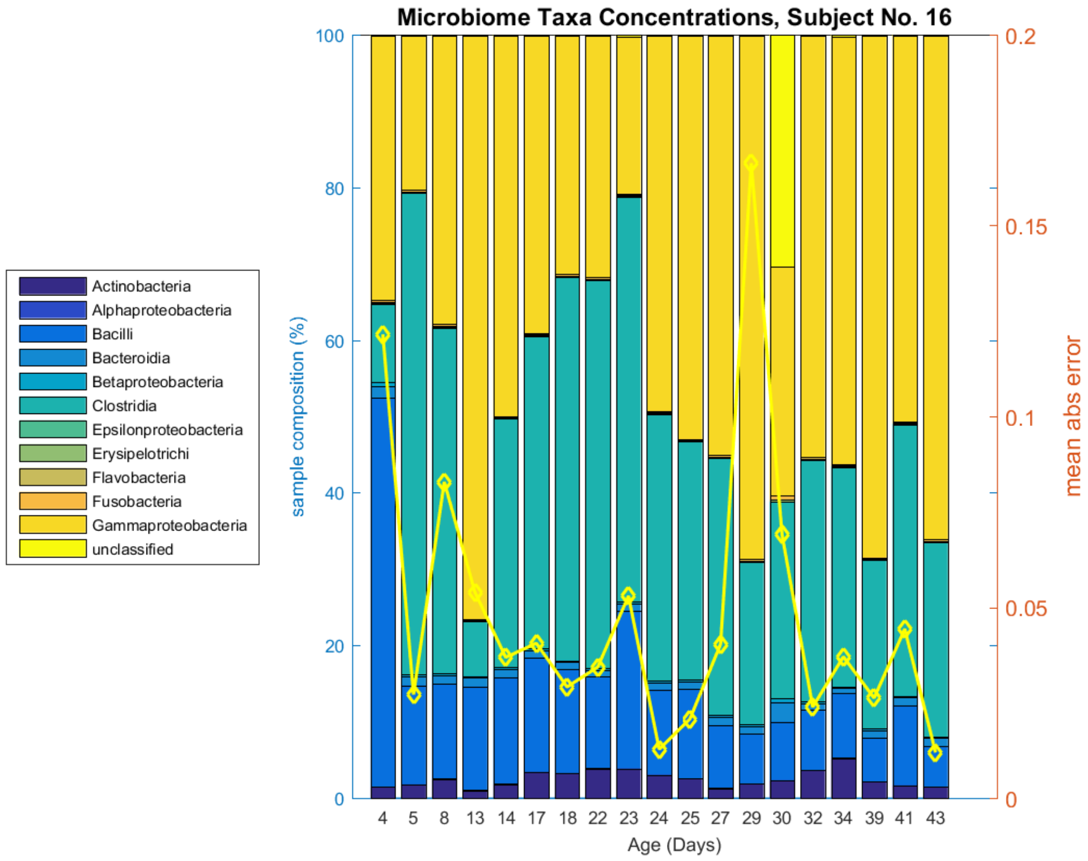

Microbiome Taxa Concentrations, Subject No. 16
Actinobacteria
Alphaproteobacteria
Bacilli
Bacteroidia
Betaproteobacteria
Clostridia
Epsilonproteobacteria
Erysipelotrichi
Flavobacteria
Fusobacteria
Gammaproteobacteria
unclassified
sample composition (%)
mean abs error
Age (Days)
0
20
40
60
80
100
0.05
0.1
0.15
0.2
4 5 8 13 14 17 18 22 23 24 25 27 29 30 32 34 39 41 43

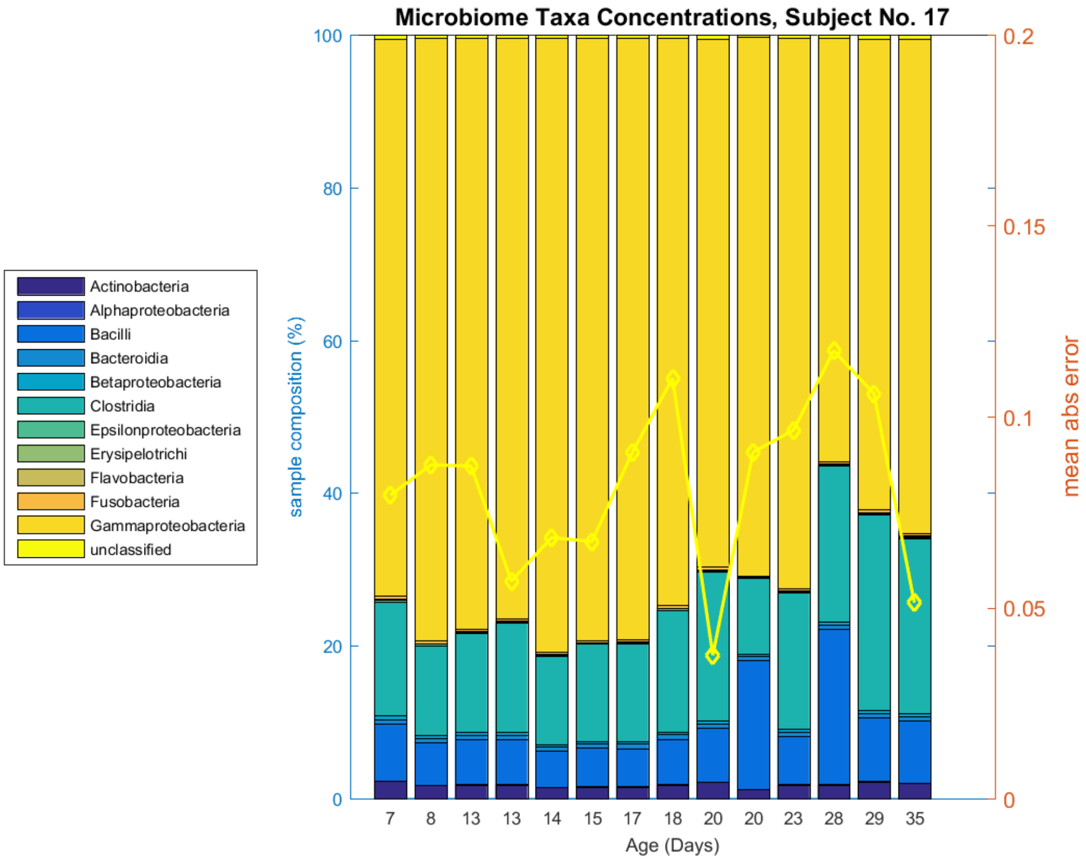

Microbiome Taxa Concentrations, Subject No. 17
Actinobacteria
Alphaproteobacteria
Bacilli
Bacteroidia
Betaproteobacteria
Clostridia
Epsilonproteobacteria
Erysipelotrichi
Flavobacteria
Fusobacteria
Gammaproteobacteria
unclassified
sample composition (%)
0
20
40
60
80
100
mean abs error
0
0.05
0.1
0.15
0.2
7
8
13
13
14
15
17
18
20
20
23
28
29
35
Age (Days)

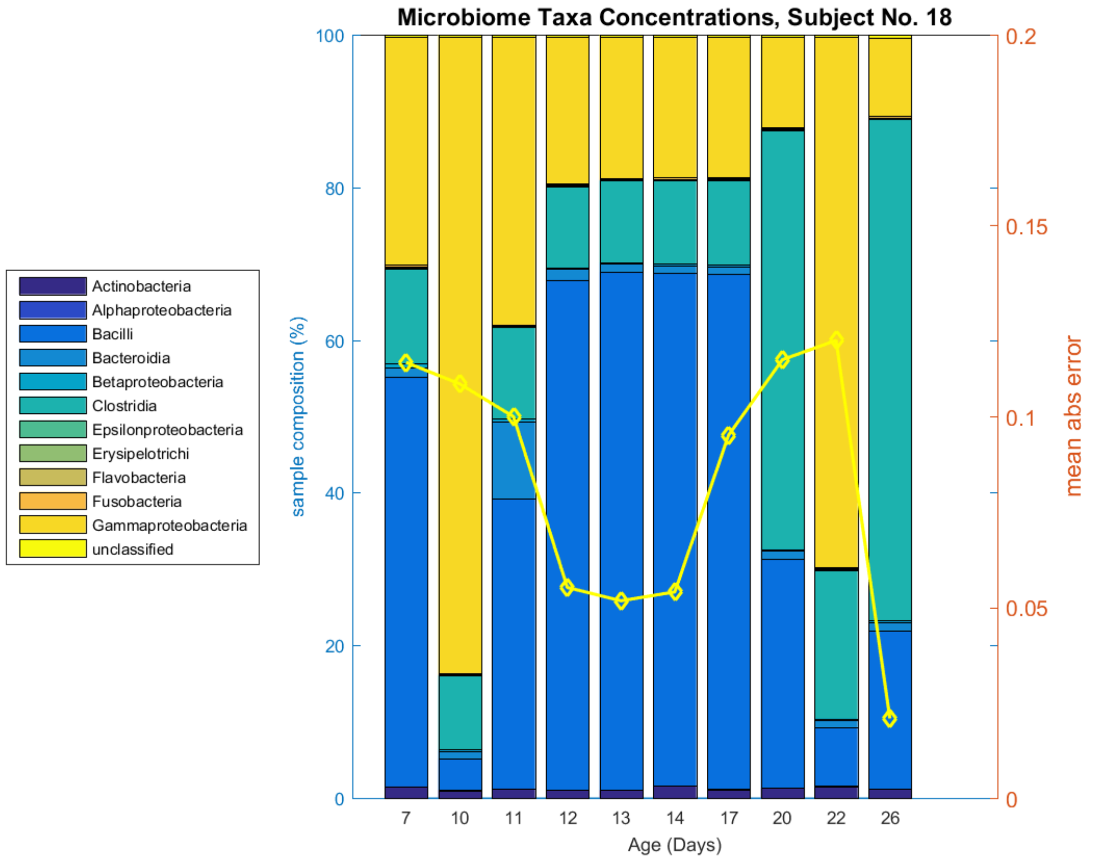

Microbiome Taxa Concentrations, Subject No. 18
sample composition (%)
mean abs error
Age (Days)
7
10
11
12
13
14
17
20
22
26
0
20
40
60
80
100
0
0.05
0.1
0.15
0.2
Actinobacteria
Alphaproteobacteria
Bacilli
Bacteroidia
Betaproteobacteria
Clostridia
Epsilonproteobacteria
Erysipelotrichi
Flavobacteria
Fusobacteria
Gammaproteobacteria
unclassified

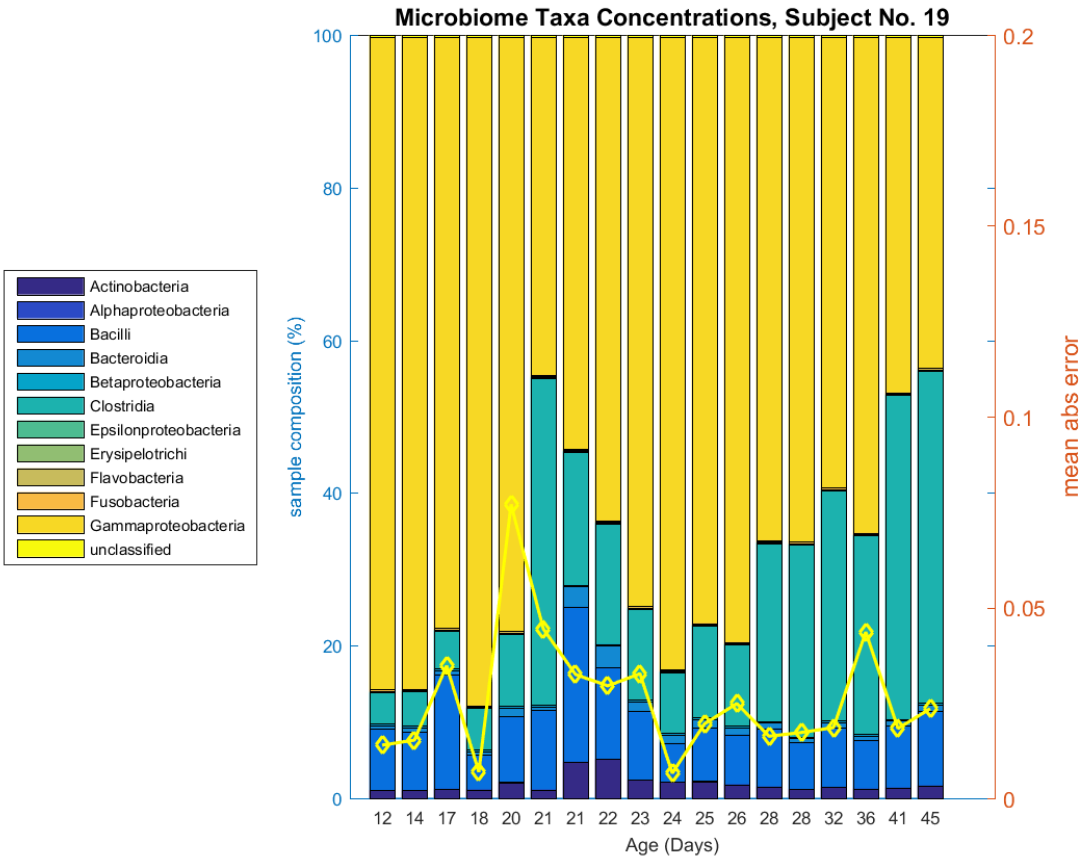
Microbiome Taxa Concentrations, Subject No. 19
Actinobacteria
Alphaproteobacteria
Bacilli
Bacteroidia
Betaproteobacteria
Clostridia
Epsilonproteobacteria
Erysipelotrichi
Flavobacteria
Fusobacteria
Gammaproteobacteria
unclassified
sample composition (%)
mean abs error
Age (Days)
12 14 17 18 20 21 21 22 23 24 25 26 28 28 32 36 41 45
0 20 40 60 80 100
0 0.05 0.1 0.15 0.2

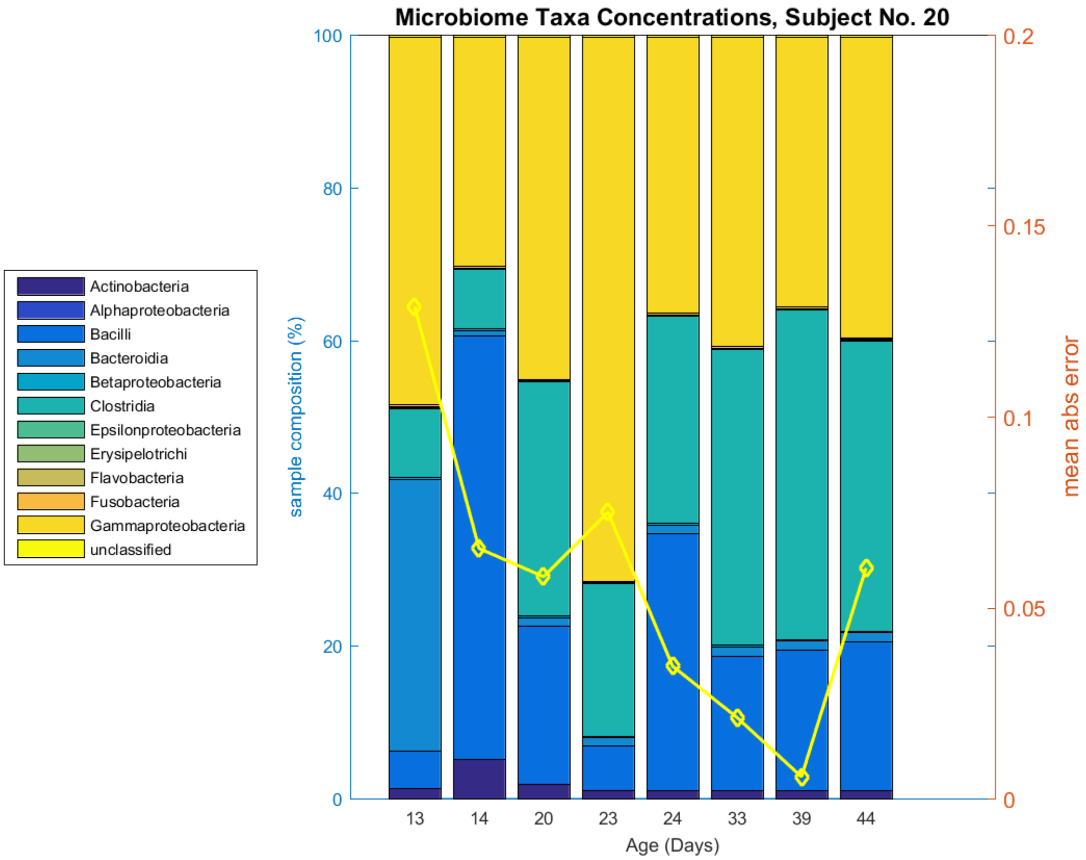

Microbiome Taxa Concentrations, Subject No. 20
Actinobacteria
Alphaproteobacteria
Bacilli
Bacteroidia
Betaproteobacteria
Clostridia
Epsilonproteobacteria
Erysipelotrichi
Flavobacteria
Fusobacteria
Gammaproteobacteria
unclassified
sample composition (%)
100
80
60
40
20
0
mean abs error
0.2
0.15
0.1
0.05
0
13
14
20
23
24
33
39
44
Age (Days)

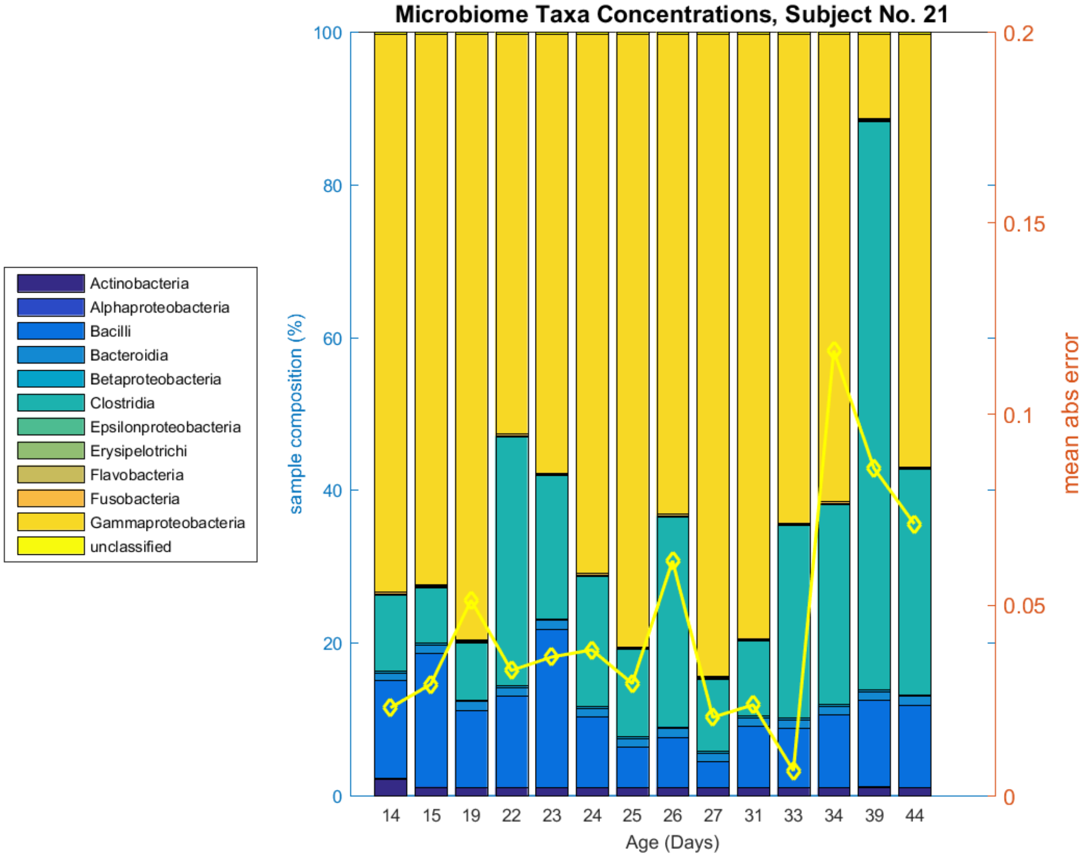

Microbiome Taxa Concentrations, Subject No. 21
sample composition (%)
mean abs error
Age (Days)
0
20
40
60
80
100
0
0.05
0.1
0.15
0.2
14
15
19
22
23
24
25
26
27
31
33
34
39
44
Actinobacteria
Alphaproteobacteria
Bacilli
Bacteroidia
Betaproteobacteria
Clostridia
Epsilonproteobacteria
Erysipelotrichi
Flavobacteria
Fusobacteria
Gammaproteobacteria
unclassified

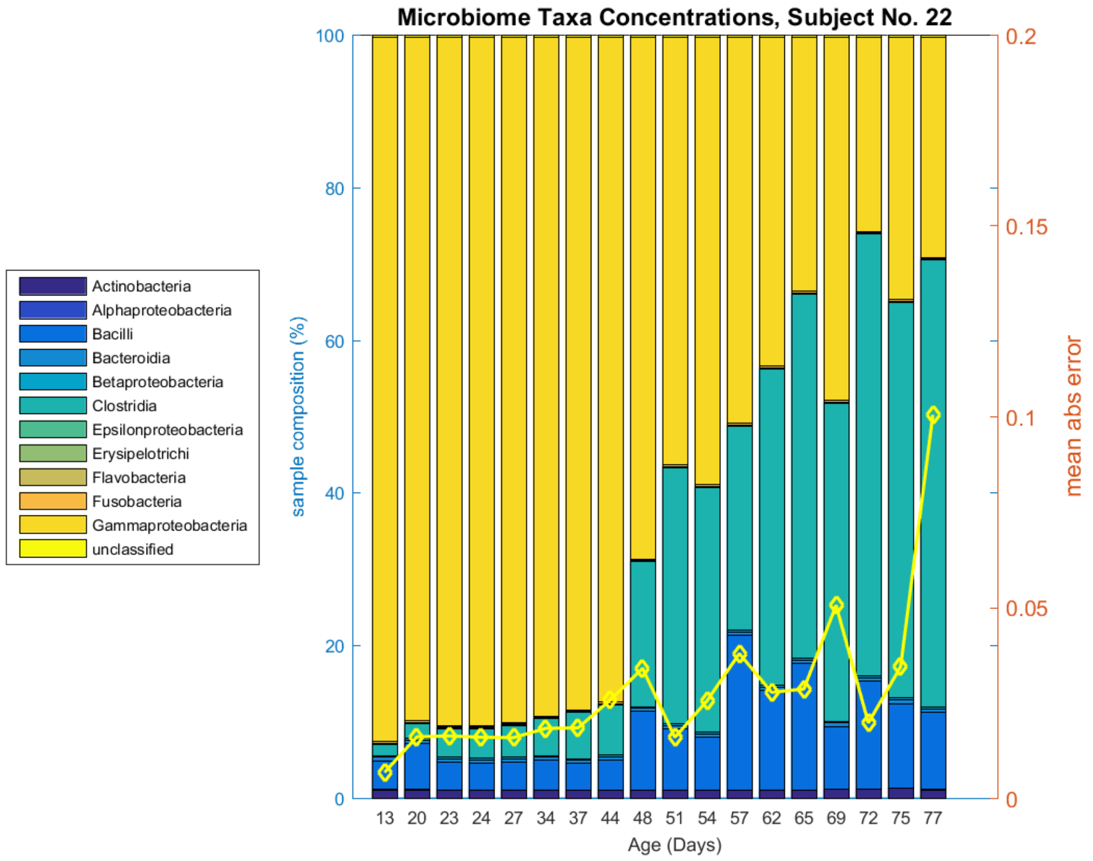

Microbiome Taxa Concentrations, Subject No. 22
Actinobacteria
Alphaproteobacteria
Bacilli
Bacteroidia
Betaproteobacteria
Clostridia
Epsilonproteobacteria
Erysipelotrichi
Flavobacteria
Fusobacteria
Gammaproteobacteria
unclassified
sample composition (%)
mean abs error
100
80
60
40
20
0
0.2
0.15
0.1
0.05
0
13 20 23 24 27 34 37 44 48 51 54 57 62 65 69 72 75 77
Age (Days)

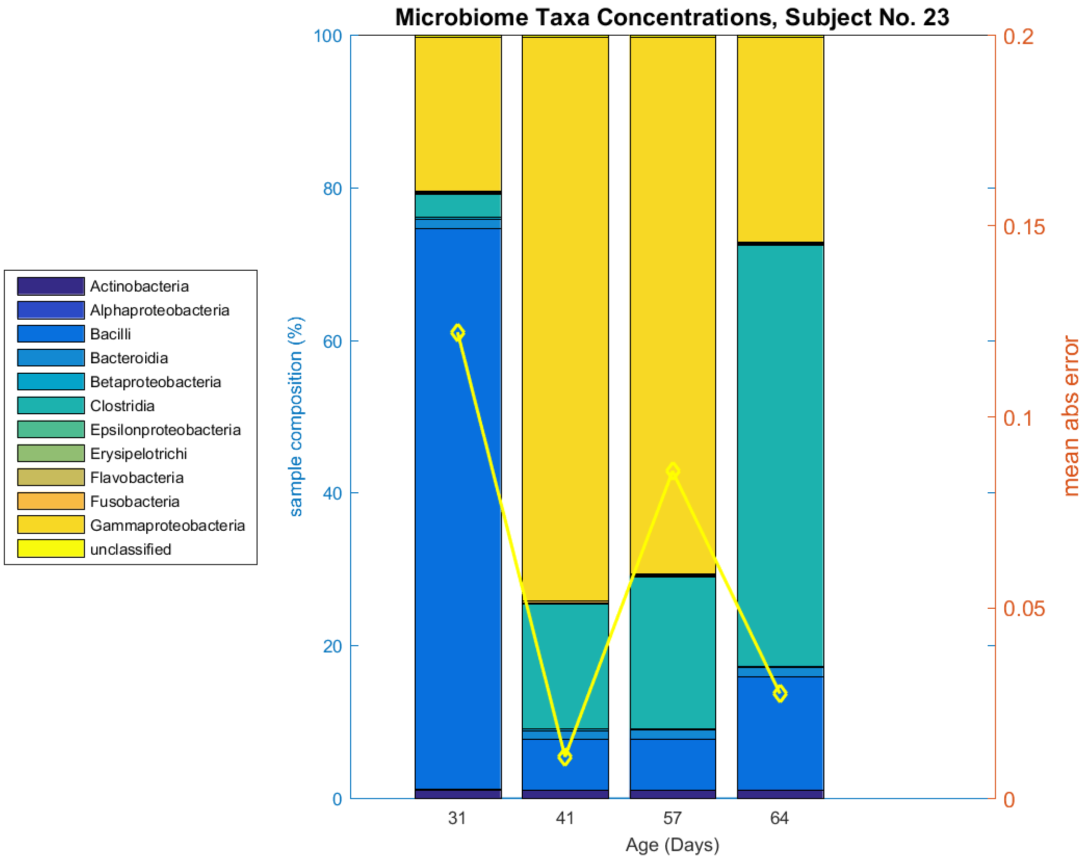

Microbiome Taxa Concentrations, Subject No. 23
Actinobacteria
Alphaproteobacteria
Bacilli
Bacteroidia
Betaproteobacteria
Clostridia
Epsilonproteobacteria
Erysipelotrichi
Flavobacteria
Fusobacteria
Gammaproteobacteria
unclassified
sample composition (%)
mean abs error
Age (Days)
100
80
60
40
20
0
0.2
0.15
0.1
0.05
0
31
41
57
64

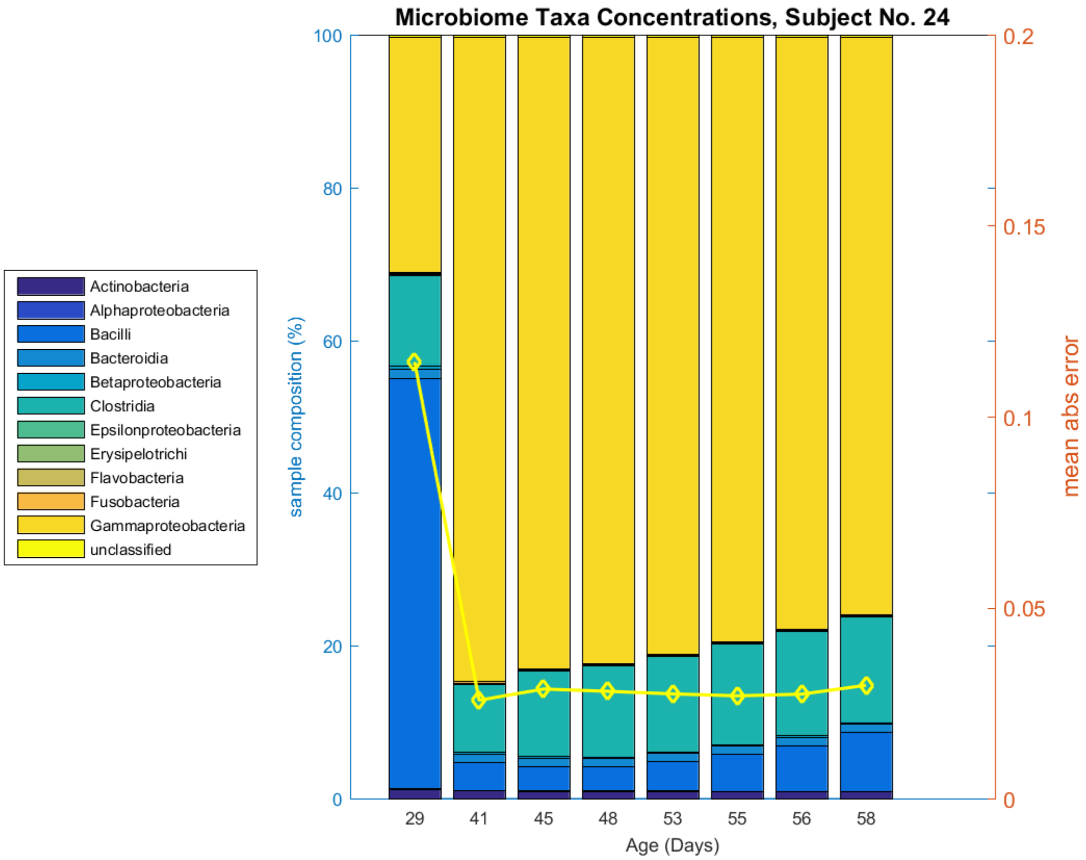

Microbiome Taxa Concentrations, Subject No. 24
Actinobacteria
Alphaproteobacteria
Bacilli
Bacteroidia
Betaproteobacteria
Clostridia
Epsilonproteobacteria
Erysipelotrichi
Flavobacteria
Fusobacteria
Gammaproteobacteria
unclassified
sample composition (%)
mean abs error
Age (Days)
0
20
40
60
80
100
0
0.05
0.1
0.15
0.2
29
41
45
48
53
55
56
58

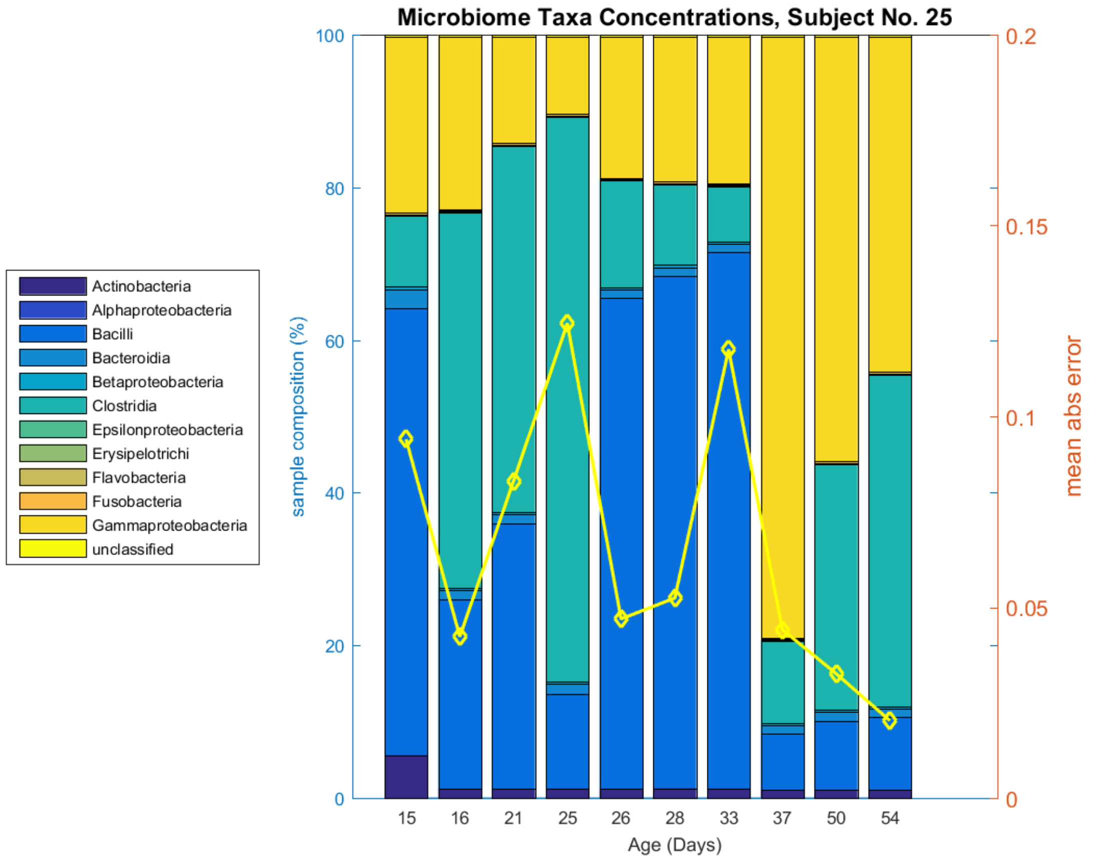

Microbiome Taxa Concentrations, Subject No. 25
Actinobacteria
Alphaproteobacteria
Bacilli
Bacteroidia
Betaproteobacteria
Clostridia
Epsilonproteobacteria
Erysipelotrichi
Flavobacteria
Fusobacteria
Gammaproteobacteria
unclassified
sample composition (%)
0
20
40
60
80
100
mean abs error
0
0.05
0.1
0.15
0.2
15
16
21
25
26
28
33
37
50
54
Age (Days)

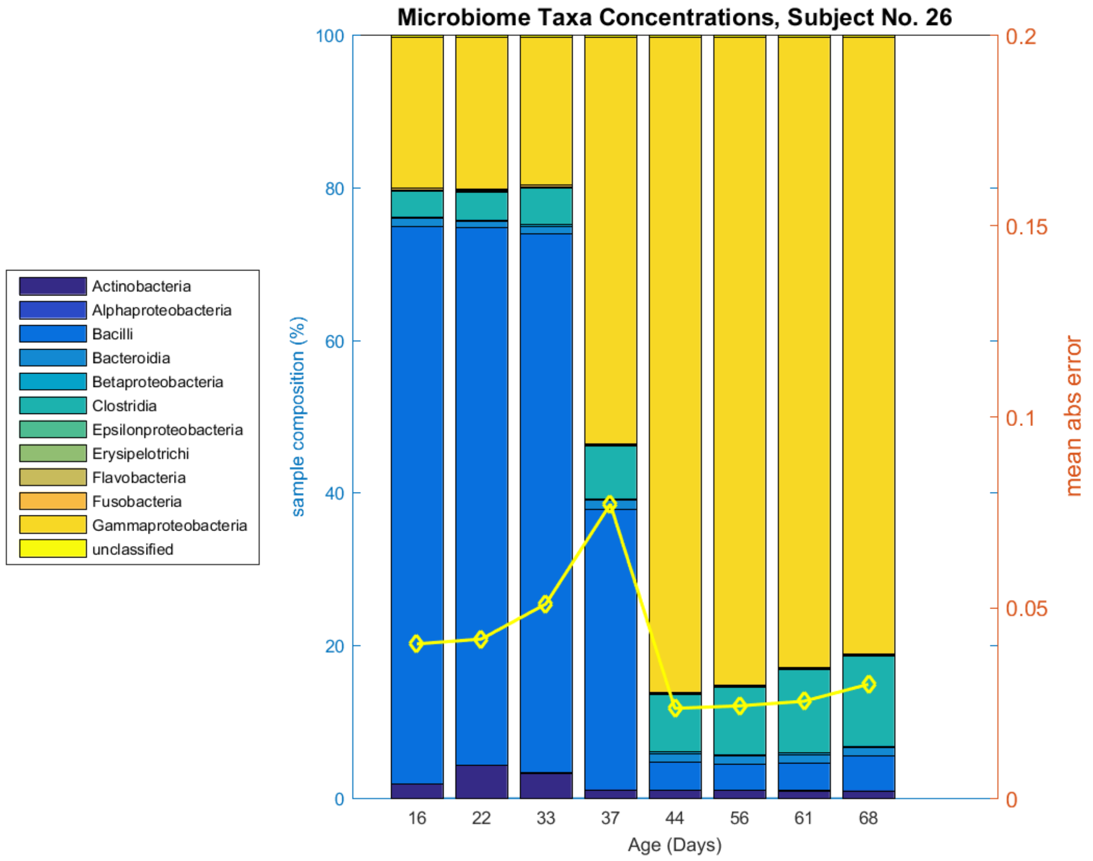

Microbiome Taxa Concentrations, Subject No. 26
Actinobacteria
Alphaproteobacteria
Bacilli
Bacteroidia
Betaproteobacteria
Clostridia
Epsilonproteobacteria
Erysipelotrichi
Flavobacteria
Fusobacteria
Gammaproteobacteria
unclassified
sample composition (%)
mean abs error
Age (Days)
16
22
33
37
44
56
61
68
0
20
40
60
80
100
0
0.05
0.1
0.15
0.2

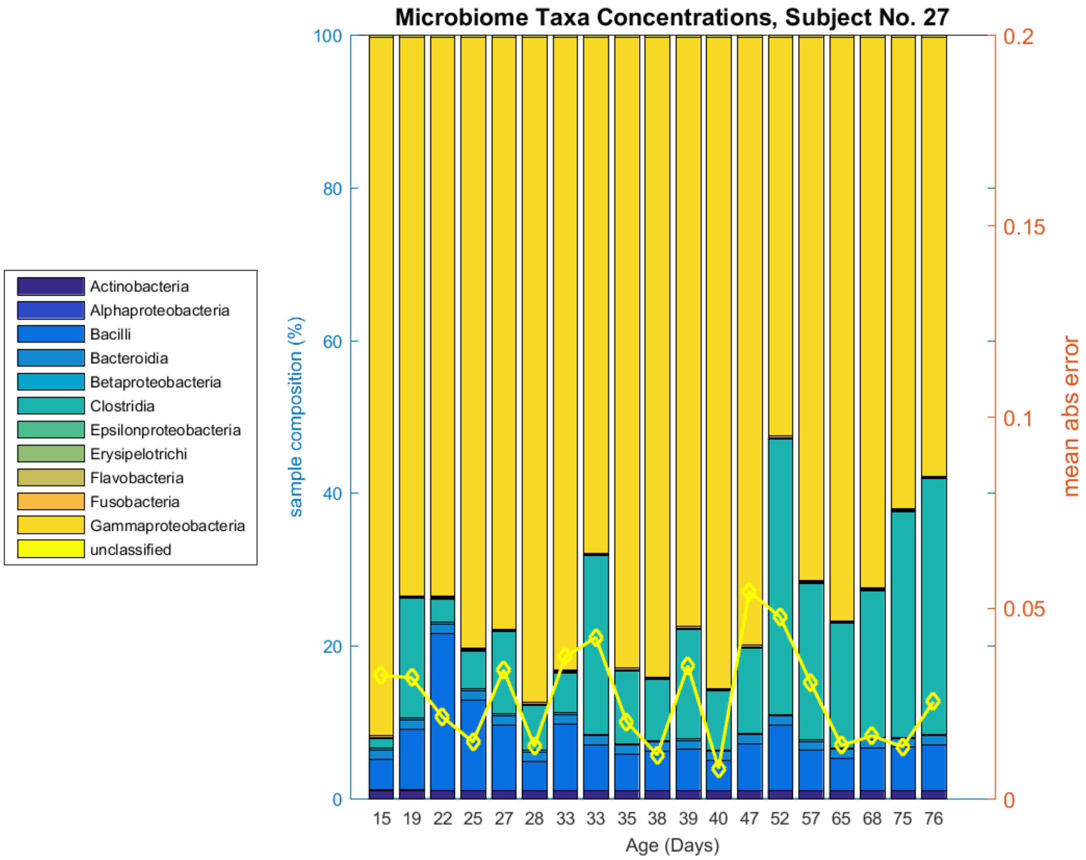

Microbiome Taxa Concentrations, Subject No. 27
Actinobacteria
Alphaproteobacteria
Bacilli
Bacteroidia
Betaproteobacteria
Clostridia
Epsilonproteobacteria
Erysipelotrichi
Flavobacteria
Fusobacteria
Gammaproteobacteria
unclassified
sample composition (%)
mean abs error
0
20
40
60
80
100
0
0.05
0.1
0.15
0.2
15 19 22 25 27 28 33 33 35 38 39 40 47 52 57 65 68 75 76
Age (Days)

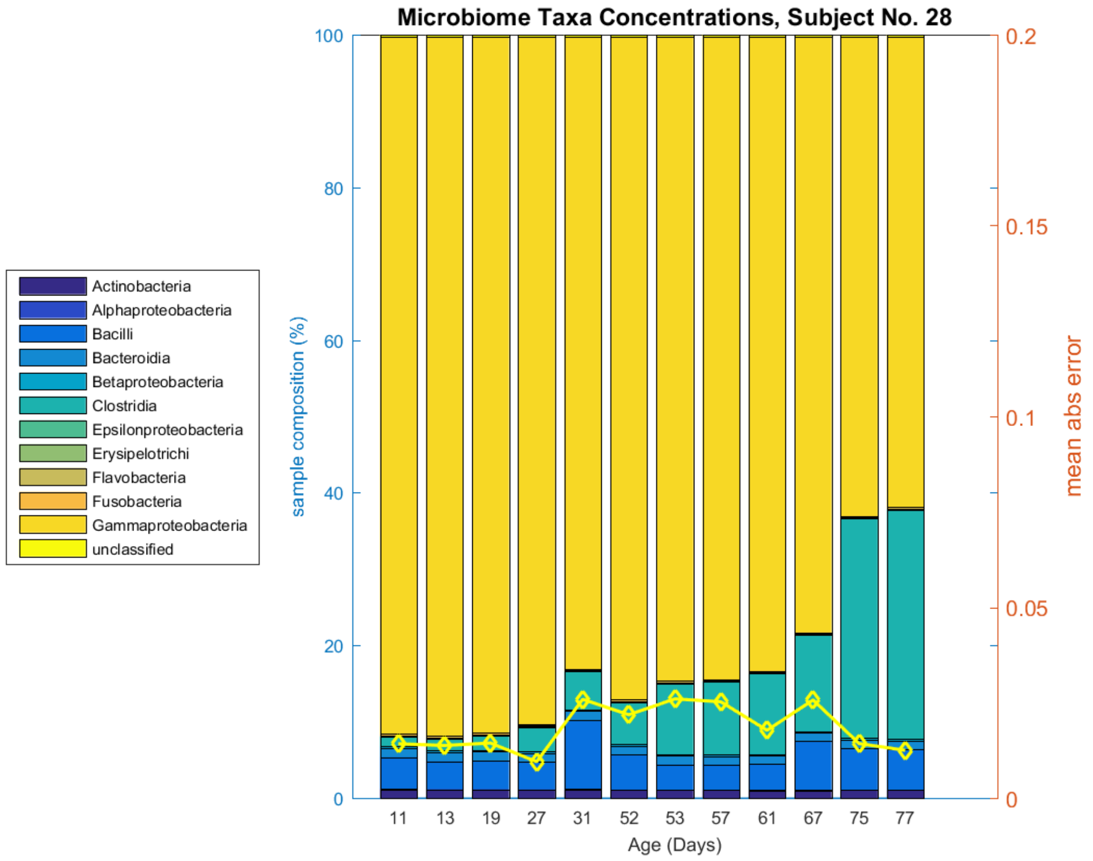

Microbiome Taxa Concentrations, Subject No. 28
Actinobacteria
Alphaproteobacteria
Bacilli
Bacteroidia
Betaproteobacteria
Clostridia
Epsilonproteobacteria
Erysipelotrichi
Flavobacteria
Fusobacteria
Gammaproteobacteria
unclassified
sample composition (%)
0
20
40
60
80
100
mean abs error
0
0.05
0.1
0.15
0.2
11
13
19
27
31
52
53
57
61
67
75
77
Age (Days)

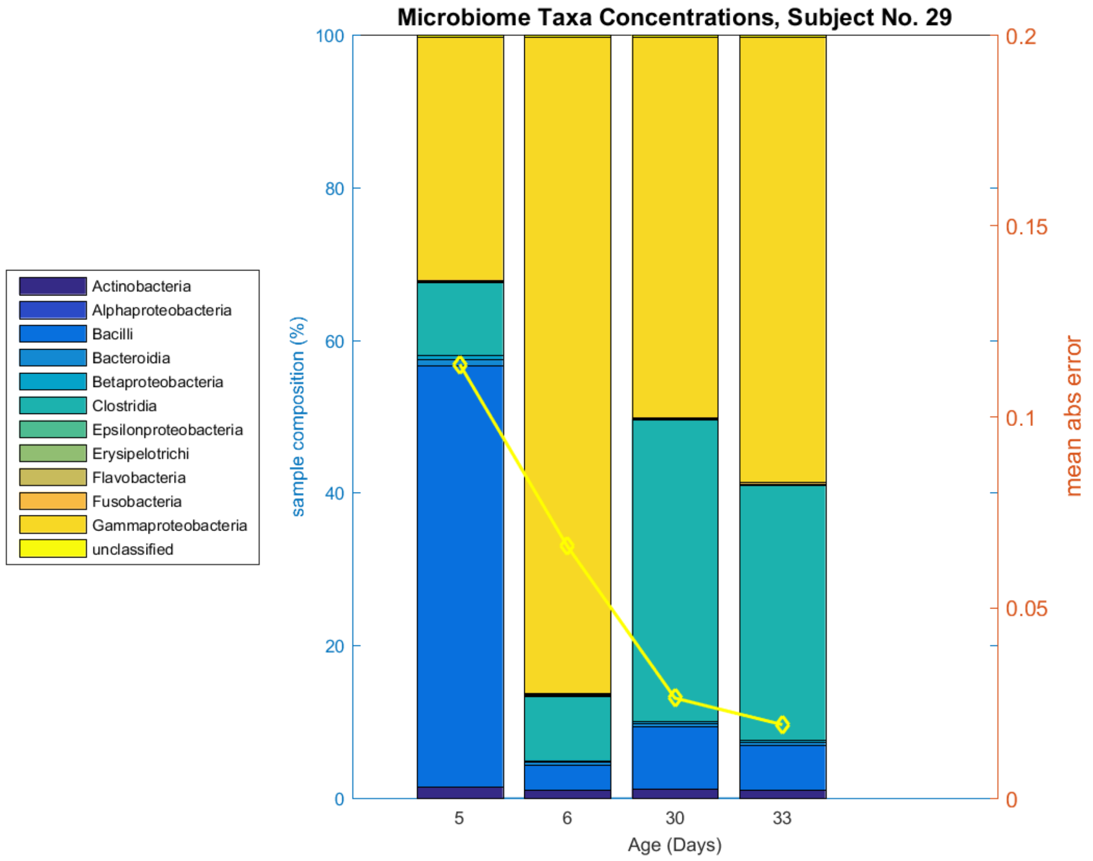
Microbiome Taxa Concentrations, Subject No. 29
Actinobacteria
Alphaproteobacteria
Bacilli
Bacteroidia
Betaproteobacteria
Clostridia
Epsilonproteobacteria
Erysipelotrichi
Flavobacteria
Fusobacteria
Gammaproteobacteria
unclassified
sample composition (%)
mean abs error
Age (Days)
5
6
30
33
0
20
40
60
80
100
0
0.05
0.1
0.15
0.2

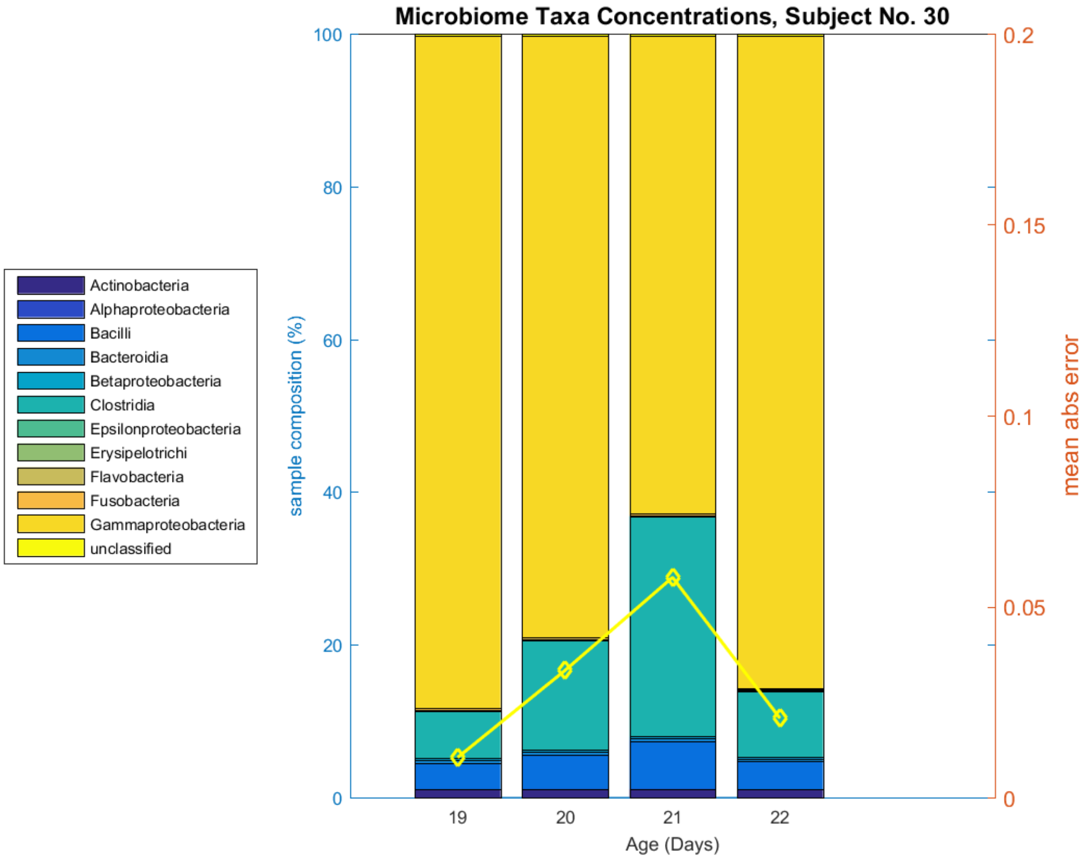

Microbiome Taxa Concentrations, Subject No. 30
Actinobacteria
Alphaproteobacteria
Bacilli
Bacteroidia
Betaproteobacteria
Clostridia
Epsilonproteobacteria
Erysipelotrichi
Flavobacteria
Fusobacteria
Gammaproteobacteria
unclassified
sample composition (%)
mean abs error
Age (Days)
0
20
40
60
80
100
0
0.05
0.1
0.15
0.2
19
20
21
22

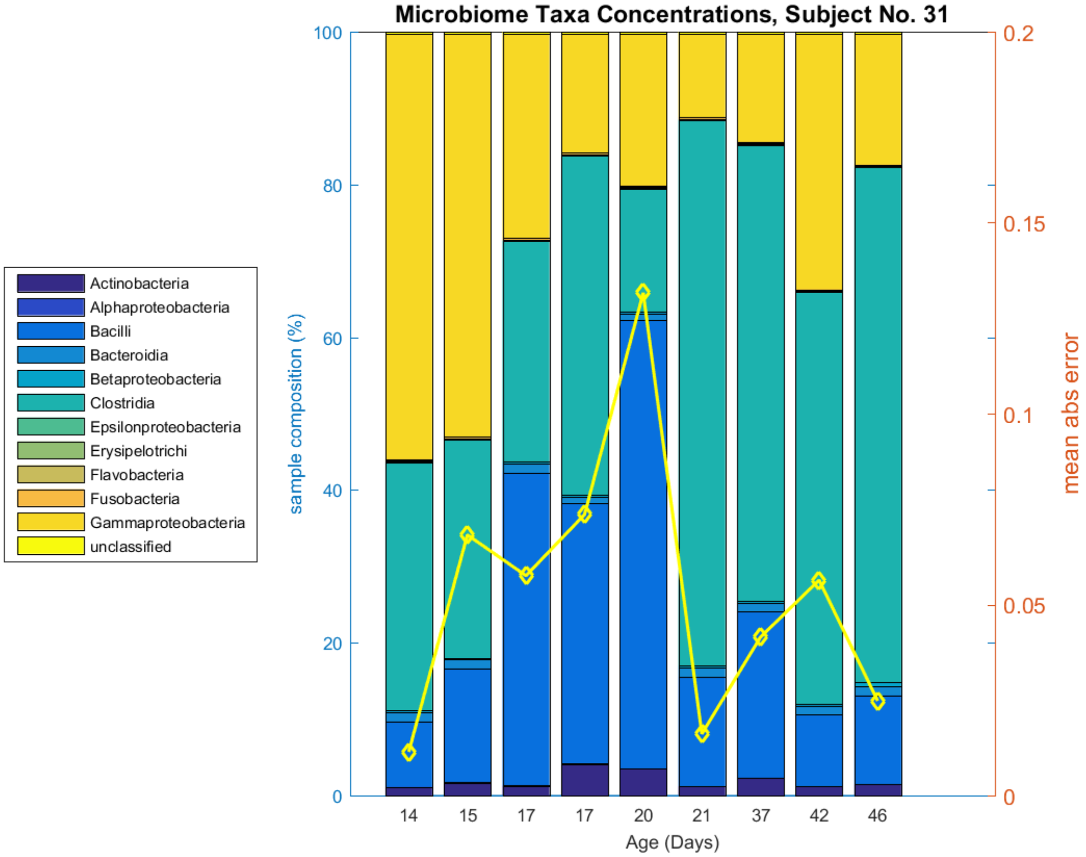
Microbiome Taxa Concentrations, Subject No. 31
sample composition (%)
mean abs error
Age (Days)
14
15
17
17
20
21
37
42
46
Actinobacteria
Alphaproteobacteria
Bacilli
Bacteroidia
Betaproteobacteria
Clostridia
Epsilonproteobacteria
Erysipelotrichi
Flavobacteria
Fusobacteria
Gammaproteobacteria
unclassified

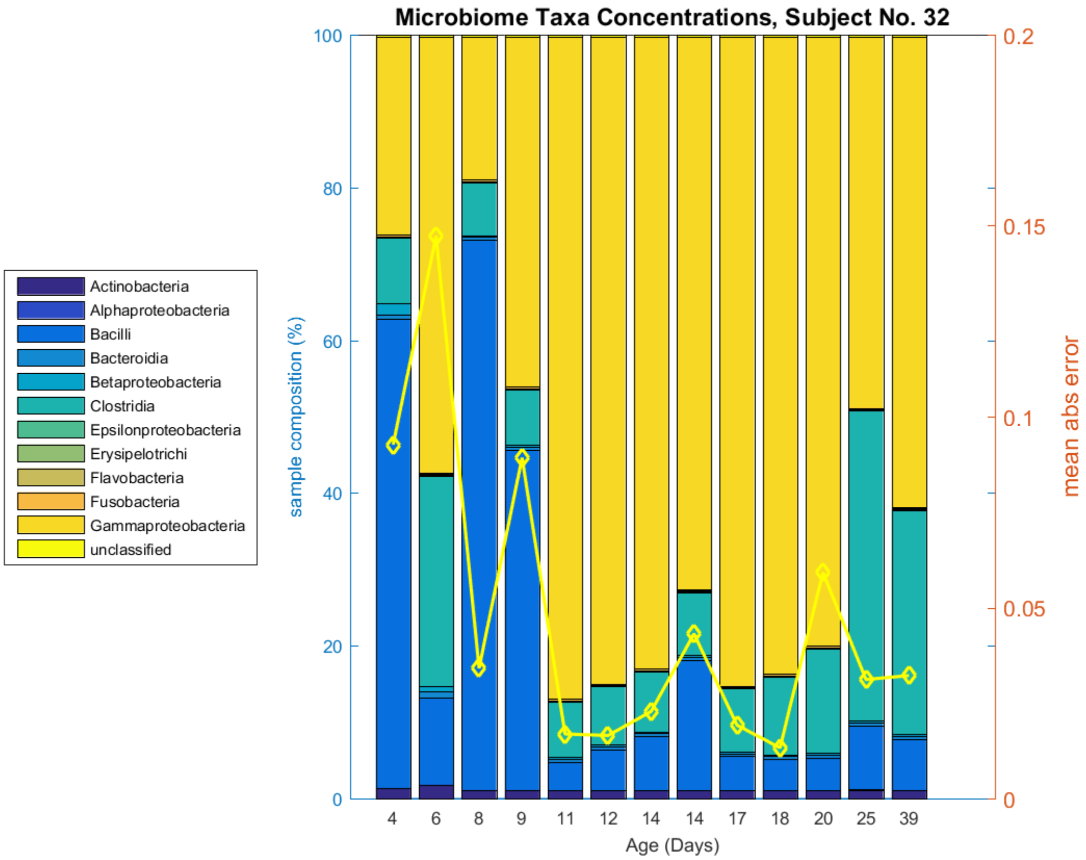

Microbiome Taxa Concentrations, Subject No. 32
Actinobacteria
Alphaproteobacteria
Bacilli
Bacteroidia
Betaproteobacteria
Clostridia
Epsilonproteobacteria
Erysipelotrichi
Flavobacteria
Fusobacteria
Gammaproteobacteria
unclassified
sample composition (%)
100
80
60
40
20
0
mean abs error
0.2
0.15
0.1
0.05
0
4
6
8
9
11
12
14
14
17
18
20
25
39
Age (Days)

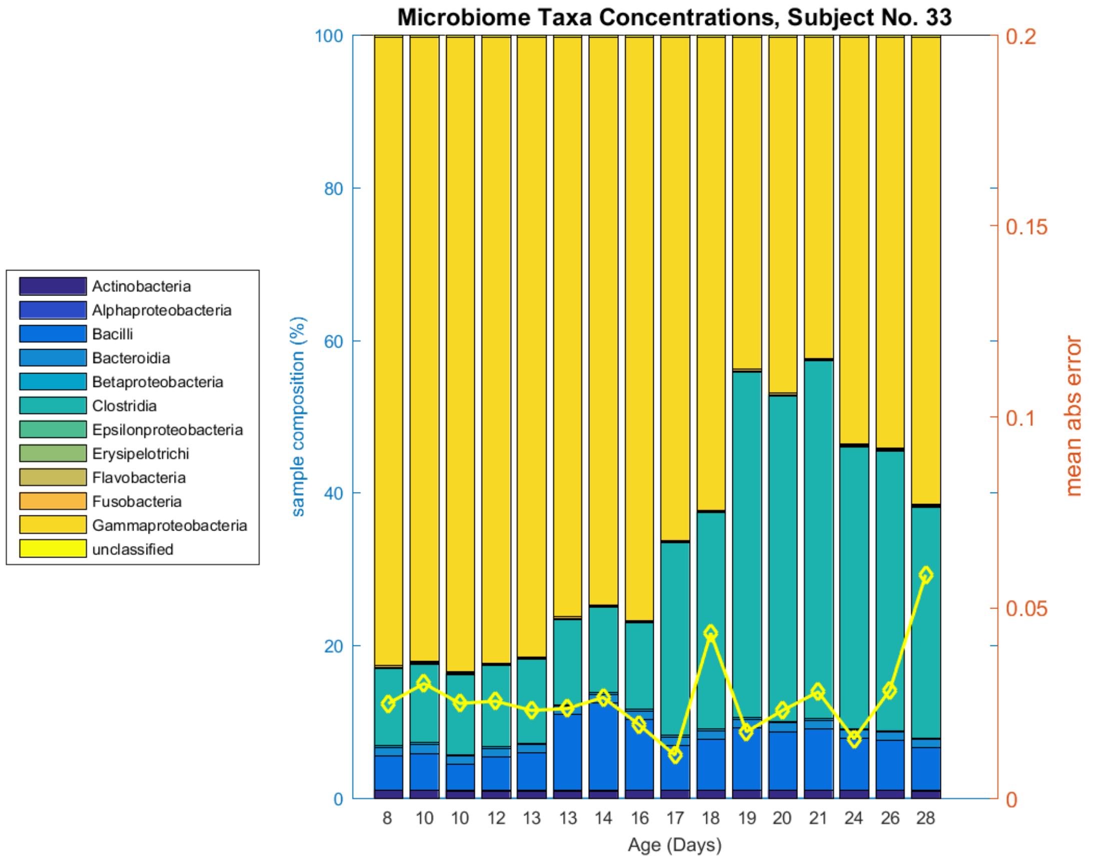

Microbiome Taxa Concentrations, Subject No. 33
sample composition (%)
mean abs error
Age (Days)
0
20
40
60
80
100
0
0.05
0.1
0.15
0.2
8
10
10
12
13
13
14
16
17
18
19
20
21
24
26
28
Actinobacteria
Alphaproteobacteria
Bacilli
Bacteroidia
Betaproteobacteria
Clostridia
Epsilonproteobacteria
Erysipelotrichi
Flavobacteria
Fusobacteria
Gammaproteobacteria
unclassified

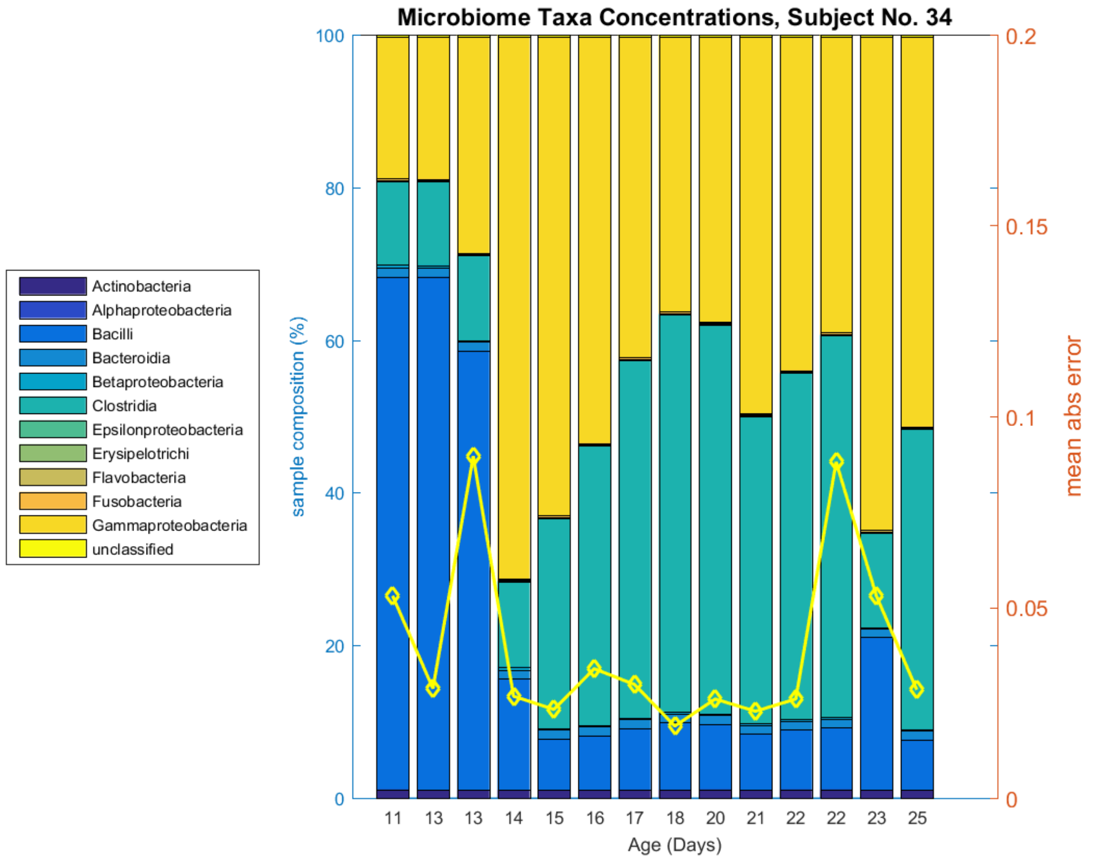

Microbiome Taxa Concentrations, Subject No. 34
Actinobacteria
Alphaproteobacteria
Bacilli
Bacteroidia
Betaproteobacteria
Clostridia
Epsilonproteobacteria
Erysipelotrichi
Flavobacteria
Fusobacteria
Gammaproteobacteria
unclassified
sample composition (%)
0
20
40
60
80
100
mean abs error
0
0.05
0.1
0.15
0.2
11
13
13
14
15
16
17
18
20
21
22
22
23
25
Age (Days)

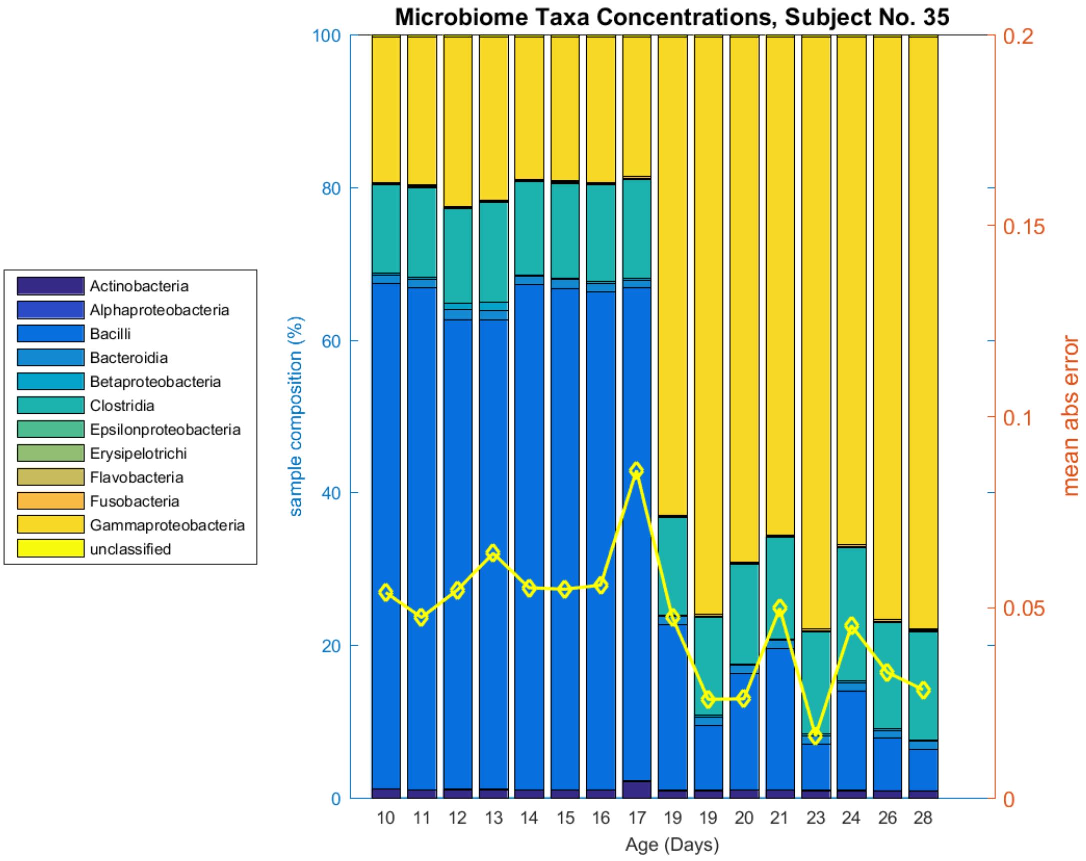

Microbiome Taxa Concentrations, Subject No. 35
Actinobacteria
Alphaproteobacteria
Bacilli
Bacteroidia
Betaproteobacteria
Clostridia
Epsilonproteobacteria
Erysipelotrichi
Flavobacteria
Fusobacteria
Gammaproteobacteria
unclassified
sample composition (%)
mean abs error
Age (Days)
0
20
40
60
80
100
0
0.05
0.1
0.15
0.2
10 11 12 13 14 15 16 17 19 19 20 21 23 24 26 28

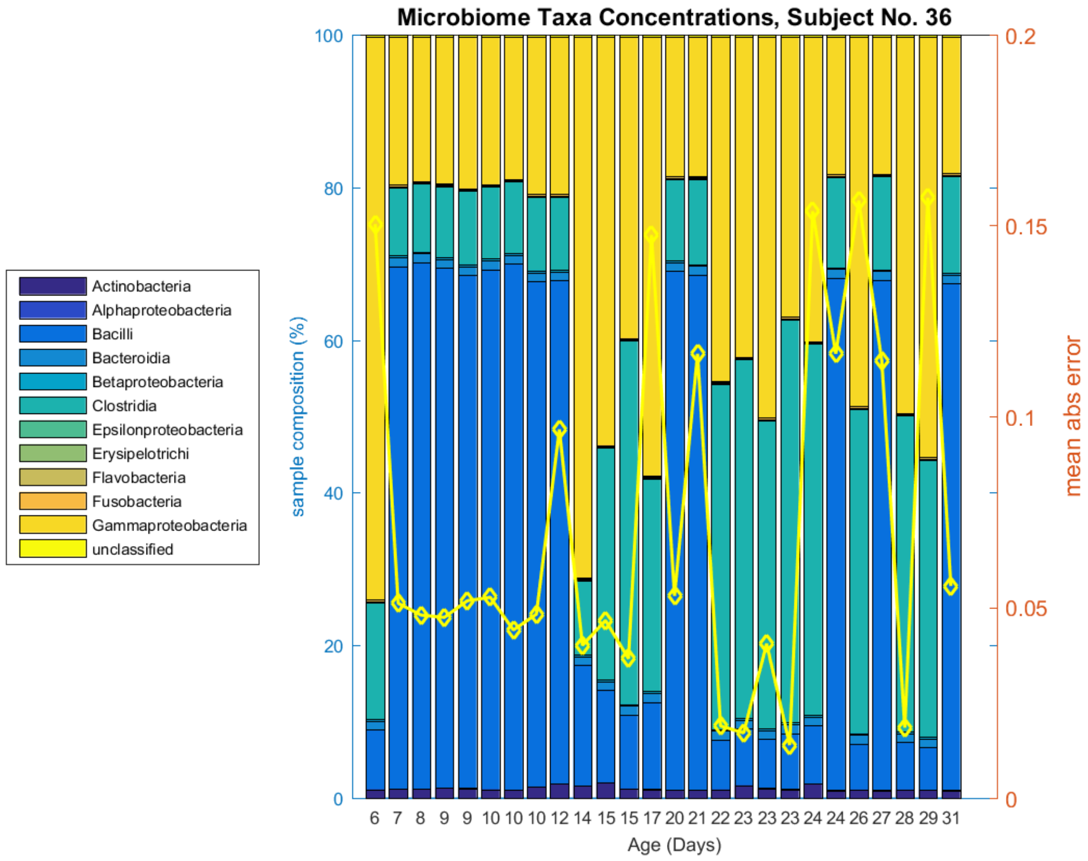
Microbiome Taxa Concentrations, Subject No. 36
Actinobacteria
Alphaproteobacteria
Bacilli
Bacteroidia
Betaproteobacteria
Clostridia
Epsilonproteobacteria
Erysipelotrichi
Flavobacteria
Fusobacteria
Gammaproteobacteria
unclassified
sample composition (%)
mean abs error
Age (Days)
0
20
40
60
80
100
0
0.05
0.1
0.15
0.2
6 7 8 9 9 10 10 10 12 14 15 15 17 20 21 22 23 23 23 24 24 26 27 28 29 31

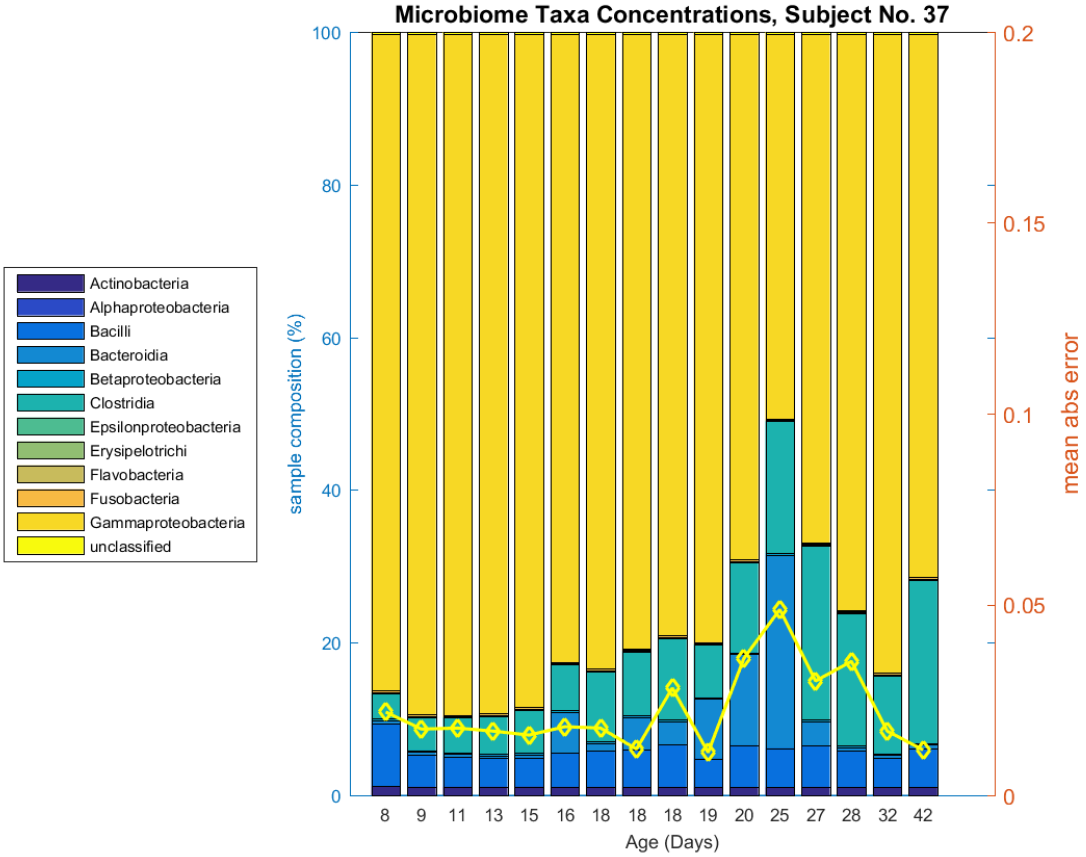
Microbiome Taxa Concentrations, Subject No. 37
Actinobacteria
Alphaproteobacteria
Bacilli
Bacteroidia
Betaproteobacteria
Clostridia
Epsilonproteobacteria
Erysipelotrichi
Flavobacteria
Fusobacteria
Gammaproteobacteria
unclassified
sample composition (%)
mean abs error
Age (Days)
0
20
40
60
80
100
0
0.05
0.1
0.15
0.2
8
9
11
13
15
16
18
18
18
19
20
25
27
28
32
42

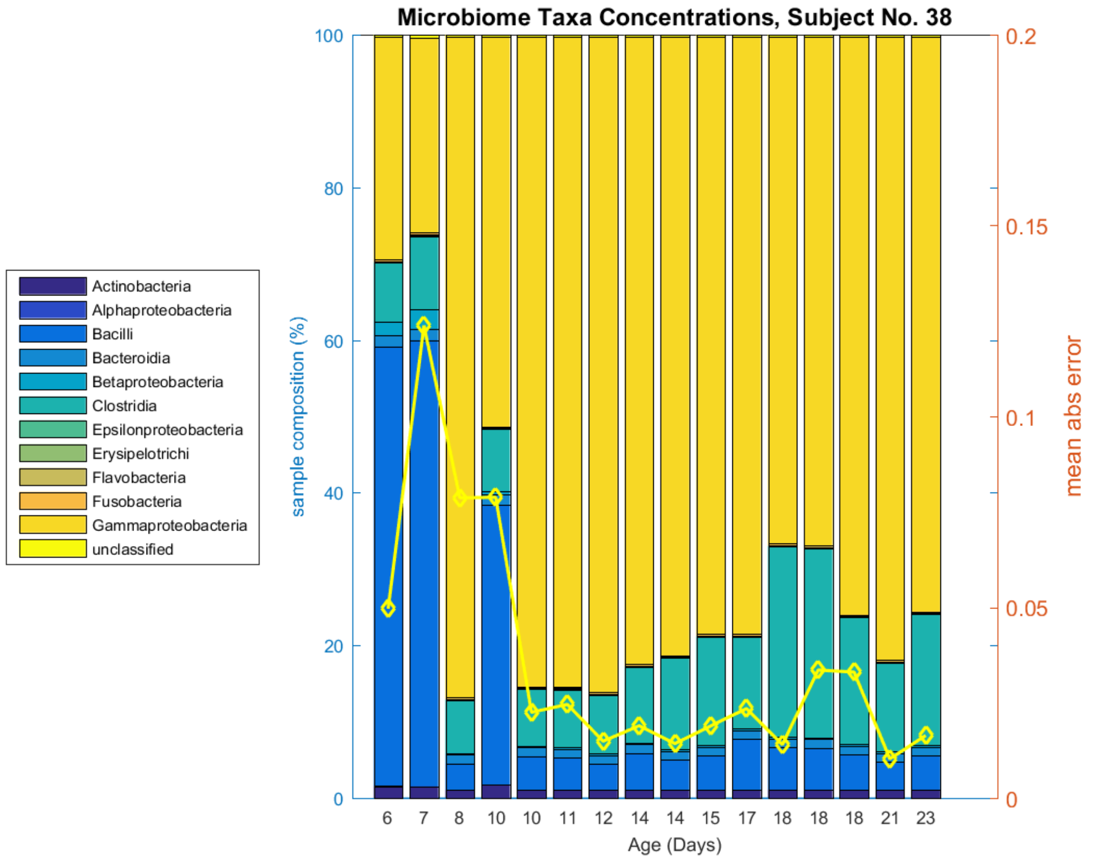
Microbiome Taxa Concentrations, Subject No. 38
Actinobacteria
Alphaproteobacteria
Bacilli
Bacteroidia
Betaproteobacteria
Clostridia
Epsilonproteobacteria
Erysipelotrichi
Flavobacteria
Fusobacteria
Gammaproteobacteria
unclassified
sample composition (%)
mean abs error
Age (Days)
6 7 8 10 10 11 12 14 14 15 17 18 18 18 21 23
0 20 40 60 80 100
0 0.05 0.1 0.15 0.2

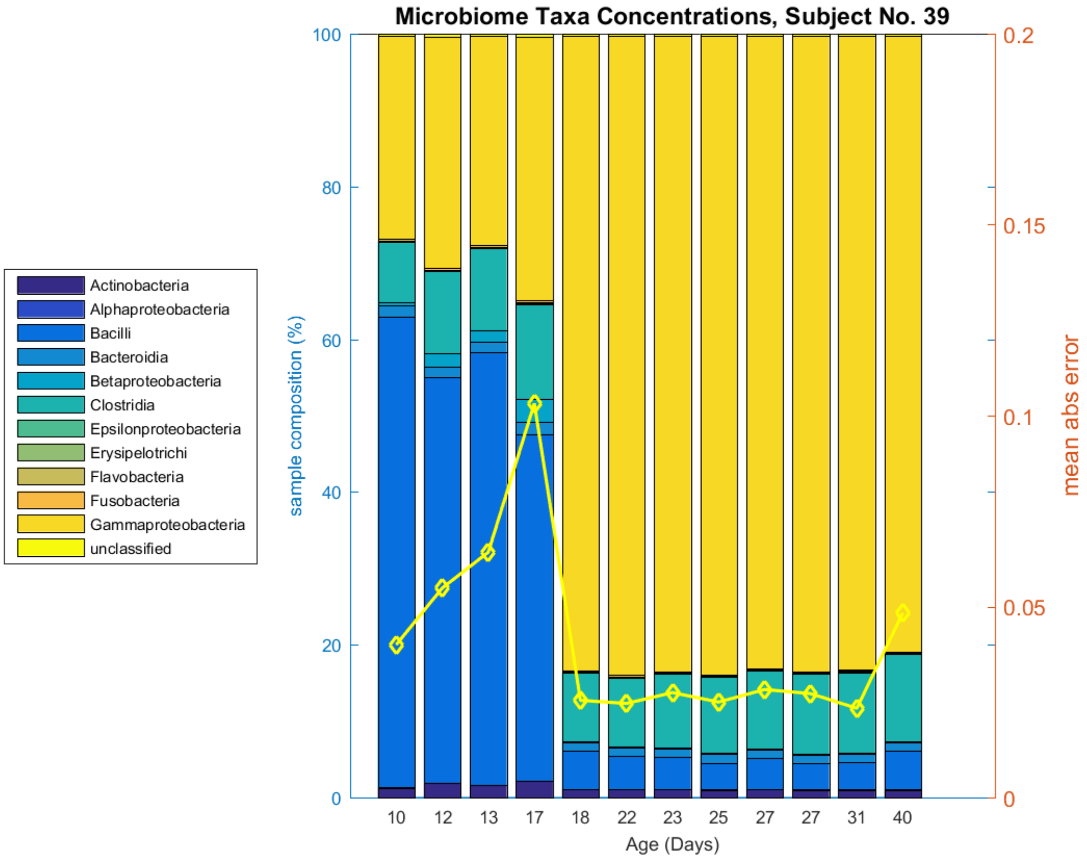
Microbiome Taxa Concentrations, Subject No. 39
Actinobacteria
Alphaproteobacteria
Bacilli
Bacteroidia
Betaproteobacteria
Clostridia
Epsilonproteobacteria
Erysipelotrichi
Flavobacteria
Fusobacteria
Gammaproteobacteria
unclassified
sample composition (%)
mean abs error
Age (Days)
10 12 13 17 18 22 23 25 27 27 31 40

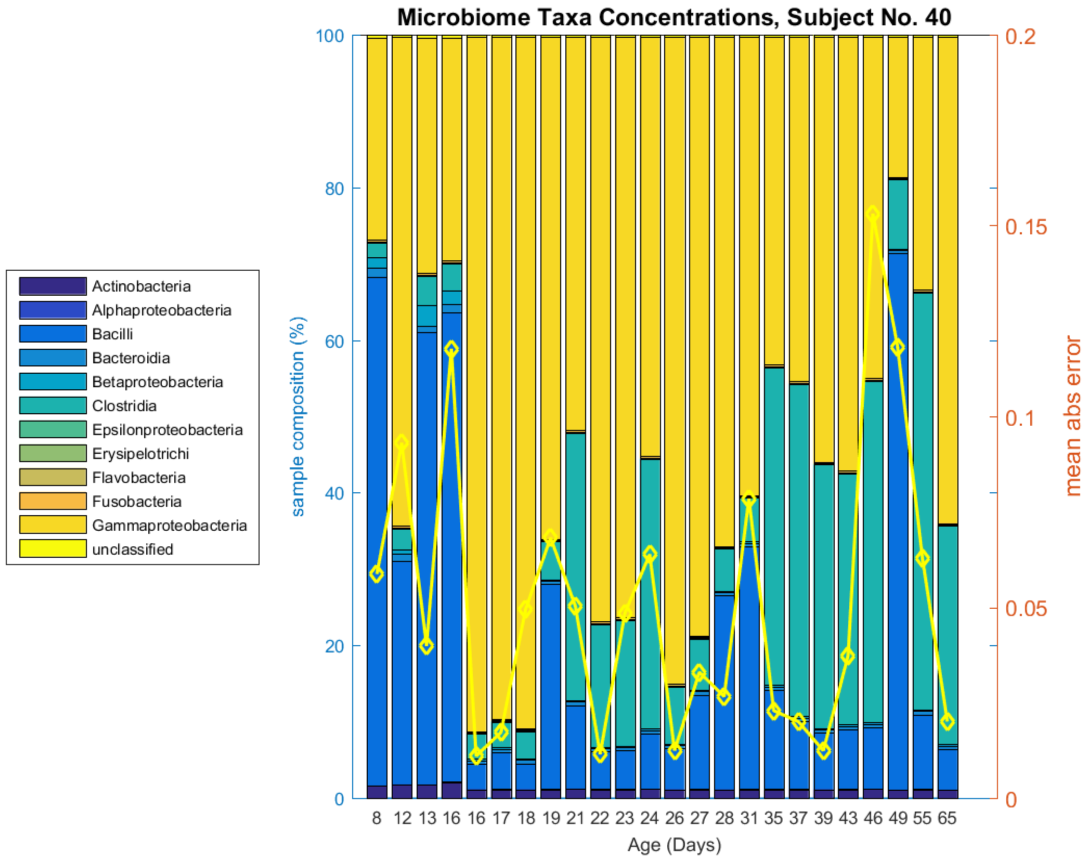
Microbiome Taxa Concentrations, Subject No. 40
Actinobacteria
Alphaproteobacteria
Bacilli
Bacteroidia
Betaproteobacteria
Clostridia
Epsilonproteobacteria
Erysipelotrichi
Flavobacteria
Fusobacteria
Gammaproteobacteria
unclassified
sample composition (%)
mean abs error
Age (Days)
8 12 13 16 16 17 18 19 21 22 23 24 26 27 28 31 35 37 39 43 46 49 55 65

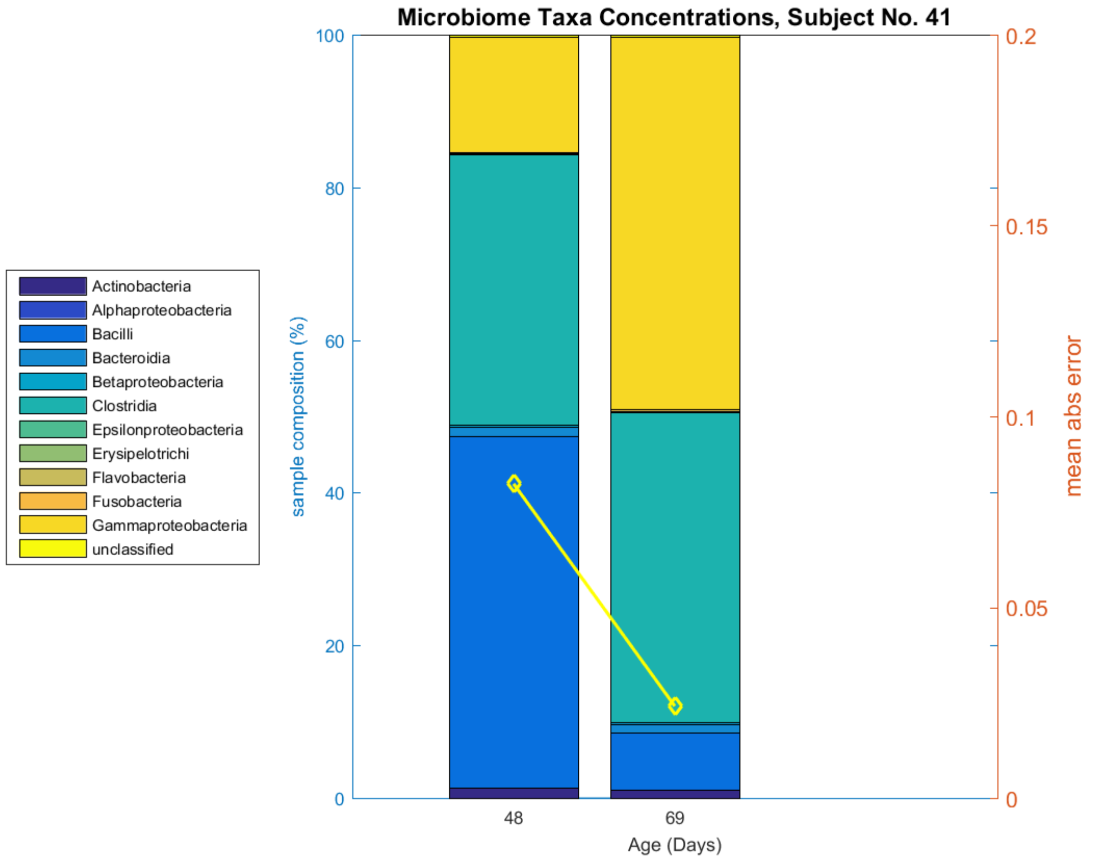

Microbiome Taxa Concentrations, Subject No. 41
sample composition (%)
mean abs error
Age (Days)
100
80
60
40
20
0
0.2
0.15
0.1
0.05
0
48
69
Actinobacteria
Alphaproteobacteria
Bacilli
Bacteroidia
Betaproteobacteria
Clostridia
Epsilonproteobacteria
Erysipelotrichi
Flavobacteria
Fusobacteria
Gammaproteobacteria
unclassified

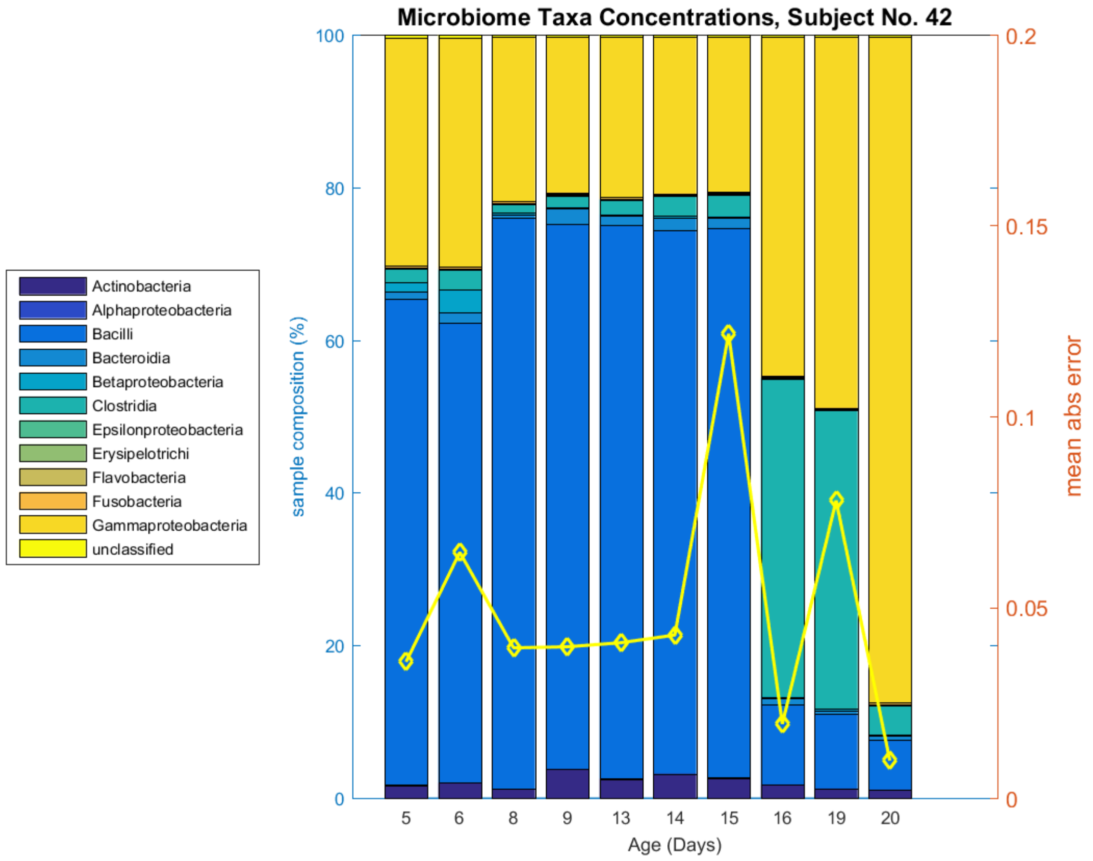

Microbiome Taxa Concentrations, Subject No. 42
Actinobacteria
Alphaproteobacteria
Bacilli
Bacteroidia
Betaproteobacteria
Clostridia
Epsilonproteobacteria
Erysipelotrichi
Flavobacteria
Fusobacteria
Gammaproteobacteria
unclassified
sample composition (%)
mean abs error
Age (Days)
100
80
60
40
20
0
0.2
0.15
0.1
0.05
0
5
6
8
9
13
14
15
16
19
20

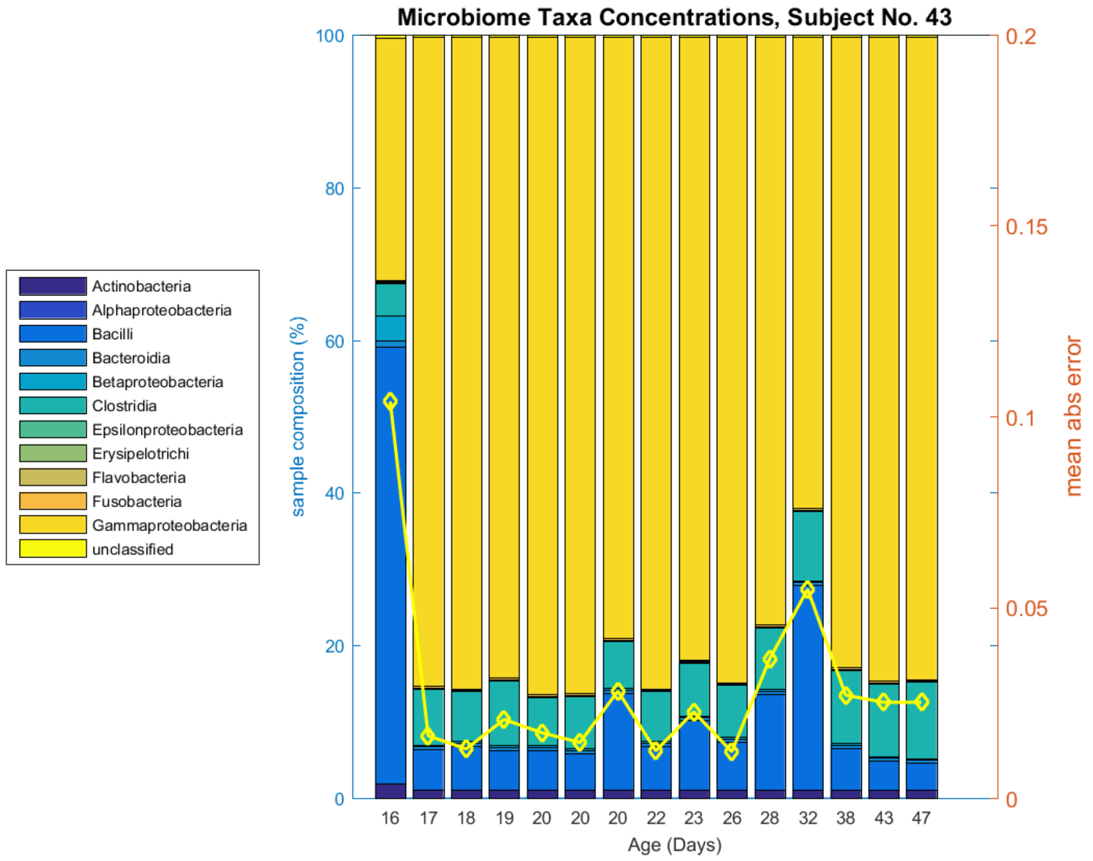
Microbiome Taxa Concentrations, Subject No. 43
Actinobacteria
Alphaproteobacteria
Bacilli
Bacteroidia
Betaproteobacteria
Clostridia
Epsilonproteobacteria
Erysipelotrichi
Flavobacteria
Fusobacteria
Gammaproteobacteria
unclassified
sample composition (%)
mean abs error
Age (Days)
16 17 18 19 20 20 20 22 23 26 28 32 38 43 47
0 20 40 60 80 100
0 0.05 0.1 0.15 0.2

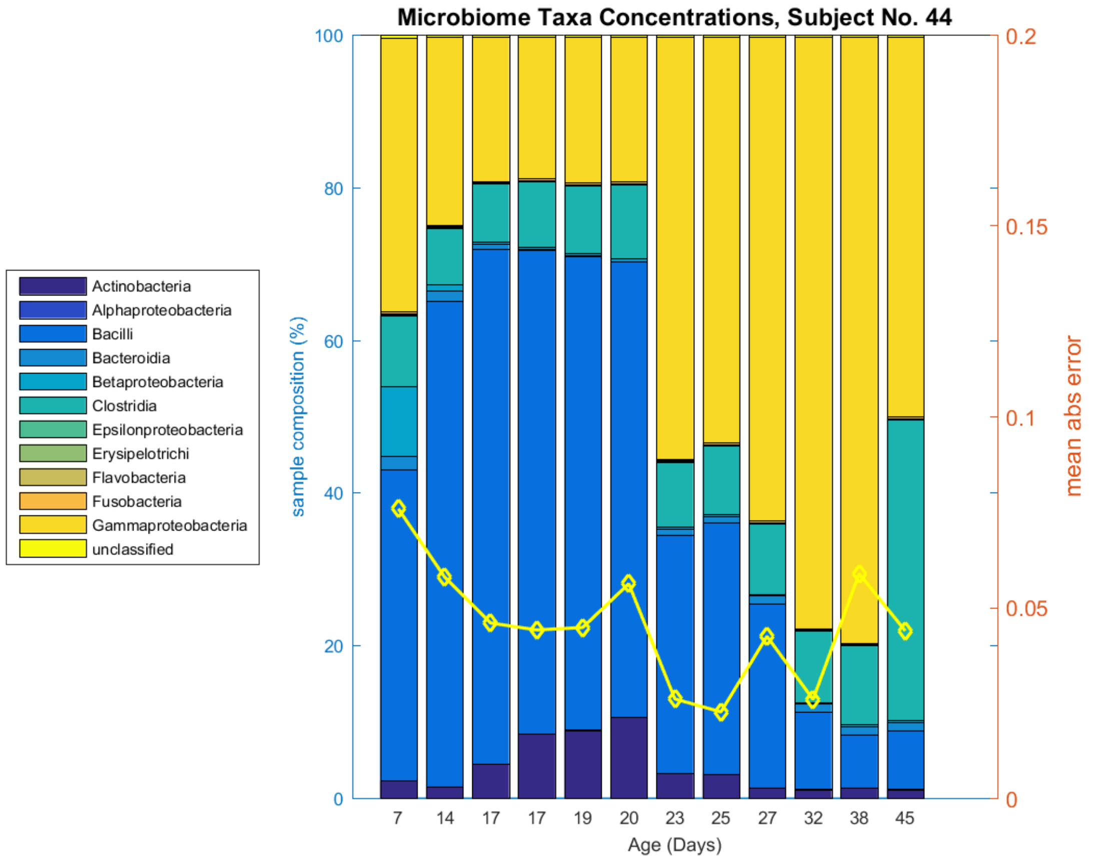

Microbiome Taxa Concentrations, Subject No. 44
Actinobacteria
Alphaproteobacteria
Bacilli
Bacteroidia
Betaproteobacteria
Clostridia
Epsilonproteobacteria
Erysipelotrichi
Flavobacteria
Fusobacteria
Gammaproteobacteria
unclassified
sample composition (%)
mean abs error
Age (Days)
0
20
40
60
80
100
0
0.05
0.1
0.15
0.2
7
14
17
17
19
20
23
25
27
32
38
45

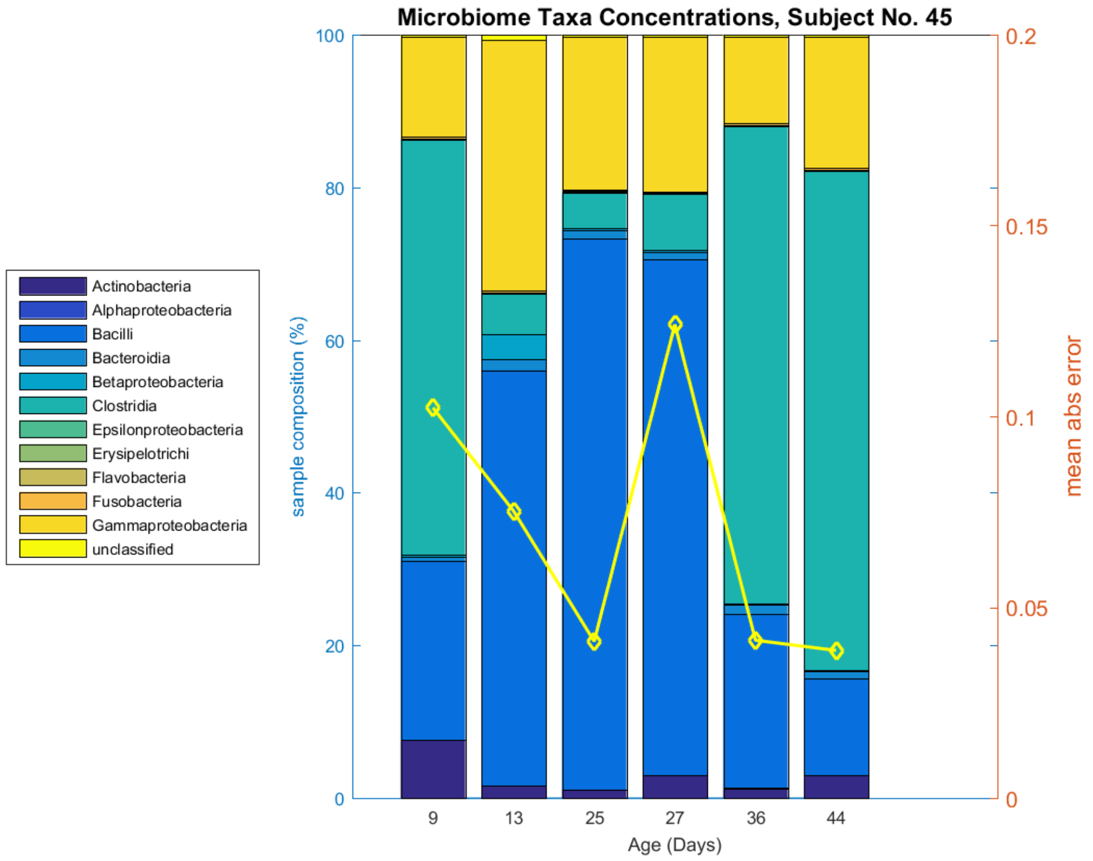
Microbiome Taxa Concentrations, Subject No. 45
Actinobacteria
Alphaproteobacteria
Bacilli
Bacteroidia
Betaproteobacteria
Clostridia
Epsilonproteobacteria
Erysipelotrichi
Flavobacteria
Fusobacteria
Gammaproteobacteria
unclassified
sample composition (%)
0
20
40
60
80
100
mean abs error
0
0.05
0.1
0.15
0.2
9
13
25
27
36
44
Age (Days)

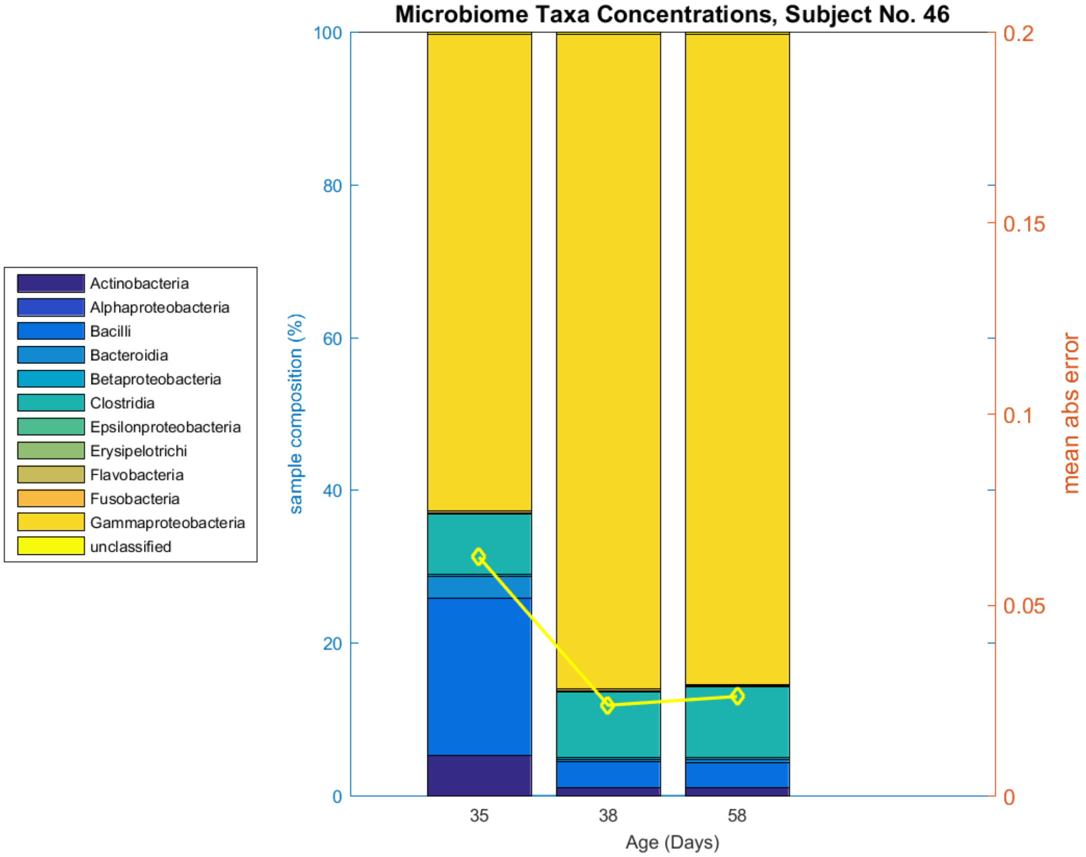
Microbiome Taxa Concentrations, Subject No. 46
Actinobacteria
Alphaproteobacteria
Bacilli
Bacteroidia
Betaproteobacteria
Clostridia
Epsilonproteobacteria
Erysipelotrichi
Flavobacteria
Fusobacteria
Gammaproteobacteria
unclassified
sample composition (%)
mean abs error
Age (Days)
100
80
60
40
20
0
0.2
0.15
0.1
0.05
0
35
38
58

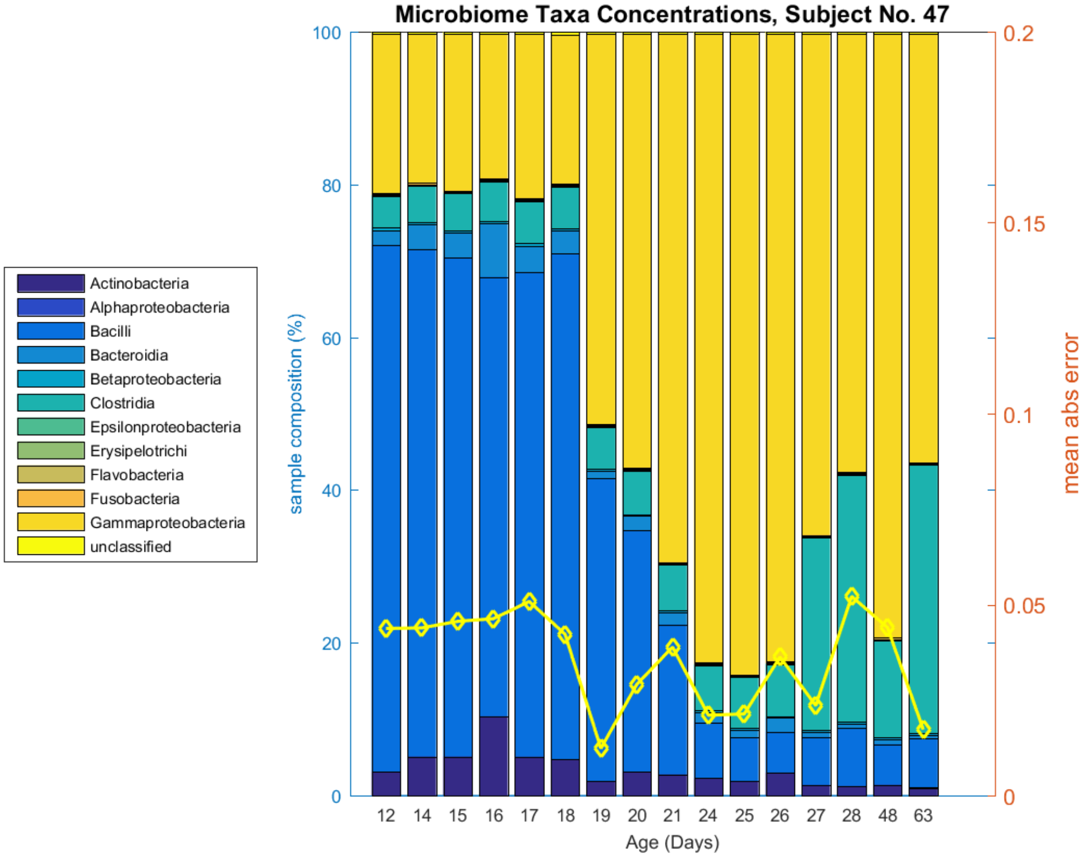
Microbiome Taxa Concentrations, Subject No. 47
Actinobacteria
Alphaproteobacteria
Bacilli
Bacteroidia
Betaproteobacteria
Clostridia
Epsilonproteobacteria
Erysipelotrichi
Flavobacteria
Fusobacteria
Gammaproteobacteria
unclassified
sample composition (%)
mean abs error
Age (Days)
12
14
15
16
17
18
19
20
21
24
25
26
27
28
48
63
0
20
40
60
80
100
0
0.05
0.1
0.15
0.2

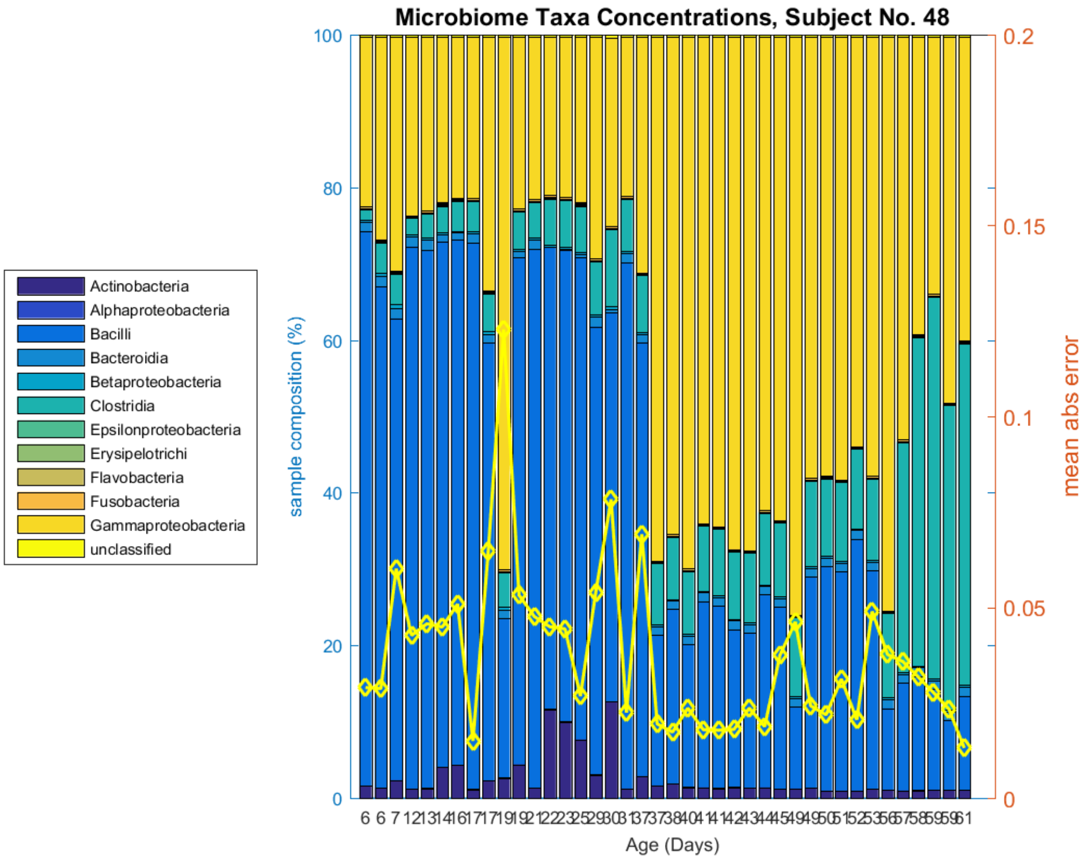

Microbiome Taxa Concentrations, Subject No. 48
Actinobacteria
Alphaproteobacteria
Bacilli
Bacteroidia
Betaproteobacteria
Clostridia
Epsilonproteobacteria
Erysipelotrichi
Flavobacteria
Fusobacteria
Gammaproteobacteria
unclassified
sample composition (%)
0
20
40
60
80
100
mean abs error
0
0.05
0.1
0.15
0.2
Age (Days)

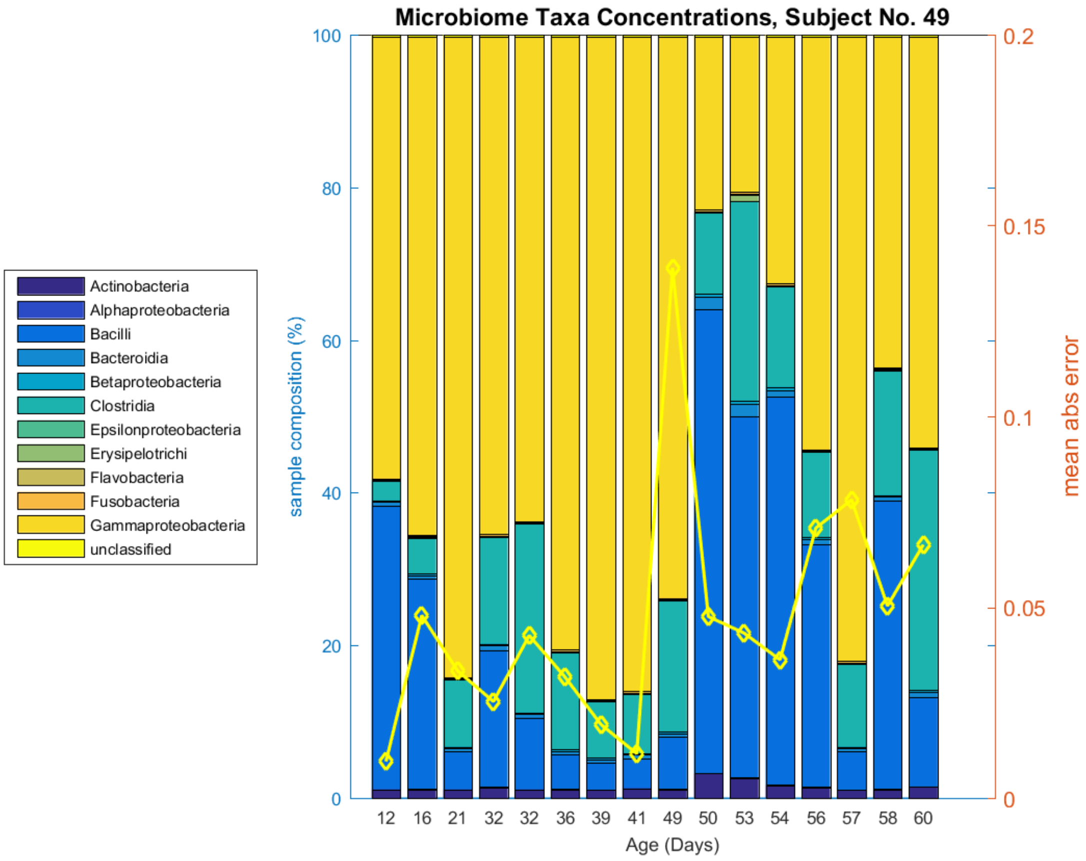

Microbiome Taxa Concentrations, Subject No. 49
Actinobacteria
Alphaproteobacteria
Bacilli
Bacteroidia
Betaproteobacteria
Clostridia
Epsilonproteobacteria
Erysipelotrichi
Flavobacteria
Fusobacteria
Gammaproteobacteria
unclassified
sample composition (%)
mean abs error
100
80
60
40
20
0
0.2
0.15
0.1
0.05
0
12 16 21 32 32 36 39 41 49 50 53 54 56 57 58 60
Age (Days)

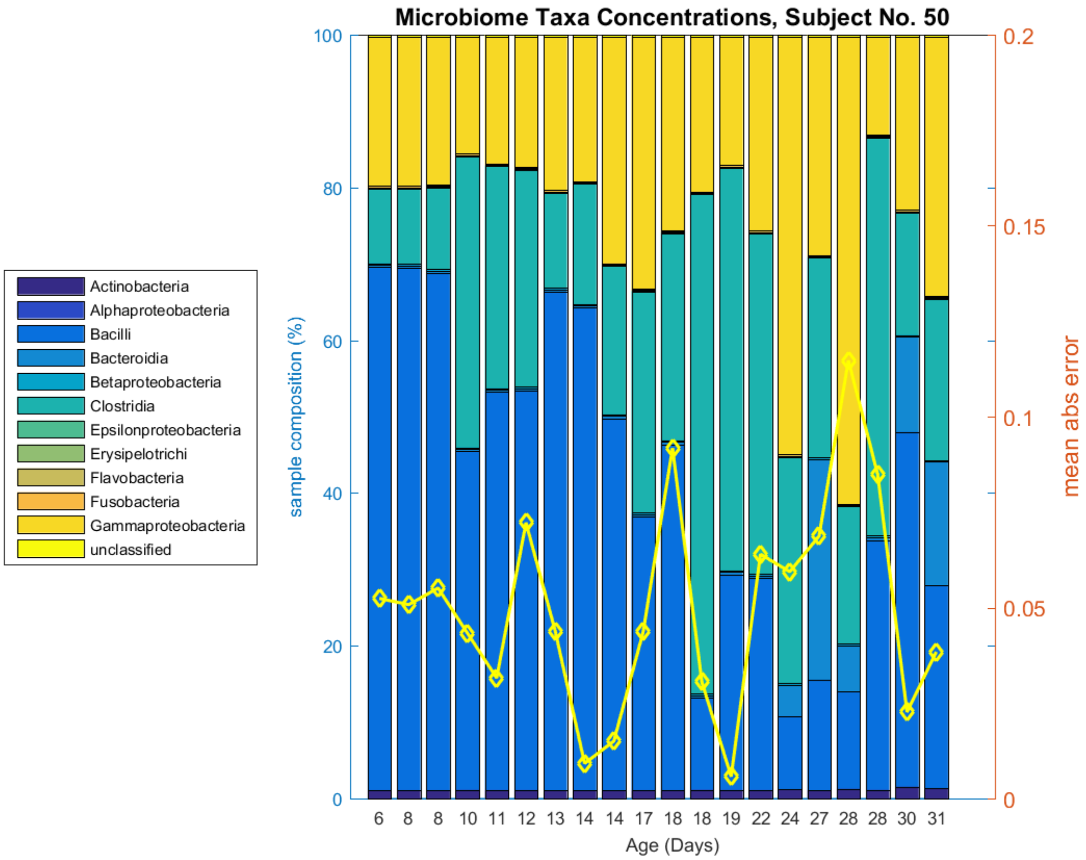

Microbiome Taxa Concentrations, Subject No. 50
Actinobacteria
Alphaproteobacteria
Bacilli
Bacteroidia
Betaproteobacteria
Clostridia
Epsilonproteobacteria
Erysipelotrichi
Flavobacteria
Fusobacteria
Gammaproteobacteria
unclassified
sample composition (%)
0
20
40
60
80
100
mean abs error
0
0.05
0.1
0.15
0.2
6 8 8 10 11 12 13 14 14 17 18 18 19 22 24 27 28 28 30 31
Age (Days)

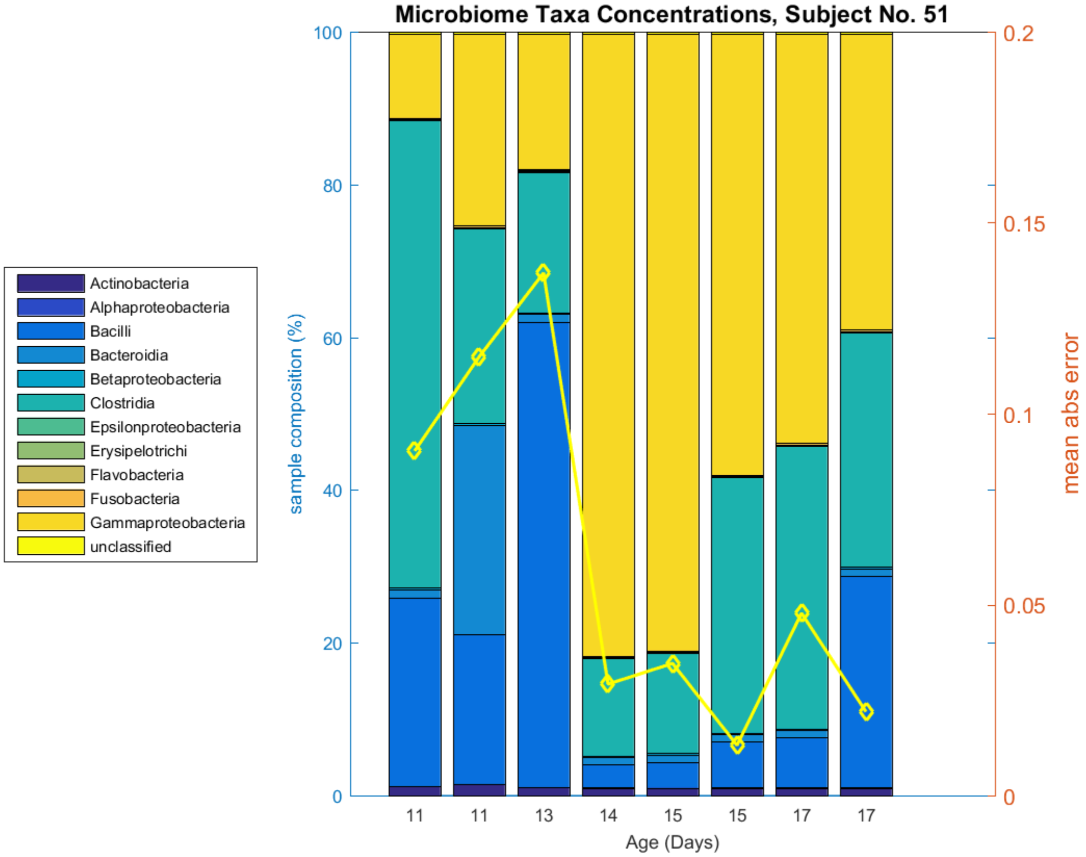
Microbiome Taxa Concentrations, Subject No. 51
Actinobacteria
Alphaproteobacteria
Bacilli
Bacteroidia
Betaproteobacteria
Clostridia
Epsilonproteobacteria
Erysipelotrichi
Flavobacteria
Fusobacteria
Gammaproteobacteria
unclassified
sample composition (%)
mean abs error
0
20
40
60
80
100
0
0.05
0.1
0.15
0.2
11
11
13
14
15
15
17
17
Age (Days)

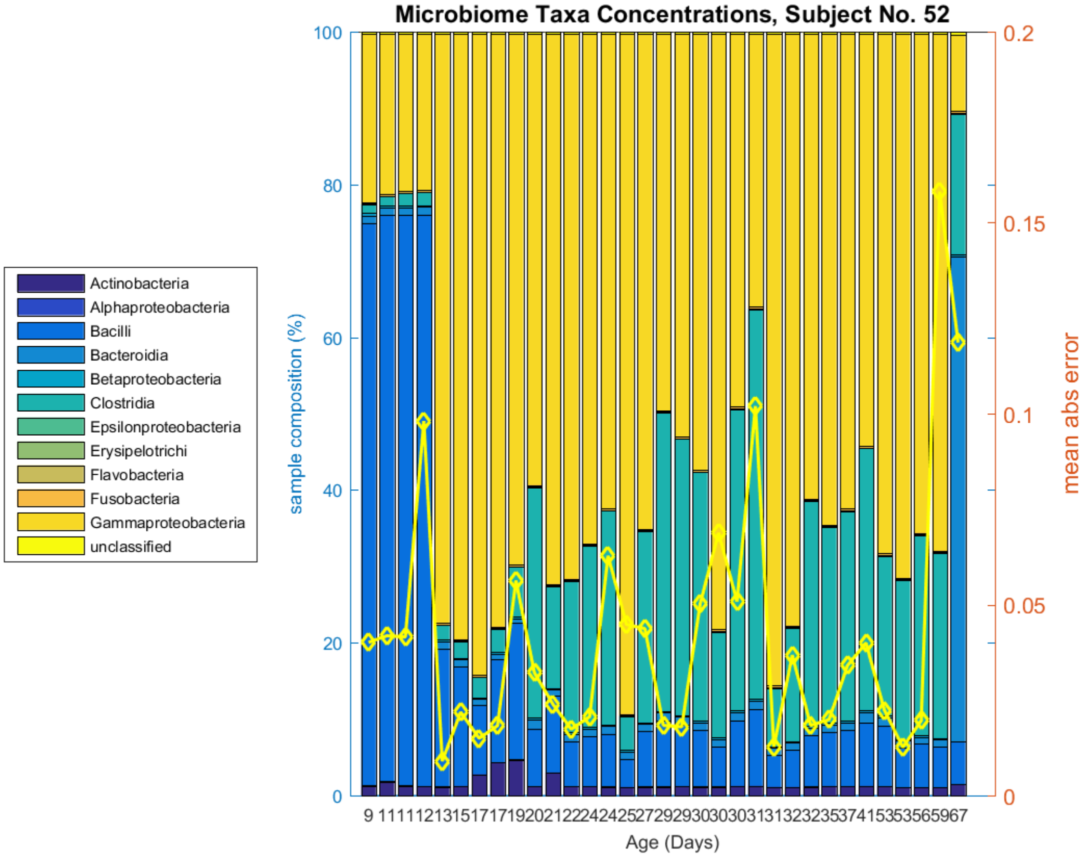
Microbiome Taxa Concentrations, Subject No. 52
Actinobacteria
Alphaproteobacteria
Bacilli
Bacteroidia
Betaproteobacteria
Clostridia
Epsilonproteobacteria
Erysipelotrichi
Flavobacteria
Fusobacteria
Gammaproteobacteria
unclassified
sample composition (%)
mean abs error
Age (Days)
100
80
60
40
20
0
0.2
0.15
0.1
0.05
0
9 11111121315171719202122242425272929303030313132323537415353565967

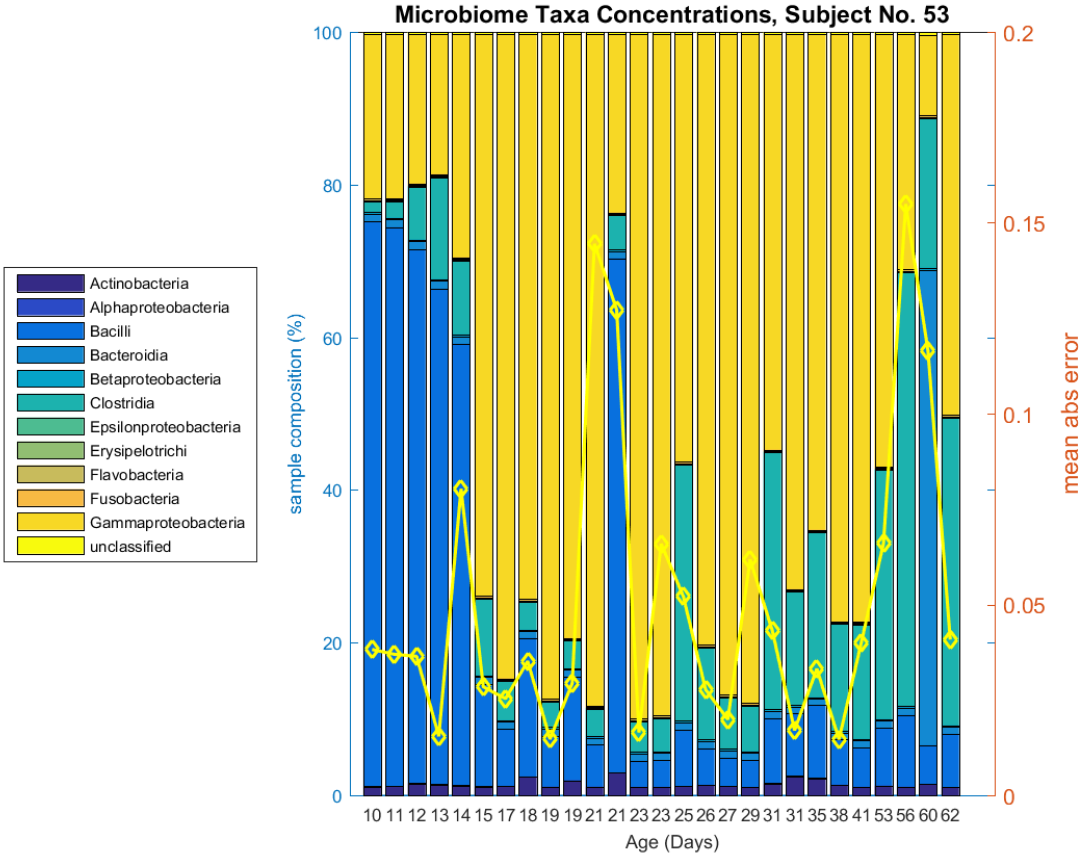
Microbiome Taxa Concentrations, Subject No. 53
Actinobacteria
Alphaproteobacteria
Bacilli
Bacteroidia
Betaproteobacteria
Clostridia
Epsilonproteobacteria
Erysipelotrichi
Flavobacteria
Fusobacteria
Gammaproteobacteria
unclassified
sample composition (%)
mean abs error
0
20
40
60
80
100
0
0.05
0.1
0.15
0.2
10 11 12 13 14 15 17 18 19 19 21 21 23 23 25 26 27 29 31 31 35 38 41 53 56 60 62
Age (Days)

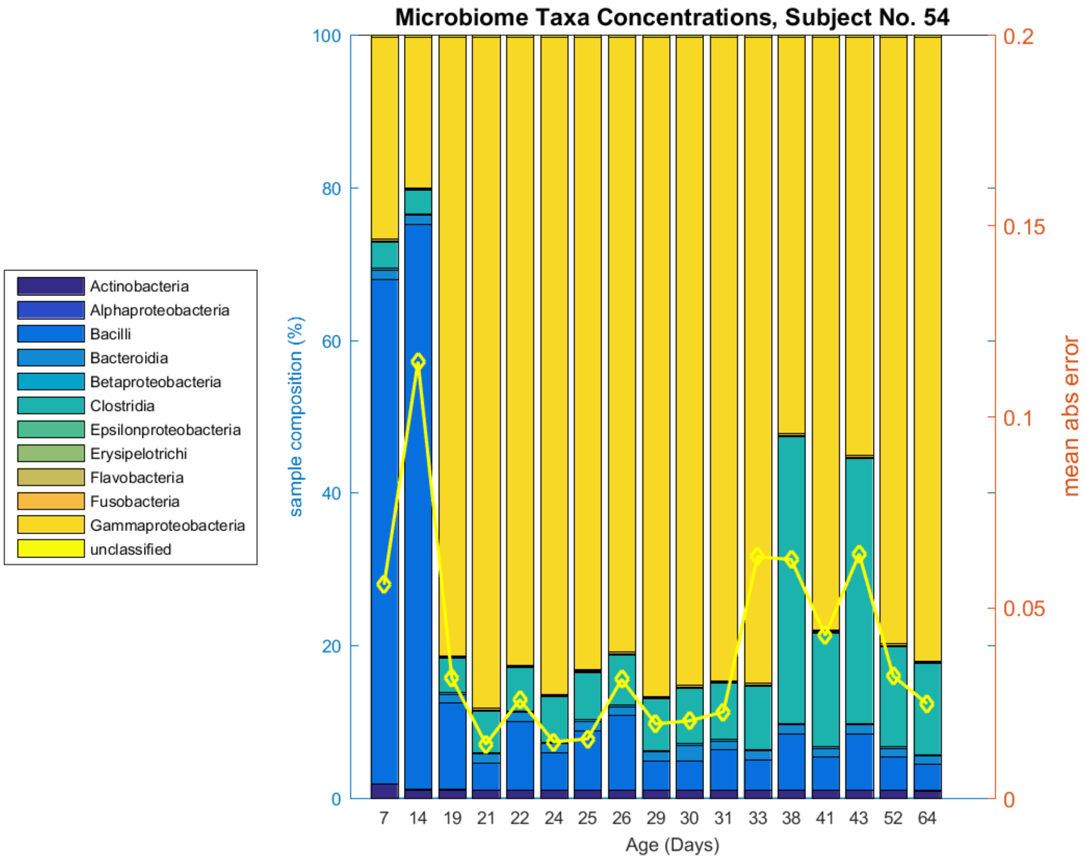
Microbiome Taxa Concentrations, Subject No. 54
Actinobacteria
Alphaproteobacteria
Bacilli
Bacteroidia
Betaproteobacteria
Clostridia
Epsilonproteobacteria
Erysipelotrichi
Flavobacteria
Fusobacteria
Gammaproteobacteria
unclassified
sample composition (%)
mean abs error
Age (Days)
0
20
40
60
80
100
0
0.05
0.1
0.15
0.2
7 14 19 21 22 24 25 26 29 30 31 33 38 41 43 52 64

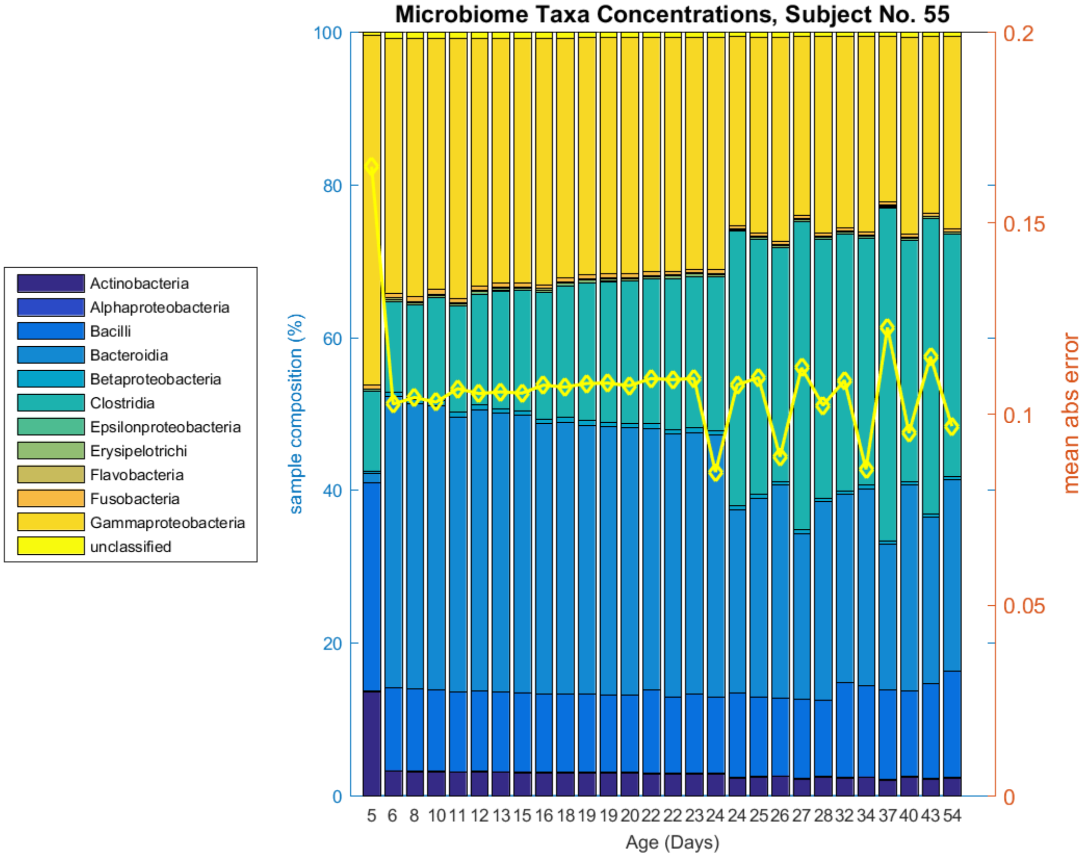

Microbiome Taxa Concentrations, Subject No. 55
Actinobacteria
Alphaproteobacteria
Bacilli
Bacteroidia
Betaproteobacteria
Clostridia
Epsilonproteobacteria
Erysipelotrichi
Flavobacteria
Fusobacteria
Gammaproteobacteria
unclassified
sample composition (%)
mean abs error
Age (Days)
5 6 8 10 11 12 13 15 16 18 19 19 20 22 22 23 24 24 25 26 27 28 32 34 37 40 43 54

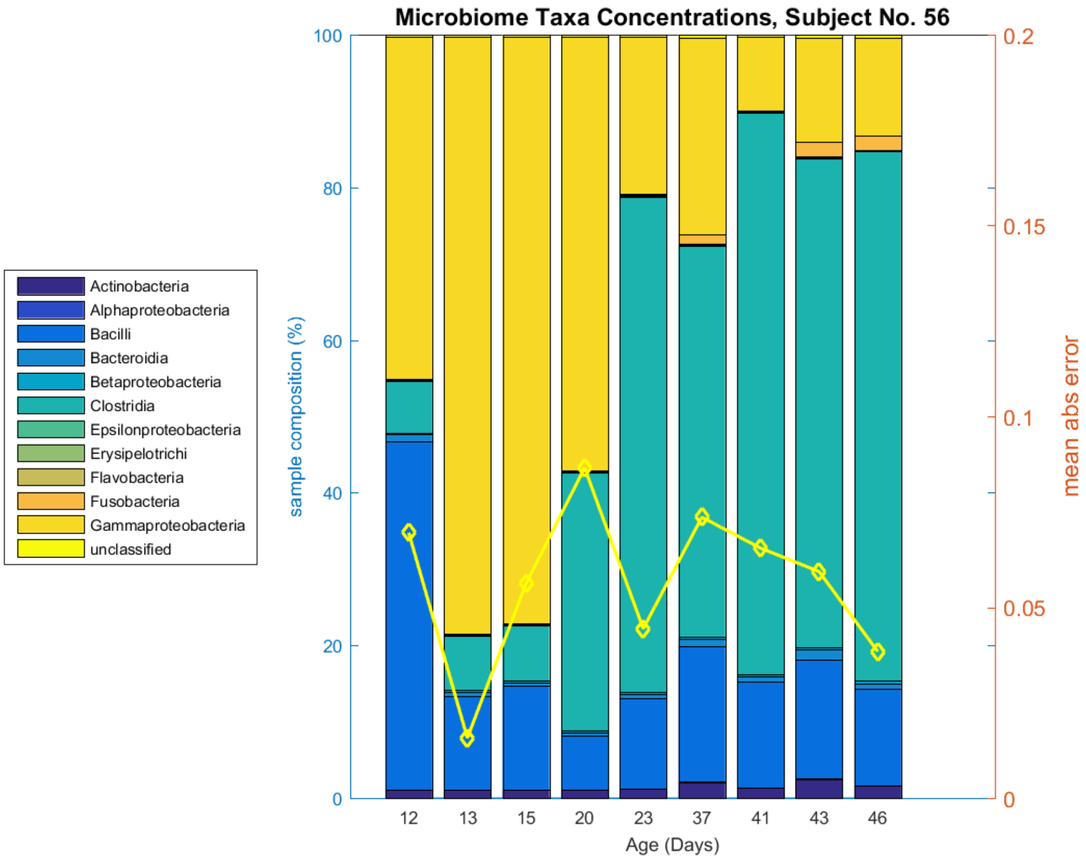
Microbiome Taxa Concentrations, Subject No. 56
Actinobacteria
Alphaproteobacteria
Bacilli
Bacteroidia
Betaproteobacteria
Clostridia
Epsilonproteobacteria
Erysipelotrichi
Flavobacteria
Fusobacteria
Gammaproteobacteria
unclassified
sample composition (%)
mean abs error
Age (Days)
12
13
15
20
23
37
41
43
46
0
20
40
60
80
100
0
0.05
0.1
0.15
0.2

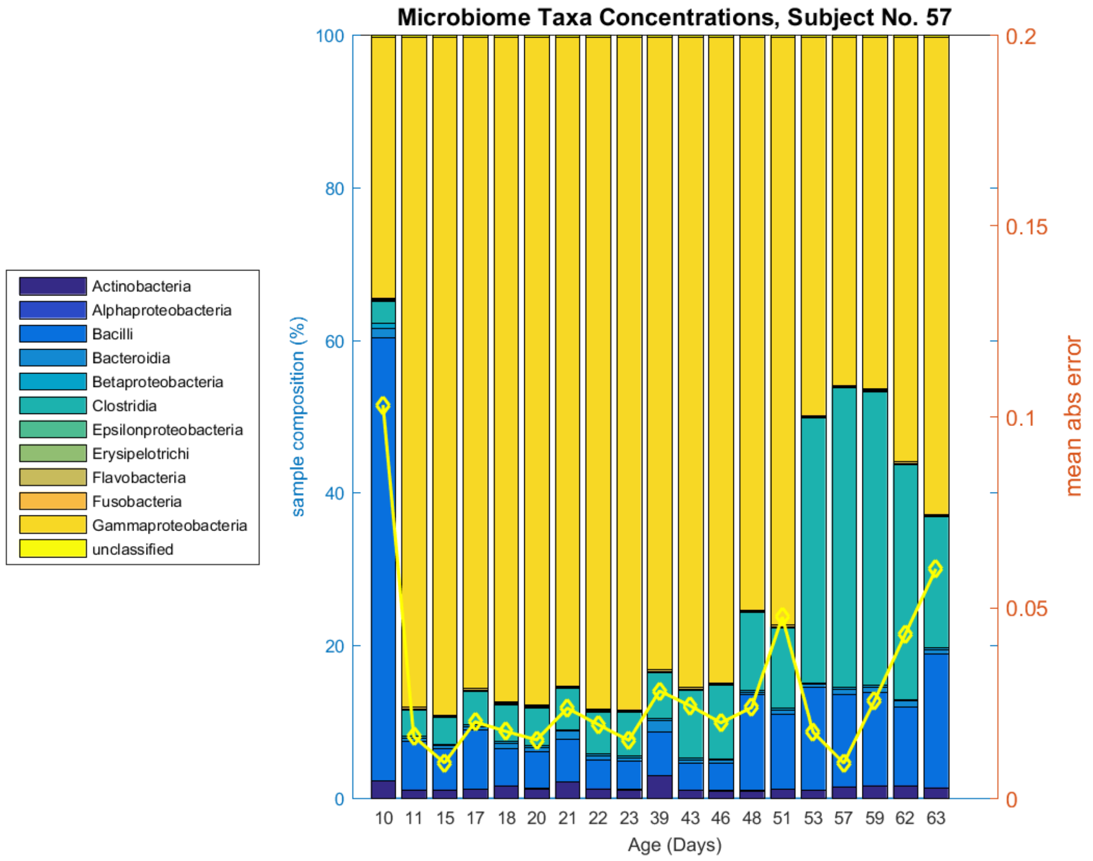

Microbiome Taxa Concentrations, Subject No. 57
Actinobacteria
Alphaproteobacteria
Bacilli
Bacteroidia
Betaproteobacteria
Clostridia
Epsilonproteobacteria
Erysipelotrichi
Flavobacteria
Fusobacteria
Gammaproteobacteria
unclassified
sample composition (%)
0
20
40
60
80
100
mean abs error
0
0.05
0.1
0.15
0.2
10 11 15 17 18 20 21 22 23 39 43 46 48 51 53 57 59 62 63
Age (Days)

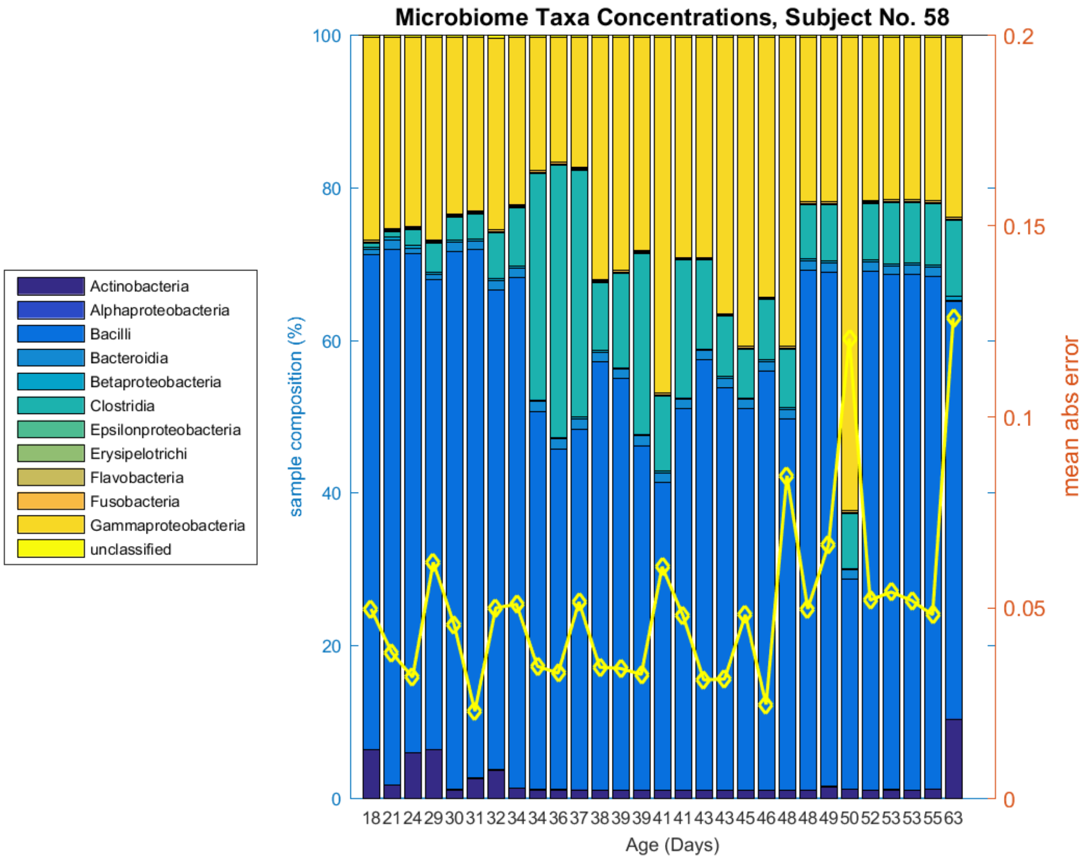

Microbiome Taxa Concentrations, Subject No. 58
Actinobacteria
Alphaproteobacteria
Bacilli
Bacteroidia
Betaproteobacteria
Clostridia
Epsilonproteobacteria
Erysipelotrichi
Flavobacteria
Fusobacteria
Gammaproteobacteria
unclassified
sample composition (%)
mean abs error
Age (Days)
0
20
40
60
80
100
0
0.05
0.1
0.15
0.2
18 21 24 29 30 31 32 34 34 36 37 38 39 39 41 41 43 43 45 46 48 48 49 50 52 53 53 55 63
